# Synthesis and Characterization of a New Series of Bis(allylic-α-aminophosphonates) under Mild Reaction Conditions

**DOI:** 10.3390/molecules28124678

**Published:** 2023-06-09

**Authors:** Ichrak Souii, Med Abderrahmane Sanhoury, Javier Vicario, Xabier Jiménez-Aberásturi, Mohamed L. Efrit, Hedi M’rabet, Jesús M. de los Santos

**Affiliations:** 1 Laboratory of Selective Organic & Heterocyclic Synthesis Biological Activity Evaluation (LR17ES01), Department of Chemistry, Faculty of Sciences of Tunis, University of Tunis El Manar, Tunis 2092, Tunisia; medlotfi.efrit@fst.utm.tn (M.L.E.); hedi.mrabet@fst.utm.tn (H.M.); 2 Department of Organic Chemistry I, Faculty of Pharmacy and Lascaray Research Center, University of the Basque Country (UPV/EHU), Paseo de la Universidad 7, 01006 Vitoria, Spain; javier.vicario@ehu.eus (J.V.); xabier.jimenezdeaberasturi@ehu.eus (X.J.-A.); 3 Laboratory of Structural Organic Chemistry: Synthesis and Physicochemical Studies, Department of Chemistry, Faculty of Sciences of Tunis, University of Tunis El Manar, El Manar I, Tunis 2092, Tunisia; senhourry@yahoo.com

**Keywords:** bis(α-aminophosphonates), bis(allylic-α-aminophosphonates), multicomponent Kabachnik–Fields reaction, nucleophilic substitution reaction

## Abstract

Several bis(α-aminophosphonates) have been conveniently prepared in good yields using a straightforward multicomponent Kabachnik–Fields reaction between ethane 1,2-diamine or propane 1,3-diamine, diethylphosphite and aldehydes under catalyst-free conditions. The nucleophilic substitution reaction of bis(α-aminophosphonates) prepared and ethyl (2-bromomethyl)acrylate under mild reaction conditions afforded an original synthetic approach to a new series of bis(allylic-α-aminophosphonates).

## 1. Introduction

Bisphosphonates (BPs) are a well-known family of organophosphorus compounds [1] used as the treatment of choice for metastatic malignant and osteoporosis diseases [2,3,4]. α-Aminophosphonates represent an important family of organophosphorus compounds [5] due to their classification as structurally equivalent to α-amino acids. They exhibit an extensive range of activities [6,7] covering antitumor [8,9], anti-HIV [10], antibiotics [11], fungicides [12] and herbicides [13]. Owing to their multidentate structures [14], they can be used as ligands for metal extraction [15,16,17] and as monomers for the synthesis of polymeric materials [18]. Furthermore, they can be suitable for the modification of polymeric structures [19] and can serve the production of *P*-macrocyclic compounds [20]. In this strategy, allylic amines are considered as an efficient class of organic compounds. They are valuable intermediates in synthetic chemistry [21] and highly synthetic targets for biologically diverse active amine derivatives [22,23].

Multicomponent reactions (MCRs) [24,25,26,27] are processes where more than two substrates react together in a one-pot chemical to form a single product that contains significant portions of all the starting materials. In the context of green chemistry, these multicomponent approaches have many benefits in comparison with stepwise reactions, including the high atom economy, their step efficiency, high exploratory power and convergence, and these processes avoid the need to purify the intermediate products. In this sense, they are perfectly suited to environmentally friendly synthesis and they are considered to be crucial synthetic tools in diversity-oriented synthesis [28,29].

In this context, and among the various methods used for the synthesis of aminophosphonates, Kabachnik–Fields reaction represents a powerful methodology via a multicomponent condensation reaction [30,31]. For instance, in 2014, Mulla et al. [32] reported a highly efficient one-pot multicomponent synthesis of α-aminophosphonates and bis(α-aminophosphonates) catalyzed using heterogeneous reusable silica-supported dodecatungstophosphoric acid at ambient temperature and their antitubercular evaluation against mycobactrium tuberculosis. Recently, Aissa et al. [33] described the hydrophosphinylation with double Kabachnik–Fields reaction through one-pot condensation of diamine, aromatic aldehyde and diethylphosphite for the preparation of bis(α-aminophosphonates) with excellent diastereoselectivity and chemical yields, using the organic Fiaud’s acid as a chiral Brønsted acid catalyst.

As a part of our ongoing research on the chemistry of β-ethoxycarbonyl allylphosphonates, we recently described the synthesis of variously substituted compounds via a 1,3-dipolar cycloaddition reaction of β-ethoxycarbonyl *N*-methylphosphonated allylic amines, used as dipolarophiles, with chloroximes under mild reaction conditions [34]. As a continuation of our interest on this topic, we aim to investigate and possibly increase the activities of the bis(α-aminophosphonates) and allylic amines through the coupling of both moieties. According to this, herein we report the first synthetic method leading to novel bis(allylic-α-aminophosphonic esters) **6**, via a simple coupling reaction between ethyl 2-bromomethylacrylate **5** and bis(α-aminophosphonates) **4**, prepared using an eco-friendly and cost effective straightforward multicomponent Kabachnik–Fields reaction.

## 2. Results and Discussion

Firstly, we performed the synthesis of bis(α-aminophosphonate) **4a** using a straightforward three-component condensation of ethane 1,2-diamine **1a** (*n* = 2), diethylphosphite **2** and benzaldehyde **3a** (R = C_6_H_5_). Remarkably, this synthetic method was carried out under catalyst-free conditions. The mixture was refluxed for 20 h in ethanol as a green solvent (method A) or during 5 to 6 h in dry toluene (method B). Although both reaction conditions lead selectively to bis(α-aminophosphonate) **4a** in good yields and without the presence of any other byproducts, a slightly higher yield was obtained in this case when toluene was used as the solvent (Table 1, Entry 1). Next, both procedures were extended to the use of *p*-tolualdehyde **3b** (R = *p*-MeC_6_H_4_), affording again in both cases, exclusively, bis(α-aminophosphonate) **4b** in similar yields. However, a significantly higher yield was observed in this case when ethanol was used as the solvent (Table 1, Entry 2).

With the aim to extend the scope of our multicomponent reaction, next we tested the same synthetic procedures using propane 1,3-diamine **1b** (*n* = 3), diethylphosphite **2** and benzaldehyde **3a** (R = C_6_H_5_), obtaining bis(α-aminophosphonate) **4c** in good yields and observing a slightly higher efficiency in the protocol using toluene as the solvent (Table 1, Entry 3). Moreover, the synthetic procedure using propane 1,3-diamine **1b** (*n* = 3) and diethylphosphite **2** was extended to the use of aromatic aldehydes bearing electron withdrawing groups such as *p*-tolualdehyde (**3b**, R = *p*-MeC_6_H_4_) and *p*-anisaldehyde (**3c**, R = *p*-MeOC_6_H_4_) or halogen-substituted benzaldehydes, such as *p*-chlorobenzaldehyde (**3d**, R = *p*-ClC_6_H_4_) and *p*-fluorobenzaldehyde (**3e**, R = *p*-FC_6_H_4_), obtaining bis(α-aminophosphonates) **4d**–**g** in good to excellent yields (Table 1, Entries 4–7). Finally, more challenging aliphatic enolizable acetaldehyde **3f** (R = Me) was selected as the electrophilic partner of the reaction obtaining aliphatic substituted bis(α-aminophosphonates) **4h** with excellent results using both procedures (Table 1, Entry 8).

Bis(α-aminophosphonates) **4** were characterized on the basis of their ^1^H, ^31^P and ^13^C NMR spectra. Functionalized bis(α-aminophosphonate) **4g** was chosen in order to unequivocally establish the structure of the reaction products obtained in the Kabachnik–Fields three-component reaction. In ^31^P NMR spectrum of compound **4g**, the phosphonate group resonates at *δ*_P_ = 22.9 ppm, while in ^19^F NMR spectrum only a signal at *δ*_F_ *=* −115.0 ppm is observed. One of the most characteristic signals in the ^1^H NMR spectrum, which evidences the formation of **4g**, corresponds to the two methylene groups directly bonded to both nitrogen atoms at *δ*_H_ = 2.23 ppm that integrates four protons, and the methylene bridge of compound **4g**, which resonates at *δ*_H_ = 1.30 ppm. As expected, both signals proved to be coupled in the homonuclear correlation spectroscopy (COSY) spectrum (see Supplementary Materials). Remarkably, the signal corresponding to both NH groups of **4g** appears at *δ*_H_ = 1.70 ppm and shows a slow interchange with D_2_O. In addition, the ^13^C NMR spectrum shows a signal at *δ*_C_ = 161.5 ppm as a well-resolved doublet for the aromatic quaternary carbon directly connected to fluorine atom, with a typical *ipso* coupling constant (^1^*J*_CF_ = 245.8 Hz), and another doublet at *δ*_C_ = 59.6 ppm for the tertiary carbon bonded to the phosphorus atom with a coupling constant of ^1^*J*_PC_ = 153.3 Hz. Some other characteristic signals in the ^13^C NMR spectrum of **4g** correspond to the two methylene groups directly bonded to both nitrogen atoms that appear as a doublet at *δ*_C_ = 45.1 ppm (^3^*J*_PC_ = 17.3 Hz) as well as the methylene bridge, whose presence is evident from the singlet at *δ*_C_ = 29.0 ppm. The proton and carbon signals assigned to the methylene groups show a correlation in the heteronuclear single quantum coherence spectroscopy (HSQC) spectrum (see Supplementary Materials).

**Scheme 1:**
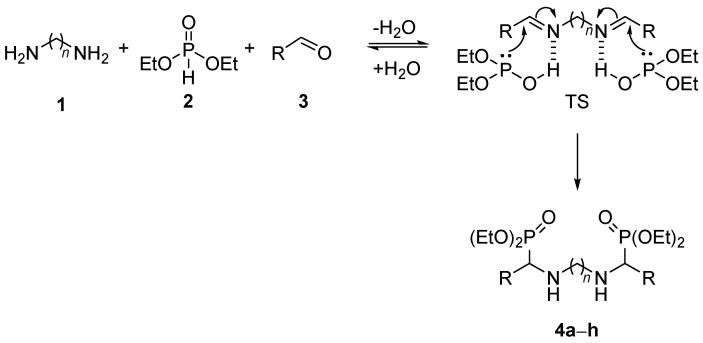
Proposed mechanism for the Kabachnik–Fields condensation reaction.

The condensation of primary diamine **1**, aldehyde **2** and a diethyl phosphite **3** was presumed to follow the “imine” mechanism (Scheme 1). First, the double condensation between diamine and two equivalents of aldehyde affords a di-imine species and next, a double hydrogen bond is formed between both imine nitrogen atoms and the P-OH of two units of the phosphite tautomer as shown in TS. Under this conformation, the nucleophilic lone pair of electrons at the phosphorus atoms attack the electrophilic imine moiety leading the formation of bis(α-aminophosphonates) **4**.

In order to manifest the synthetic utility of the substrates obtained from the multicomponent reaction, next we used our bis(α-aminophosphonates) **4** as starting materials in a reaction comprising the first simultaneous generation of symmetric double allylic-α-aminophosphonate moieties. This novel approach was undertaken using a simple double nucleophilic substitution of bis(α-aminophosphonates) (**4a**–**h**) with ethyl (2-bromomethyl)acrylate **5** under mild reaction conditions. We first explored the reaction of bis(α-aminophosphonate) **4a** (R = C_6_H_5_) with two equivalents of ethyl (2-bromomethyl)acrylate **5** in THF as solvent and in the absence of any base. Under these reaction conditions, no reaction progress was observed. Nevertheless, when the reaction was performed in THF with 2.5 equivalents of trimethylamine, bis(allylic-α-aminophosphonate) **6a** was obtained in good yield after 12 h at room temperature (Table 2, Entry 1). Likewise, the reaction was achieved using trimethylamine as base in acetonitrile or in ethyl ether as solvents. In these cases, **6a** was isolated in only 30% and 45% chemical yield, respectively. With the optimal conditions in hand, the scope of this reaction was evaluated with a series of bis(α-aminophosphonic esters) **4** obtained from our multicomponent protocols. As illustrated in Table 2, the synthetic pathway, which was successfully extended to the preparation of a series of bis(allylic-α-aminophosphonates) **6a**–**h**, is tolerant to a variety of substitution, including not only bis(allylic-α-aminophosphonates) derived from ethylenediamine (**6a**–**b**, Table 2, Entries 1–2) but also those derived from propylenediamine (**6c**–**h**, Table 2, Entries 3–8). Moreover, the reaction procedure is equally efficient when different aromatic substituents are present, such as *p*-tolyl, *p*-anisyl, *p*-chlorophenyl or *p*-fluorophenyl (Table 2, Entries 4–7) and even when aliphatic substituted substrates are used (Table 2, Entry 8).

Substrates obtained from the nucleophilic substitution of bis(α-aminophosphonates) **4** with ethyl (2-bromomethyl)acrylate **5** were fully characterized on the basis of their ^1^H, ^31^P and ^13^C NMR spectra and HRMS (see Supplementary Materials). For instance, bis(allylic-α-aminophosphonate) **6g** showed one absorption at *δ*_P_ = 23.4 ppm in the 31P NMR spectrum, while in its ^19^F NMR spectrum, only a signal at *δ*_F_ *=* −114.2 ppm was observed. One of the most representative signals that appear in the ^1^H NMR spectrum of **6g** are those corresponding to the methylene group of the terminal alkene at *δ*_H_ *=* 6.28 and 5.93 ppm. Furthermore, both signals showed a cross-peak in the homonuclear correlation spectroscopy (COSY) spectrum (see Supplementary Materials). Likewise, in the ^13^C NMR spectrum of compound **6g**, certainly, the well-resolved doublet corresponding to the tertiary carbon at *δ*_C_ *=* 61.0 ppm, which shows a very strong *ipso*-coupling with the phosphorus atom (^1^*J*_PC_ = 159.4 Hz), is the most remarkable signal. The presence of the ester group is evident from the chemical shift at *δ*_C_ *=* 166.7 ppm, typical for carboxylic groups. The quaternary carbon and the methylene group of the C=C double bond appear at *δ*_C_ *=* 138.4 and 126.0 ppm, respectively. All the proton and carbon signals assigned show a correlation in the heteronuclear single quantum coherence spectroscopy (HSQC) spectrum (see Supplementary Materials).

In agreement with the structure proposed for **6g**, the heteronuclear multiple-bond correlation spectroscopy (HMBC) spectrum presents cross-peak of both protons of the terminal alkene (*δ*_H_ *=* 6.28 and 5.93 ppm) with the quaternary C–C double bond (*δ*_C_ *=* 138.4 ppm), the carbonyl group (*δ*_C_ *=* 166.7 ppm) and the methylene group directed bonded to the nitrogen atom (*δ*_C_ *=* 51.7 ppm).

**Scheme 2:**
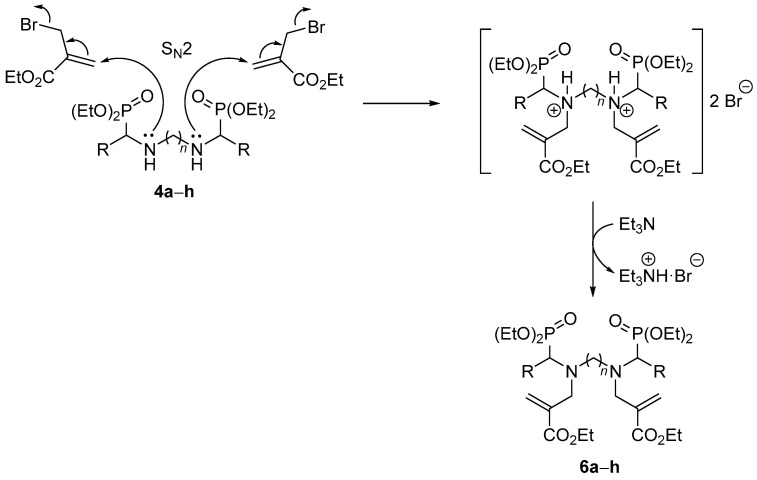
Suggested mechanism for the double nucleophilic substitution of bis(α-aminophosphonates) **4** with ethyl (2-bromomethyl)acrylate **5**.

The reaction mechanism suggests a double bimolecular nucleophilic substitution (S_N_2) reaction through the attack of the lone pair of electrons on the nitrogen atoms of compound **4** to the C=C double bond of ethyl (2-bromomethyl)acrylate **5**. Further concomitant removal of the leaving group would afford an intermediate salt, which after base treatment would yield bis(allylic-α-aminophosphonates) **6** (Scheme 2).

We finally explore if this process might be performed in a one-pot operation that would be appealing from an atom-economic alternative for carbon–heteroatom bond construction. Therefore, a mixture of propane 1,3-diamine (**1b**), diethylphosphite (**2**) and *p*-fluorobenzaldehyde (**3e**) was refluxed in EtOH for 20 h. After cooling and removing the solvent, the crude compound, without isolation, was subjected to double nucleophilic substitution conditions. The crude compound was dissolved in THF, and 2 equivalents of ethyl (2-bromomethyl)acrylate **5** and 2.5 equivalents of trimethylamine were added to the solution and kept at room temperature for 12 h. After a careful analysis of the crude compound, the corresponding bis(allylic-α-aminophosphonate) **6g** was isolated at very low yield (8%), together with an inseparable mixture of compounds. Unfortunately, when the one-pot reaction was performed in toluene, acetonitrile, EtOH, or even in the absence of solvent (both steps), the bis(allylic-α-aminophosphonate) **6** was observed in a very low yield.

As far as we know, this process represents the first example of the synthesis of bis(allylic-α-aminophosphonic ester) derivatives using the nucleophilic substitution reaction of bis(α-aminophosphonates) and ethyl (2-bromomethyl)acrylate under mild reaction conditions. Moreover, this bidentate bis(allylic-α-aminophosphonates) **6** could have a large range of applications. They can be used as monomers for the creation of new polymers containing the α-aminophosphonate moiety, starting materials for the preparation of higher-membered heterocycles, as well as ligands for the complexation of metals.

## 3. Materials and Methods

### 3.1. Chemistry

All starting reagents were obtained from commercial sources and used as received without further purification, or recrystallized or distilled as necessary. All solvents used in reactions were freshly distilled and dried over molecular sieves 4 Å before use. Solvents for extraction and chromatography were technical grade. All reactions were performed under an atmosphere of dry nitrogen. The reaction progress was monitored using ^31^P NMR or analytical thin layer chromatography (TLC) performed on precoated Merck silica gel 60 F_254_ TLC aluminium plates, and spot visualized using UV light or permanganate stain. Melting points were taken on a Büchi Melting Point B-540 apparatus and were uncorrected. ^1^H, ^13^C, ^19^F and ^31^P NMR spectra were recorded using a Bruker 300 MHz or a Bruker Avance 400 MHz (Bruker BioSpin GmbH, Rheinstetten, Germany) spectrometer at 300 or 400 MHz for ^1^H, 75 or 101 MHz for ^13^C, 376 MHz for ^19^F, and 121 or 161 MHz for ^31^P, respectively, in CDCl_3_ at room temperature. Chemical shifts (*δ*) are reported in parts per million (ppm) relative to residual CHCl_3_ (*δ* = 7.28 ppm for ^1^H and *δ* = 77.00 ppm for ^13^C NMR*)* and use phosphoric acid (50%) or CFCl_3_ as external reference (*δ* = 0.0 ppm) for ^31^P and ^19^F NMR spectra. All coupling constants (*J*) values are reported in Hz. Data for 1H NMR spectra are reported as follows: chemical shift, multiplicity, coupling constant, integration. Multiplicity abbreviations are reported as s = singlet, d = doublet, t = triplet, q = quartet, m = multiplet, dd = double doublet, bs = broad singlet. ^13^C NMR spectra were recorded in a broadband decoupled mode from hydrogen nuclei. Distortionless enhanced polarization transfer (DEPT) supported peak assignments for ^13^C NMR. High-resolution mass spectrum (HRMS) was acquired using a positive-ion electrospray ionization (ESI) method on an Agilent 6530 Accurate-Mass QTOF LC/MS (Santa Clara, CA, USA). The sample was analyzed using the time-of-flight Q-TOF method. Data are reported in the form *m/z* (intensity relative to base = 100). Chromatographic purification was performed as flash chromatography using commercial grades of silica gel finer than 230 mesh with pressure. Compounds **4a** [35,36], **4c** [35], **4e** [35] and (2-bromomethyl)acrylate **5** [37] were prepared according to procedures in the literature and characterized using NMR spectra.

#### 3.1.1. General Procedure and Spectral Data for the Multicomponent Kabachnik–Fields Reaction for the Preparation of Bis(α-aminophosphonates) **4**

Bisamine **1** (5 mmol, 1 eq), diethylphosphite (10 mmol, 2 eq) **2** and aldehyde **3** (10 mmol, 2 eq) were dissolved simultaneously in the appropriate solvent (50 mL). The mixture was refluxed under nitrogen atmosphere in ethanol for 20 h (method A) or for 5 to 6 h in toluene (method B). The reaction was monitored using TLC and ^31^P NMR. The solvent was removed from the mixture under vacuum and the residue was purified using flash column chromatography (SiO_2_, AcOEt) or recrystallized from diethyl ether (for solid compounds) to afford bis(α-aminophosphonates) **4**.

Tetraethyl [(ethane-1,2-diylbis(azanediyl))bis(phenylmethylene)]bis(phosphonate) (**4a**), (1.41 g, 55%) was obtained as a white solid following the general procedure (method A). The title compound **4a** (1.54 g, 60%) was obtained as white solid as described in the general procedure (method B). Data for **4a**: mp 97–98 °C. ^1^H NMR (400 MHz, CDCl_3_) *δ* 7.36–7.19 (m, 8H, Ar-CH), 4.09–3.69 (m, 10H, H_2_C-O and P-CH), 2.84–2.53 (m, 6H, CH_2_ and NH), 1.20 (t, 6H, ^3^*J*_HH_ = 7.0 Hz, CH_3_), 1.06 (t, 6H, ^3^*J*_HH_ = 7.1 Hz, CH_3_) ppm. ^31^P NMR (162 MHz, CDCl_3_) *δ* 23.3 ppm.

Tetraethyl [(ethane-1,2-diylbis(azanediyl))bis(p-tolylmethylene)]bis(phosphonate) (**4b**), (1.92 g, 71%) was obtained as a viscous oil following the general procedure (method A). The title compound **4b** (1.54 g, 57%) was obtained as viscous oil as described in the general procedure (method B). Data for **4b**: ^1^H NMR (400 MHz, CDCl_3_) *δ* 7.26–7.22 (m, 4H, Ar-CH), 7.14–7.09 (m, 4H, Ar-CH), 4.13–3.78 (m, 10H, H_2_C-O and P-CH), 2.63–2.46 (m, 4H, CH_2_), 2.32 (s, 6H, CH_3_), 2.17 (bs, 2H, NH), 1.29–1.12 (m, 12H, CH_3_) ppm. ^13^C {^1^H} NMR (101 MHz, CDCl_3_) *δ* 137.5 (Ar-C), 132.8 (Ar-C), 129.1 (Ar-CH), 128.4 (Ar-CH), 62.9 (P-OCH_2_), 60.4 (d, ^1^*J*_PC_ = 154.7 Hz, P-CH), 47.0 (CH_2_), 37.4 (CH_2_), 21.2 (CH_3_), 16.3 (CH_3_) ppm. ^31^P NMR (162 MHz, CDCl_3_) *δ* 23.7 ppm.

Tetraethyl [(propane-1,3-diylbis(azanediyl))bis(phenylmethylene]bis(phosphonate) (**4c**), (1.82 g, 69%) was isolated as a viscous clear oil using the general procedure (method A). The title compound **4c** (1.95 g, 74%) was produced as a viscous clear oil as described in the general procedure (method B). Data for **4c**: ^1^H NMR (400 MHz, CDCl_3_) *δ* 7.42–7.13 (m, 8H, Ar-CH), 4.12–3.62 (m, 10H, H_2_C-O and P-CH), 2.63–2.33 (m, 4H, CH_2_), 2.20 (bs, 2H, NH), 1.69–1.46 (m, 2H, CH_2_), 1.33–0.95 (m, 12H, CH_3_) ppm. ^31^P NMR (162 MHz, CDCl_3_) *δ* 23.4 ppm.

Tetraethyl [(propane-1,3-diylbis(azanediyl))bis(p-tolylmethylene]bis(phosphonate) (**4d**), (2.41 g, 87%) was isolated as a viscous oil using the general procedure (method A). The title compound **4d** (2.19 g, 79%) was produced as a viscous oil as described in the general procedure (method B). Data for **4d**: ^1^H NMR (400 MHz, CDCl_3_) *δ* 7.18 (d, ^3^*J*_HH_ = 7.2 Hz, 4H, Ar-CH), 7.06 (d, ^3^*J*_HH_ = 7.6 Hz, 4H, Ar-CH), 4.02–3.69 (m, 10H, H_2_C-O and P-CH), 2.55–2.37 (m, 4H, CH_2_), 2.26 (s, 6H, CH_3_), 2.09 (bs, 2H, NH), 1.57–1.45 (m, 2H, CH_2_), 1.18 (t, 6H, ^3^*J*_HH_ = 7.1 Hz, CH_3_), 1.06 (t, 6H, ^3^*J*_HH_ = 7.1 Hz, CH_3_) ppm. ^13^C {^1^H} NMR (101 MHz, CDCl_3_) *δ* 137.1 (Ar-C), 131.7 (Ar-C), 128.6 (Ar-CH), 128.0 (Ar-CH), 62.3 (P-OCH_2_), 59.9 (d, ^1^*J*_PC_ = 153.1 Hz, P-CH), 45.5 (d, ^3^*J*_PC_ = 15.2 Hz, CH_2_), 28.5 (CH_2_), 20.6 (CH_3_), 15.8 (d, ^3^*J*_PC_ = 17.2 Hz, CH_3_) ppm. ^31^P NMR (162 MHz, CDCl_3_) *δ* 23.6 ppm.

Tetraethyl [(propane-1,3-diylbis(azanediyl))bis((4-methoxyphenyl)methylene)]bis(phosphonate) (**4e**), (1.93 g, 66%) was isolated as a viscous clear oil as described in the general procedure (method A). The title compound **4e** (1.85 g, 63%) was obtained as a viscous clear oil as described in the general procedure (method B). Data for **4e**: ^1^H NMR (400 MHz, CDCl_3_) *δ* 7.25 (d, ^3^*J*_HH_ = 7.9 Hz, 4H, Ar-CH), 6.83 (d, ^3^*J*_HH_ = 8.5 Hz, 4H, Ar-CH), 4.35 (bs, 2H, NH), 4.03–3.88 (m, 10H, H_2_C-O and P-CH), 3.75 (s, 6H, CH_3_), 2.54–2.39 (m, 4H, CH_2_), 1.60–1.52 (m, 2H, CH_2_), 1.22 (t, 6H, ^3^*J*_HH_ = 7.0 Hz, CH_3_), 1.09 (t, 6H, ^3^*J*_HH_ = 7.0 Hz, CH_3_) ppm ^31^P NMR (162 MHz, CDCl_3_) *δ* 23.7 ppm.

Tetraethyl [(propane-1,3-diylbis(azanediyl))bis((4-chlorophenyl)methylene)]bis(phosphonate) (**4f**), (2.02 g, 68%) was isolated as a viscous oil as described in the general procedure (method A). The title compound **4f** (2.11 g, 71%) was obtained as viscous oil as described in the general procedure (method B). Data for **4f**: ^1^H NMR (400 MHz, CDCl_3_) *δ* 7.22–7.07 (m, 8H, Ar-CH), 3.93–3.64 (m, 10H, H_2_C-O and P-CH), 2.45–2.24 (m, 4H, CH_2_), 1.76 (bs, 2H, NH), 1.46–1.37 (m, 2H, CH_2_), 1.09–0.95 (m, 12H, CH_3_) ppm. ^13^C {^1^H} NMR (101 MHz, CDCl_3_) *δ* 134.3 (Ar-C), 132.8 (Ar-C), 129.2 (d, ^3^*J*_PC_ = 6.0 Hz,Ar-CH), 127.9 (Ar-CH), 62.3 (P-OCH_2_), 60.0 (d, ^1^*J*_PC_ = 152.8 Hz, P-C), 45.4 (d, ^3^*J*_PC_ = 16.6 Hz,CH_2_), 29.3 (CH_2_), 15.7 (CH_3_) ppm. ^31^P NMR (162 MHz, CDCl_3_) *δ* 22.6 ppm.

Tetraethyl [(propane-1,3-diylbis(azanediyl))bis((4-fluorophenyl)methylene)]bis(phosphonate) (**4g**), (1.69 g, 60%) was obtained as a viscous oil following the general procedure (method A). The title compound **4g** (1.63 g, 58%) was obtained as a viscous oil using the general procedure (method B). Data for **4g**: ^1^H NMR (400 MHz, CDCl_3_) *δ* 7.28–6.94 (m, 4H, Ar-CH), 6.71–6.50 (m, 4H, Ar-CH), 3.93–3.44 (m, 10H, H_2_C-O and P-CH), 2.33–2.05 (m, 4H, CH_2_), 1.71–1.63 (m, 2H, NH), 1.37–1.23 (m, 2H, CH_2_), 1.03–0.70 (m, 12H, CH_3_) ppm. ^13^C {^1^H} NMR (101 MHz, CDCl_3_) *δ* 161.5 (d, ^1^*J*_CF_ = 245.8 Hz, C-F), 131.3 (Ar-C), 129.2 (Ar-CH), 114.3 (d, ^3^*J*_PC_ = 21.6 Hz, Ar-CH), 61.8 (P-OCH_2_), 59.6 (d, ^1^*J*_PC_ = 153.3 Hz, P-CH), 45.1 (d, ^3^*J*_PC_ = 17.3 Hz, CH_2_), 29.0 (CH_2_), 15.4 (d, ^3^*J*_PC_ = 15.8 Hz, CH_3_) ppm. ^31^P NMR (162 MHz, CDCl_3_) *δ* 22.9 ppm. ^19^F NMR (376 MHz, CDCl_3_) *δ* –115.0 ppm.

Tetraethyl [(propane-1,3-diylbis(azanediyl))bis(ethane-1,1-diyl)]bis(phosphonate) (**4h**), (1.59 g, 79%) was isolated as a viscous oil as described in the general procedure (method A). The title compound **4h** (1.73 g, 86%) was obtained as a viscous oil as described in the general procedure (method B). Data for **4h**: ^1^H NMR (400 MHz, CDCl_3_) *δ* 4.19–4.08 (m, 10H, H_2_C-O and P-CH), 3.00–2.91 (m, 2H, CH_2_), 2.84–2.67 (m, 4H, CH_2_), 1.48 (bs, 2H, NH), 1.34–1.25 (m, 18H, CH_3_). ^13^C {^1^H} NMR (101 MHz, CDCl_3_) 62.0 (P-OCH_2_), 50.5 (d, ^1^*J*_PC_ = 153.9 Hz, P-CH), 46.1 (d, ^3^*J*_PC_ = 11.0 Hz, CH_2_), 30.6 (CH_2_), 16.5 (d, ^3^*J*_PC_ = 5.53 Hz,CH_3_), 15.2 (CH_3_) ppm. ^31^P NMR (162 MHz, CDCl_3_) *δ* 28.6 ppm.

#### 3.1.2. General Procedure and Spectral Data for the Nucleophilic Substitution of Bis(α-aminophosphonates) **4** with Ethyl (2-Bromomethyl)acrylate **5** for the Synthesis of Bis(allylic-α-aminophosphonates) **6**

To a stirred solution of bis(α-aminophosphonates) **4** (0.7 mmol, 1 eq) in dry THF (10–15 mL), triethylamine (1.75 mmol, 2.5 eq) was slowly added. After 15 min, ethyl 2-bromomethylacrylate (**5**) (1.47 mmol, 2.1 eq) was added dropwise at 0 °C. The mixture was allowed to stir at room temperature under an inert atmosphere. After 12 h, the solvent was removed, and the residue was neutralized using a 1 M aqueous solution of HCl and then extracted using diethyl ether (3 × 30 mL). The crude product was purified using flash column chromatography (SiO_2_, AcOEt/hexane 50:50) to obtain compounds **6**.

Diethyl 2,2′-[(ethane-1,2-diylbis(((diethoxyphosphoryl)(phenyl)methyl)azanediyl))bis(methylene)]diacrylate (**6a**), (0.36 g, 69%) was isolated as a white solid as described in the general procedure. Data for **6a**: mp 150–151 °C. ^1^H NMR (300 MHz, CDCl_3_) *δ* 7.56–7.33 (m, 10H, Ar-CH), 6.24 (s, 2H, H_2_C=), 5.86 (s, 2H, H_2_C=), 4.21–4.08 (m, m, 10H, H_2_CO-P and HC-P), 3.92–3.86 (m, 2H, H_2_CO-C), 3.76–3.66 (m, 2H, H_2_CO-C and H_2_C-N), 3.35–3.30 (m, 2H, H_2_C-N), 3.21–3.17 (m, 2H, H_2_C-N), 2.50–2.37 (m, 2H, CH_2_), 1.28–1.22 (m, 12H, CH_3_), 0.98 (t, ^3^*J*_HH_ = 7.2 Hz, 6H, CH_3_) ppm. ^13^C {^1^H} NMR (101 MHz, CDCl_3_) *δ* 166.7 (C=O), 138.6 (C=CH_2_), 132.4 (d, ^2^*J*_PC_ = 5.8 Hz Ar-C), 130.8 (d, ^3^*J*_PC_ = 8.7 Hz Ar-CH), 128.1 (Ar-CH), 127.9 (Ar-CH), 125.9 (H_2_C=), 62.4 (P-OCH_2_), 61.4 (d, ^1^*J*_PC_ = 162.8 Hz, P-CH), 60.4 (C-OCH_2_), 52.7 (d, ^3^*J*_PC_ = 10.3 Hz, N-CH_2_), 50.5 (d, ^3^*J*_PC_ = 5.8 Hz, N-CH_2_), 31.6 (CH_2_), 29.0 (CH_2_), 16.4 (d, ^3^*J*_PC_ = 5.8 Hz, CH_3_), 14.1 (CH_3_) ppm. ^31^P NMR (162 MHz, CDCl_3_) *δ* 23.1 ppm. ESI-HRMS (CI) *m/z* calcd for C_36_H_55_N_2_O_10_P_2_ ([M + H]^+^) 737.3332, found 737.3334.

Diethyl 2,2′-[(ethane-1,2-diylbis(((diethoxyphosphoryl)(p-tolyl)methyl)azanediyl))bis(methylene)]diacrylate (**6b**), (0.46 g, 85%) was isolated as a viscous oil as described in the general procedure. Data for **6b**: ^1^H NMR (400 MHz, CDCl_3_) *δ* 7.32 (d, ^3^*J*_HH_ = 7.7 Hz, 4H, Ar-CH), 7.13 (d, ^3^*J*_HH_ = 8.0 Hz, 4H, Ar-CH), 6.28 (s, 2H, H_2_C=), 5.93 (s, 2H, H_2_C=), 4.22–4.01 (m, 10H, H_2_CO-P and HC-P), 3.97–3.87 (m, 2H, H_2_CO-C), 3.80–3.66 (m, 4H, H_2_CO-C and H_2_C-N), 3.27–3.24 (m, 2H, H_2_C-N), 3.19–3.14 (m, 2H, CH_2_), 2.55–2.49 (m, 2H, CH_2_), 2.35 (s, 6H, CH_3_), 1.33 (t, 6H, ^3^*J*_HH_ = 7.2 Hz, CH_3_), 1.26 (t, 6H, ^3^*J*_HH_ = 7.2 Hz, CH_3_), 1.05 (t, ^3^*J*_HH_ = 7.1 Hz, 6H, CH_3_) ppm. ^13^C {^1^H} NMR (101 MHz, CDCl_3_) *δ* 166.9 (C=O), 138.5 (C=CH_2_), 137.5 (Ar-C), 130.5 (Ar-CH), 130.4 (Ar-CH), 128.9 (Ar-C), 126.0 (H_2_C=), 62.5 (P-OCH_2_), 61.7 (d, ^1^*J*_PC_ = 151.6 Hz, P-CH), 60.5 (C-OCH_2_), 52.5 (N-CH_2_), 50.6 (N-CH_2_), 21.1 (CH_3_), 16.6 (d, ^3^*J*_PC_ = 5.6 Hz, CH_3_), 16.2 (d, ^3^*J*_PC_ = 5.8 Hz, CH_3_), 14.2 (CH_3_) ppm. ^31^P NMR (162 MHz, CDCl_3_) *δ* 23.7 ppm. ESI-HRMS (CI) *m/z* calcd for C_38_H_59_N_2_O_10_P_2_ ([M + H]^+^) 765.3645, found 765.3638.

Diethyl 2,2′-[(propane-1,3-diylbis(((diethoxyphosphoryl)(phenyl)methyl)azanediyl))bis(methylene)]diacrylate (**6c**), (0.39 g, 74%) obtained as a viscous clear oil as described in the general procedure. Data for **6c**: ^1^H NMR (300 MHz, CDCl_3_) *δ* 7.39–7.18 (m, 10H, Ar-CH), 6.11 (s, 2H, H_2_C=), 5.68 (s, 2H, H_2_C=), 4.20–3.67 (m, 14H, H_2_CO-P, H_2_CO-C, HC-P), 3.16 (s, 4H, H_2_C-N), 2.50–2.32 (m, 4H, H_2_C-N), 1.61–1.52 (m, 2H, CH_2_), 1.27–1.14 (m, 12H, CH_3_), 1.06 (t, ^3^*J*_HH_ = 7.3 Hz, 6H, CH_3_) ppm. ^13^C {^1^H} NMR (75 MHz, CDCl_3_) *δ* 165.8 (C=O), 137.3 (C=CH_2_), 135.0 (Ar-C), 127.4 (Ar-CH), 127.3 (Ar-CH), 126.7 (Ar-CH), 124.6 (C=CH_2_), 61.8 (P-OCH_2_), 60.1 (d, ^1^*J*_PC_ = 153.1 Hz, CH-P), 59.5 (C-OCH_2_), 53.5 (N-CH_2_), 51.1 (CH_2_), 45.0 (CH_2_), 28.6 (CH_2_), 15.2 (CH_3_), 13.1 (CH_3_) ppm. ^31^P NMR (121 MHz, CDCl_3_) *δ* 23.6 ppm. ESI-HRMS (CI) *m/z* calcd for C_37_H_56_N_2_O_10_P_2_ ([M + H]^+^) 751.3488, found 751.3494.

Diethyl 2,2′-[(propane-1,3-diylbis(((diethoxyphosphoryl)(p-tolyl)methyl)azanediyl))bis(methylene)]diacrylate (**6d**), (0.39 g, 71%) was isolated as a viscous clear oil as described in the general procedure. Data for **6d**: ^1^H NMR (300 MHz, CDCl_3_) *δ* 7.37–7.00 (m, 8H, Ar-CH), 6.21 (s, 2H, H_2_C=), 5.89 (s, 2H, H_2_C=), 4.19–3.56 (m, 12H, H_2_CO-P, HC-P and H_2_CO-C), 3.72–3.56 (m, 4H, H_2_CO-C and H_2_C-N), 3.20–3.03 (m, 2H, H_2_C-N), 2.98–2.87 (m, 2H, CH_2_), 2.26 (s, 6H, CH_3_), 1.74–1.51 (m, 2H, CH_2_), 1.29–1.14 (m, 12H, CH_3_), 0.98–0.92 (m, 6H, CH_3_) ppm. ^13^C {^1^H} NMR (101 MHz, CDCl_3_) *δ* 166.7 (C=O), 138.3 (C=CH_2_), 137.3 (Ar-C), 130.3 (d, ^2^*J*_PC_ = 8.6 Hz, Ar-C), 129.4 (d, ^3^*J*_PC_ = 6.0 Hz, Ar-CH), 128.6 (Ar-CH), 125.7 (C=CH_2_), 62.0 (d, ^2^*J*_PC_ = 7.1 Hz, P-OCH_2_), 61.9 (d, ^2^*J*_PC_ = 7.0 Hz, P-OCH_2_), 61.2 (d, ^1^*J*_PC_ = 138.7 Hz, P-CH), 60.2 (C-OCH_2_), 51.7 (N-CH_2_), 49.6 (N-CH_2_), 26.8 (CH_2_), 20.9 (CH_3_), 16.3 (d, ^3^*J*_PC_ = 5.9 Hz, CH_3_), 16.0 (d, ^3^*J*_PC_ = 5.6 Hz, CH_3_), 14.0 (CH_3_) ppm. ^31^P NMR (121 MHz, CDCl_3_) *δ* 23.7 ppm. ESI-HRMS (CI) *m/z* calcd for C_39_H_61_N_2_O_10_P_2_ ([M + H]^+^) 779.3801, found 779.3810.

Diethyl 2,2′-[(propane-1,3-diylbis(((diethoxyphosphoryl)(4-methoxyphenyl)methyl)azanediyl))bis(methylene)]diacrylate (**6e**), (0.42 g, 74%) was isolated as a viscous clear oil as described in the general procedure. Data for **6e**: ^1^H NMR (400 MHz, CDCl_3_) *δ* 7.39–7.36 (m, 4H, Ar-CH), 6.88–6.85 (m, 4H, Ar-CH), 6.29 (s, 2H, H_2_C=), 5.95 (s, 2H, H_2_C=), 4.20–3.86 (m, 12H, H_2_CO-P, HC-P and H_2_CO-C), 3.81 (s, 6H, OCH_3_), 3.75–3.67 (m, 4H, H_2_CO-C and H_2_C-N), 3.20–3.13 (m, 2H, H_2_C-N), 3.04–2.94 (m, 2H, CH_2_), 2.42–2.27 (m, 2H, CH_2_), 1.71–1.62 (m, 2H, CH_2_), 1.31–1.25 (m, 12H, CH_3_), 0.96 (t, ^3^*J*_HH_ = 7.1, 3H, CH_3_), 0.94 (t, ^3^*J*_HH_ = 7.1, 3H, CH_3_) ppm. ^13^C {^1^H} NMR (101 MHz, CDCl_3_) *δ* 166.9 (C=O), 159.2 (Ar-C), 138.5 (C=CH_2_), 131.9 (Ar-C), 125.9 (=CH_2_), 124.7 (d, ^3^*J*_PC_ = 5.9 Hz, Ar-CH), 124.5 (d, ^3^*J*_PC_ = 5.9 Hz, Ar-CH), 113.5 (Ar-CH), 62.2 (d, ^2^*J*_PC_ = 7.3Hz, P-OCH_2_), 62.1 (d, ^2^*J*_PC_ = 7.3 Hz, P-OCH_2_), 61.1 (d, ^1^*J*_PC_ = 161.2 Hz, P-CH), 60.5 (C-OCH_2_), 55.1 (N-CH_2_), 51.7 (N-CH_2_), 49.8 (CH_2_), 27.0 (CH_3_), 16.5 (d, ^3^*J*_PC_ = 5.8 Hz, CH_3_), 16.2 (d, ^3^*J*_PC_ = 5.8 Hz, CH_3_), 14.2 (CH_3_) ppm. ^31^P NMR (162 MHz, CDCl_3_) *δ* 23.7 ppm. ESI-HRMS (CI) *m/z* calcd for C_39_H_61_N_2_O_12_P_2_ ([M + H]^+^) 811.3700, found 811.3697.

Diethyl 2,2′-[(propane-1,3-diylbis(((4-chlorophenyl)(diethoxyphosphoryl)methyl)azanediyl))bis(methylene)]diacrylate (**6f**), (0.50 g, 87%) obtained as a viscous clear oil as described in the general procedure. Data for **6f**: ^1^H NMR (300 MHz, CDCl_3_) *δ* 7.39–7.11 (m, 8H, Ar-CH), 6.21 (s, 2H, H_2_C=), 5.79 (s, 2H, H_2_C=), 4.25–3.51 (m, 16H, H_2_CO-P, HC-P, H_2_CO-C, and H_2_C-N), 3.24–2.70 (m, 4H, H_2_C-N), 2.34–2.14 (m, 2H, CH_2_), 1.76–1.44 (m, 2H, CH_2_), 1.23–0.76 (m, 18H, CH_3_) ppm. ^13^C {^1^H} NMR (75 MHz, CDCl_3_) *δ* 166.6 (C=O), 138.4 (C=CH_2_), 133.8 (Ar-C), 131.7 (Ar-CH), 128.2 (Ar-CH), 125.9 (=CH_2_), 124.6 (Ar-C), 62.2 (P-OCH_2_), 60.8 (d, ^1^*J*_PC_ = 143.7 Hz, P-CH), 60.4 (C-OCH_2_), 51.9 (N-CH_2_), 49.7 (N-CH_2_), 26.9 (CH_2_), 16.3 (CH_3_), 14.0 (CH_3_) ppm. ^31^P NMR (121 MHz, CDCl_3_) *δ* 23.00 ppm. ESI-HRMS (CI) *m/z* calcd for C_37_H_55_Cl_2_N_2_O_10_P_2_ ([M + H]^+^) 819.2709, found 819.2714.

Diethyl 2,2′-[(propane-1,3-diylbis(((diethoxyphosphoryl)(4-fluorophenyl)methyl)azanediyl))bis(methylene)]diacrylate (**6g**), (0.41 g, 75%) was isolated as a viscous clear oil as described in the general procedure. Data for **6g**: ^1^H NMR (400 MHz, CDCl_3_) *δ* 7.45–7.41 (m, 4H, Ar-CH), 7.06–7.00 (m, 4H, Ar-CH), 6.28 (s, 2H, H_2_C=), 5.93 (s, 2H, H_2_C=), 4.22–4.07 (m, 10H, H_2_CO-P and HC-P), 3.97–3.87 (m, 2H, H_2_CO-C), 3.81–3.68 (m, 4H, H_2_CO-C and H_2_C-N), 3.23–3.18 (m, 2H, H_2_C-N), 3.05–2.98 (m, 2H, CH_2_), 2.35–2.29 (m, 2H, CH_2_), 1.68–1.60 (m, 2H, CH_2_), 1.33–1.26 (m, 12H, CH_3_), 1.06 (t, 6H, ^3^*J*_HH_ = 7.3 Hz, CH_3_) ppm. ^13^C {^1^H} NMR (101 MHz, CDCl_3_) *δ* 166.7 (C=O), 162.4 (d, ^1^*J*_CF_ = 245.9 Hz, C-F), 138.4 (C=CH_2_), 132.2 (Ar-CH), 128.9 (Ar-C), 126.0 (C=CH_2_), 115.2 (d, ^3^*J*_PC_ = 21.1 Hz, Ar-CH), 62.2 (d, ^2^*J*_PC_ = 20.9 Hz, P-OCH_2_), 61.0 (d, ^1^*J*_PC_ = 159.4 Hz, P-C), 60.5 (C-OCH_2_), 51.8 (N-CH_2_), 49.7 (N-CH_2_), 27.0 (CH_2_), 16.5 (CH_3_), 16.2 (CH_3_) ppm. ^31^P NMR (162 MHz, CDCl_3_) *δ* 23.4 ppm. ^19^F NMR (376 MHz, CDCl_3_) *δ* −114.2 ppm. ESI-HRMS (CI) *m/z* calcd for C_37_H_55_F_2_N_2_O_10_P_2_ ([M + H]^+^) 787.3300, found 787.3295.

Diethyl 2,2′-[(propane-1,3-diylbis((1-(diethoxyphosphoryl)ethyl)azanediyl))bis(methylene)]diacrylate (**6h**), (0.30 g, 69%) was isolated as a viscous clear oil as described in the general procedure. Data for **6h**: ^1^H NMR (400 MHz, CDCl_3_) *δ* 6.13 (s, 2H, H_2_C=), 5.77 (s, 2H, H_2_C=), 4.14–3.95 (m, 12H, H_2_CO-P, HC-P and H_2_CO-C), 3.50–3.26 (m, 4H, H_2_CO-C and H_2_C-N), 3.07–2.99 (m, 2H, H_2_C-N), 2.52–2.41 (m, 2H, CH_2_), 1.49–1.45 (m, 2H, CH_2_), 1.25–1.13 (m, 24H, CH_3_) ppm. ^13^C {^1^H} NMR (101 MHz, CDCl_3_) *δ* 166.7 (C=O), 139.0 (C=CH_2_), 125.3 (=CH_2_), 61.0 (d, ^2^*J*_PC_ = 7.2 Hz, P-OCH_2_), 60.3 (C-OCH_2_), 52.0 (N-CH_2_), 51.4 (d, ^1^*J*_PC_ = 141.6 Hz, P-CH), 49.2 (N-CH_2_), 28.1 (CH_2_), 16.4 (CH_3_), 14.0 (CH_3_), 11.2 (d, ^3^*J*_CP_ = 4.7 Hz, CH_3_), 10.7 (d, ^3^*J*_CP_ = 4.8 Hz, CH_3_) ppm. ^31^P NMR (162 MHz, CDCl_3_) *δ* 28. 5 ppm. ESI-HRMS (CI) *m/z* calcd for C_27_H_53_N_4_O_10_P_2_ ([M + H]^+^) 627.3175, found, 627.3175.

## 4. Conclusions

We reported a straightforward Kabacknik–Fields three-component protocol using ethane 1,2-diamine or propane 1,3-diamine for the synthesis of bis(α-aminophosphonates) **4**. These compounds have been used for an original preparation of new double allylic (α-aminophosphonates) **6** through the nucleophilic substitution reaction of bis(α-aminophosphonates) **4** with ethyl (2-bromomethyl)acrylate, under mild reaction conditions. As far as we know, this methodology constitutes the first example of the synthesis of bis(allylic-α-aminophosphonates). The 1,3-dipolar cycloaddition reactivity of the title *N*,*N*-[bis(allylic-α-aminomethylene phosphonates)] **6** used as dipolarophiles towards chloroximes and their biological activity, the preparation of macrocycles from compounds **6**, as well as their use as ligands for the complexation of metals are already ongoing in our laboratory.

## Figures and Tables

**Table 1:**
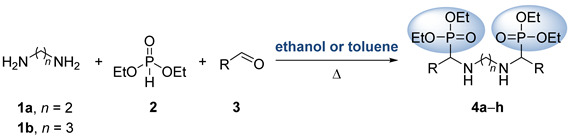
Synthesis of bis(α-aminophosphonates) **4a**–**h** using Kabachnik–Fields three-component condensation under catalyst-free conditions ^[a]^.

Entry	Compound	R	*n*	Yield (%) ^[b]^	
				Method A	Method B
1	**4a**	C_6_H_5_	2	55	60
2	**4b**	*p*-MeC_6_H_4_	2	71	57
3	**4c**	C_6_H_5_	3	69	74
4	**4d**	*p*-MeC_6_H_4_	3	87	79
5	**4e**	*p*-MeOC_6_H_4_	3	66	63
6	**4f**	*p*-ClC_6_H_4_	3	68	71
7	**4g**	*p*-FC_6_H_4_	3	60	58
8	**4h**	Me	3	79	86

^[a]^ 2 Equivalents of aldehyde **3** react with 2 equivalents of diethylphosphite **2** and 1 equivalent of diamine **1** refluxing in EtOH (method A) or refluxing in toluene (method B). ^[b]^ Isolated yields after purification using flash column chromatography.

**Table 2:**
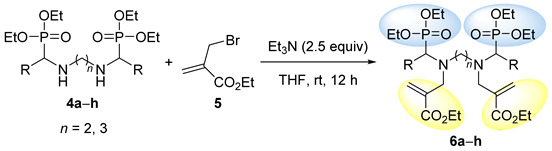
Synthesis of bis(allylic-α-aminophosphonates) **6a**–**h**.

Entry	Compound	R	*n*	Yield (%) ^[a]^
1	**6a**	C_6_H_5_	2	69
2	**6b**	*p*-MeC_6_H_4_	2	85
3	**6c**	C_6_H_5_	3	74
4	**6d**	*p*-MeC_6_H_4_	3	71
5	**6e**	*p*-MeOC_6_H_4_	3	74
6	**6f**	*p*-ClC_6_H_4_	3	87
7	**6g**	*p*-FC_6_H_4_	3	75
8	**6h**	Me	3	69

^[a]^ Isolated yields after purification using flash column chromatography.

## Data Availability

The data presented in this study are available in the Supplementary Materials file or on request from the corresponding author (^1^H, ^13^C, ^19^F, ^31^P, 2D-COSY, 2D-HSQC and 2D-HMBC NMR and HRMS spectra).

## Supplementary Materials

The following supporting information can be downloaded at: https://www.mdpi.com/article/10.3390/molecules28124678/s1, ^1^H, ^13^C, ^19^F, ^31^P and 2D-NMR copies of compounds **4** and **6**; 2D-NMR spectra of compounds **4g, 6g.**

## Sample Availability

Samples of the compounds are not available.
