# Supplementary material for: Synthesis and Characterization of a New Series of Bis(allylic-α-aminophosphonates) under Mild Reaction Conditions
Source: Molecules. 2023 Jun 9;28(12):4678. doi: 10.3390/molecules28124678 (PMC10302223; doi:10.3390/molecules28124678)

Supplementary Materials

Synthesis and characterization of a new series of bis-(allylic-α-aminopphosphonates) under mild reaction conditions

Ichrak Souii[1,3,*], Mohamed A. Sanhourry[2], Javier Vicario[3], Xabier Jiménez-Aberásturi[3], Mohamed L. Efrit[1], Hedi M'rabet[1] and Jesús M. de los Santos[3,*]

[1] Laboratory of Selective Organic & Heterocyclic Synthesis Biological Activity Evaluation (LR17ES01), Department of Chemistry, Faculty of Sciences, University of Tunis El Manar, 2092 Tunis-Tunisia
[2] Laboratory of Structural Organic Chemistry: Synthesis and Physicochemical Studies, Department of Chemistry. Faculty of Sciences of Tunis, University of Tunis El Manar 2092, El Manar I, Tunis, Tunisia
[3] Department of Organic Chemistry I. Faculty of Pharmacy and Lascaray Research Center, University of the Basque Country (UPV/EHU). Paseo de la Universidad 7, 01006 Vitoria, Spain
* Correspondence: ichrak.souii@fst.utm.tn (I.S.), ORCID: 0009-0006-2522-343X; jesus.delossantos@ehu.eus (J.M.S), ORCID: 0000-0003-1315-4263

Table of contents

NMR Spectral Charts — Page S2

^{1}H, ^{31}P, ^{19}F ^{13}C NMR spectra of of bis(α-aminophosphonates) **4** — Pages S2–S26

2D-NMR spectra of bis(α-aminophosphonate) **4g** — Pages S21–S23

^{1}H, ^{31}P, ^{19}F ^{13}C NMR spectra of of bis(allylic-α-aminophosphonates) **6** — Page S27–S54

2D-NMR spectra of bis(allylic-α-aminophosphonate) **6d** — Pages S49–S51

¹H NMR (400 MHz, $CDCl_3$) of compound **4a**.

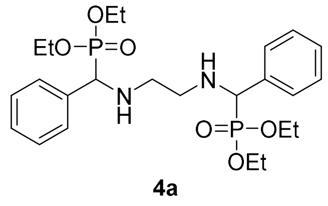

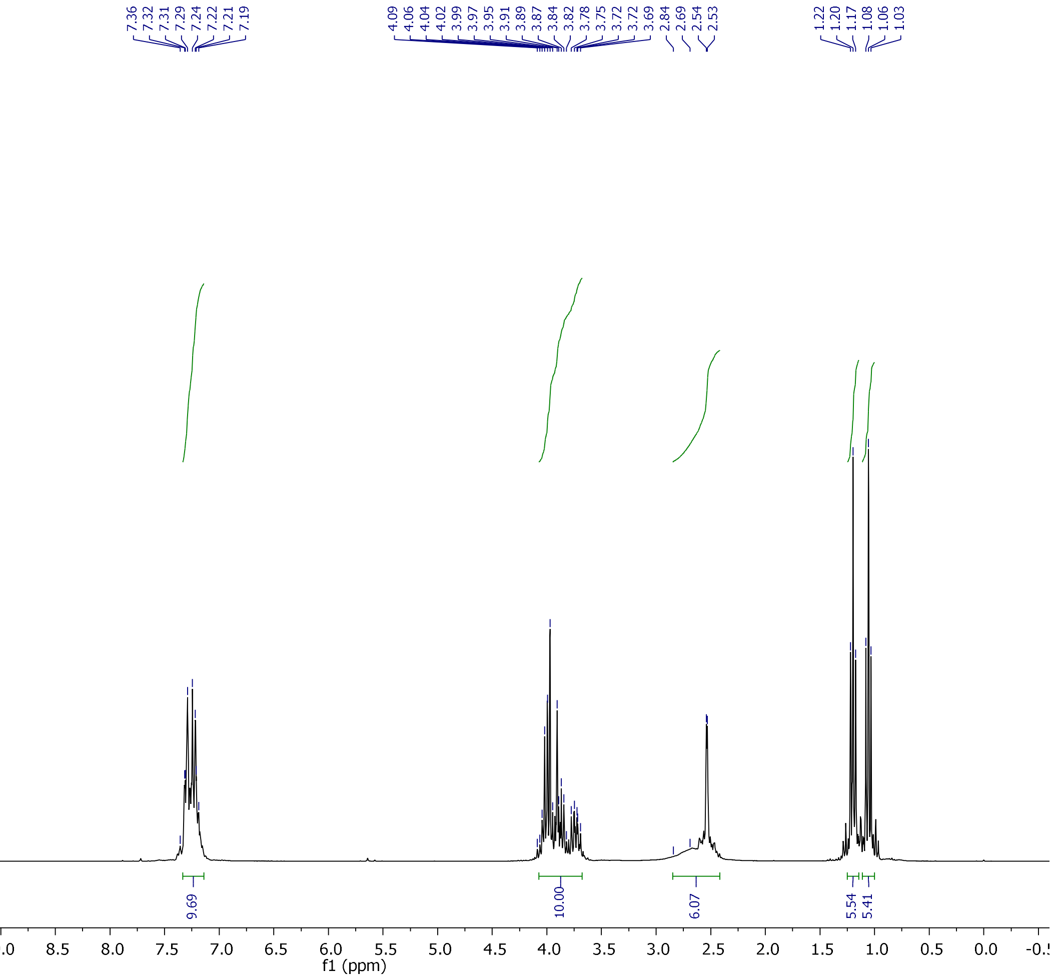

^{31}P NMR (162 MHz, $CDCl_3$) of compound **4a**.

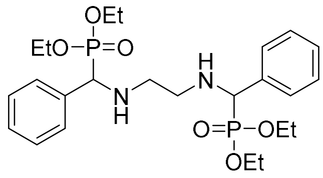

4a

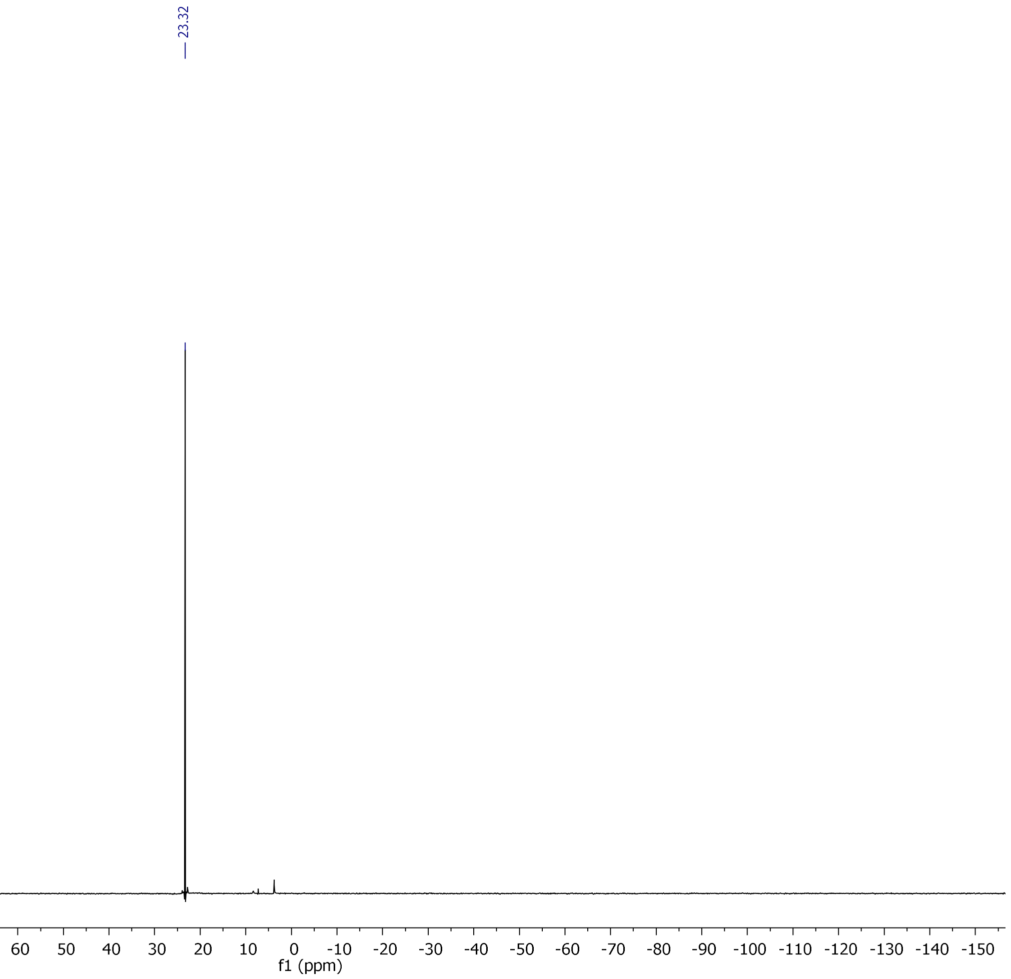

^{1}H NMR (400 MHz, $CDCl_3$) of compound **4b**.

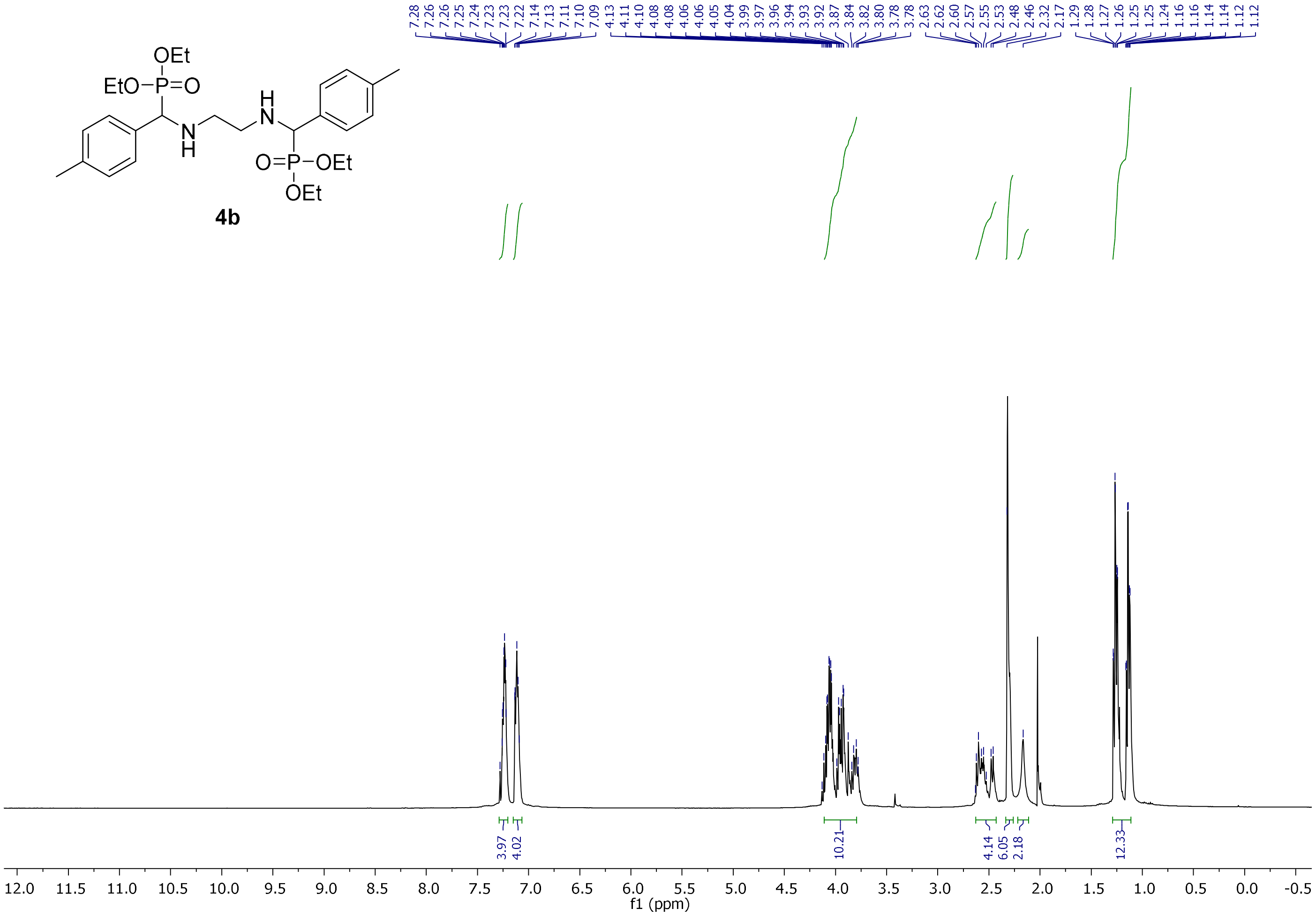

^{13}C {^{1}H} NMR (101 MHz, $CDCl_3$) of compound **4b**.

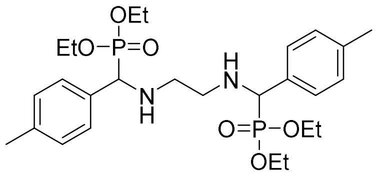

4b

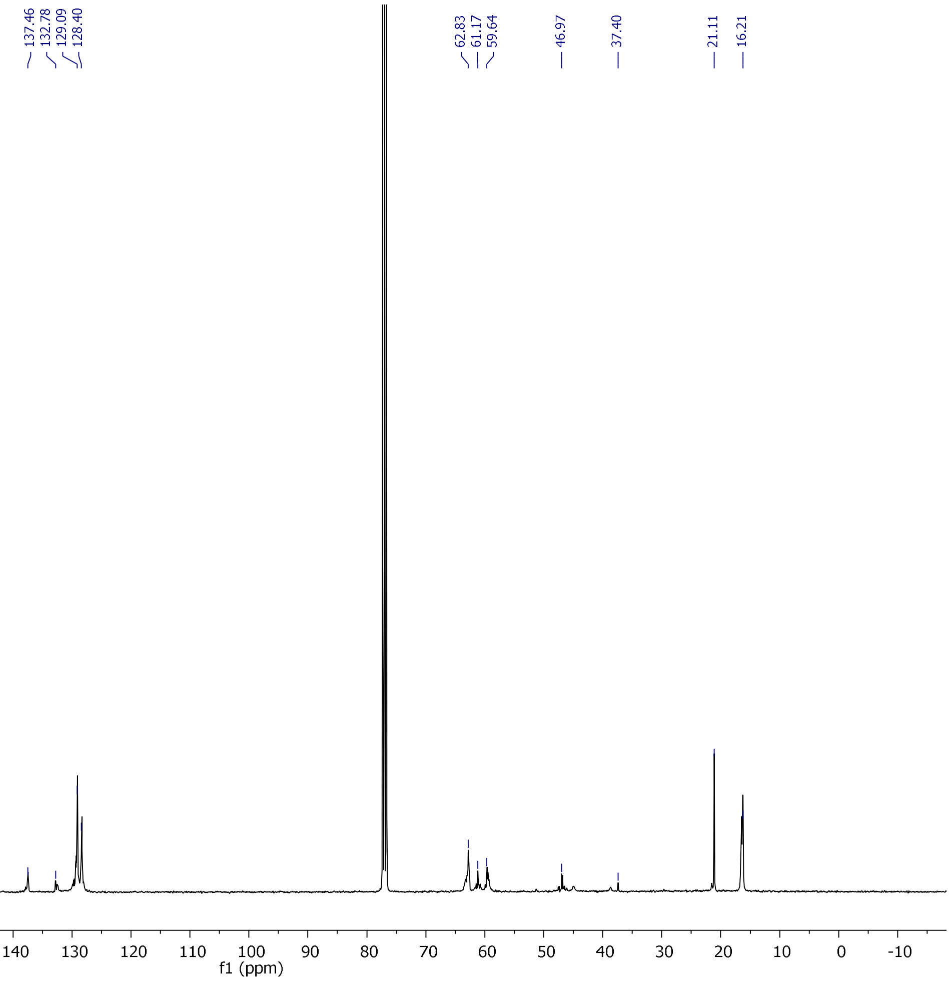

^{31}P NMR (162 MHz, $CDCl_3$) of compound **4b**.

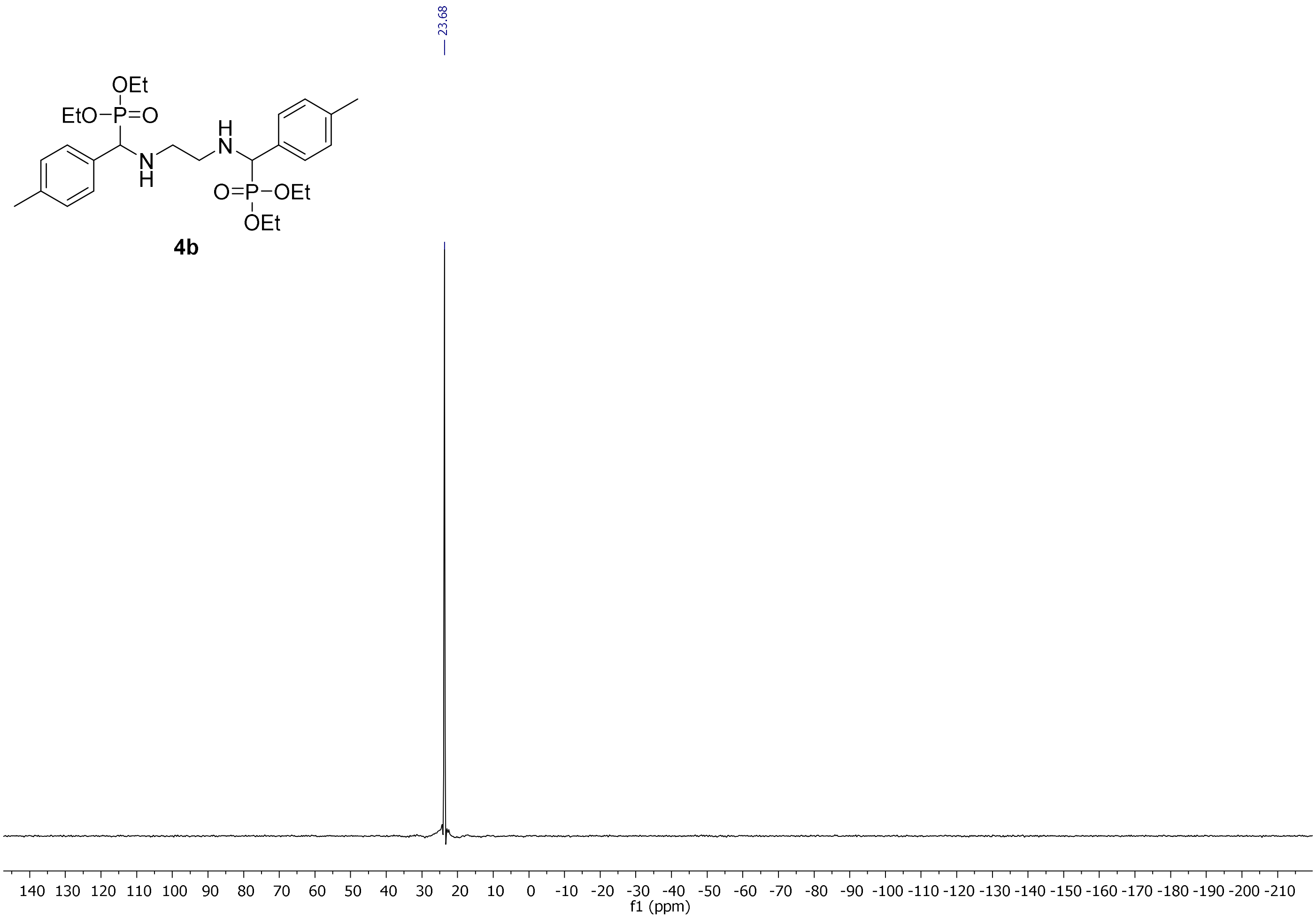

^{1}H NMR (400 MHz, $CDCl_3$) of compound **4c**.

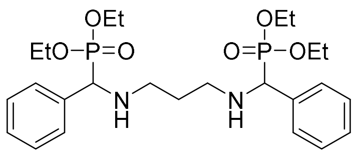

4c

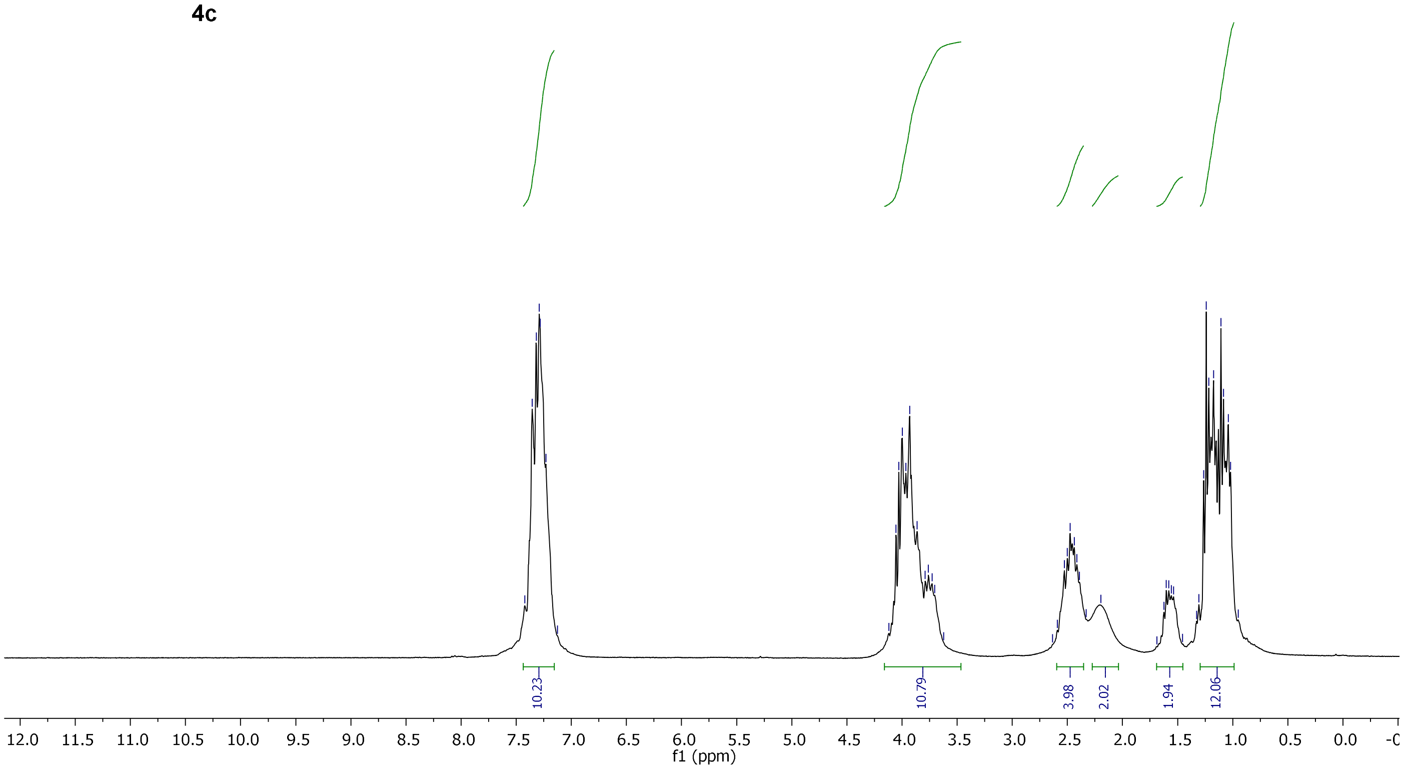

^{31}P NMR (162 MHz, $CDCl_3$) of compound **4c**.

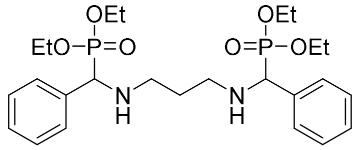

4c

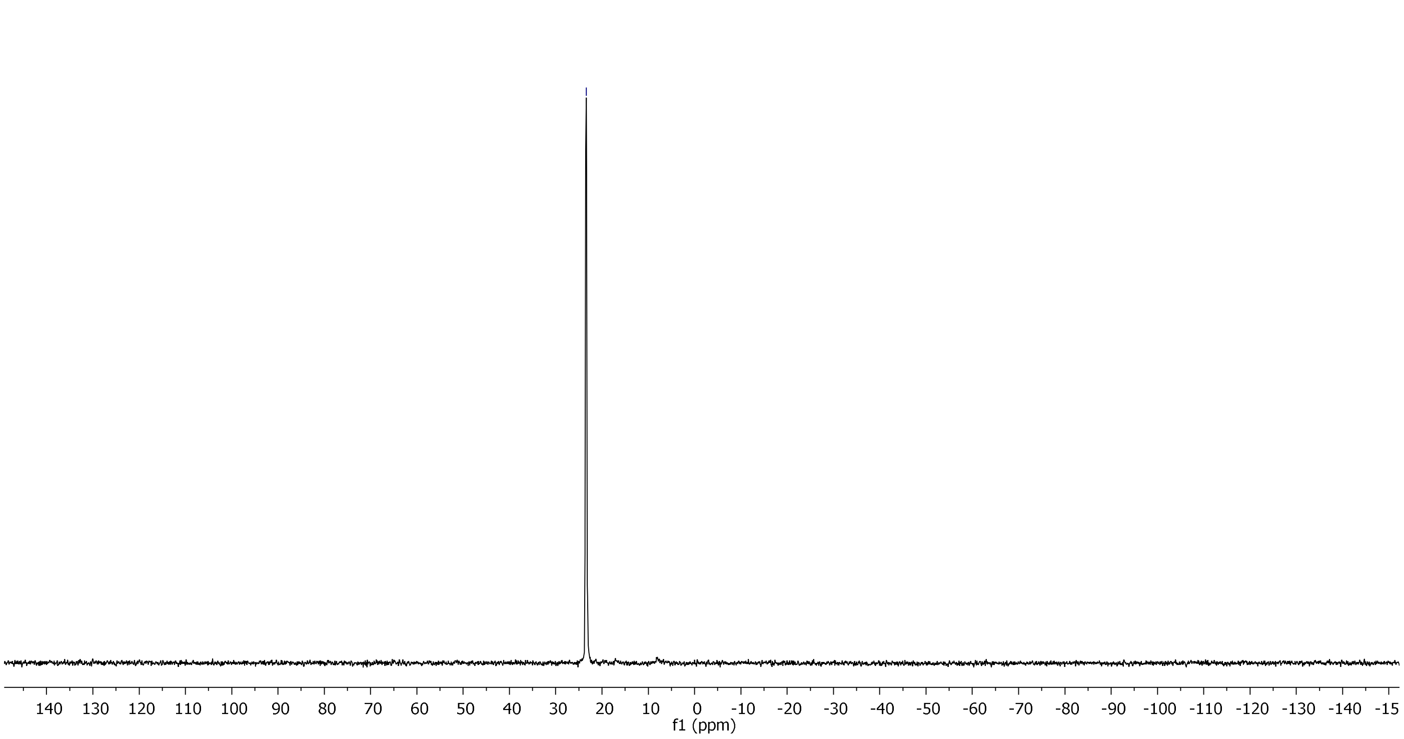

^{1}H NMR (400 MHz, $CDCl_3$) of compound **4d**.

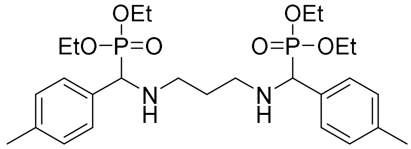

4d

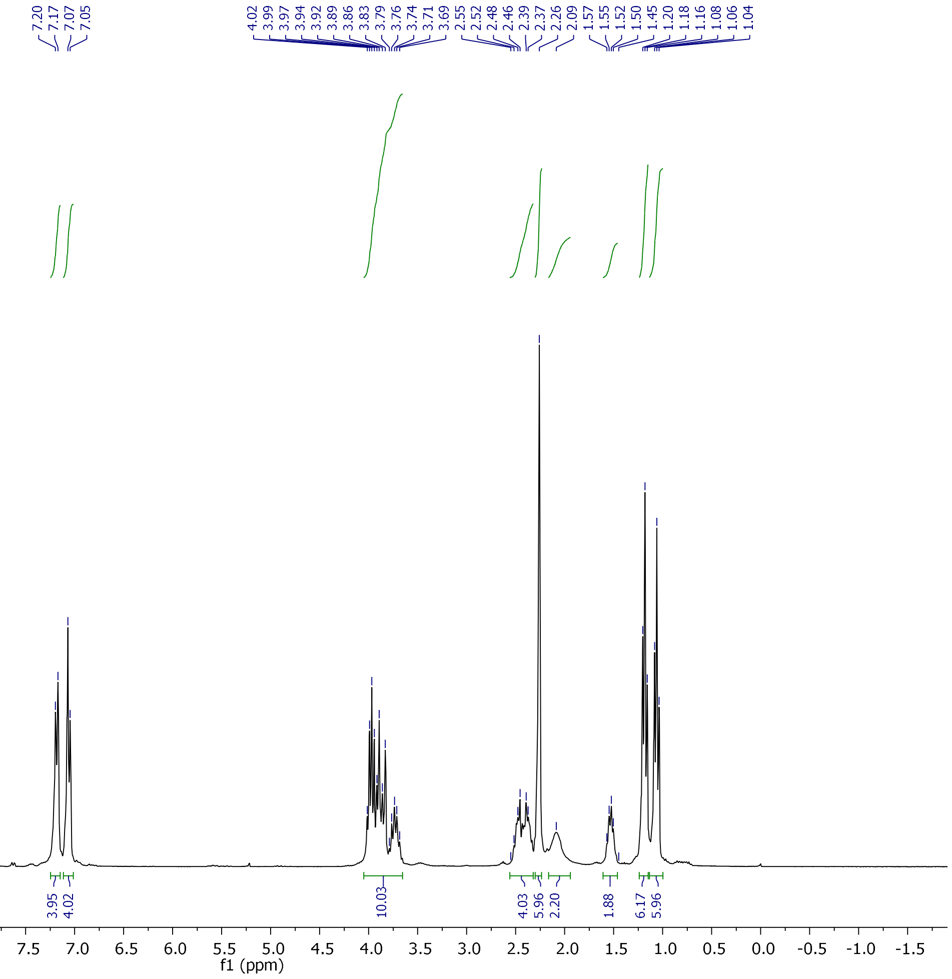

^{13}C {^{1}H} NMR (101 MHz, $CDCl_3$) of compound **4d**.

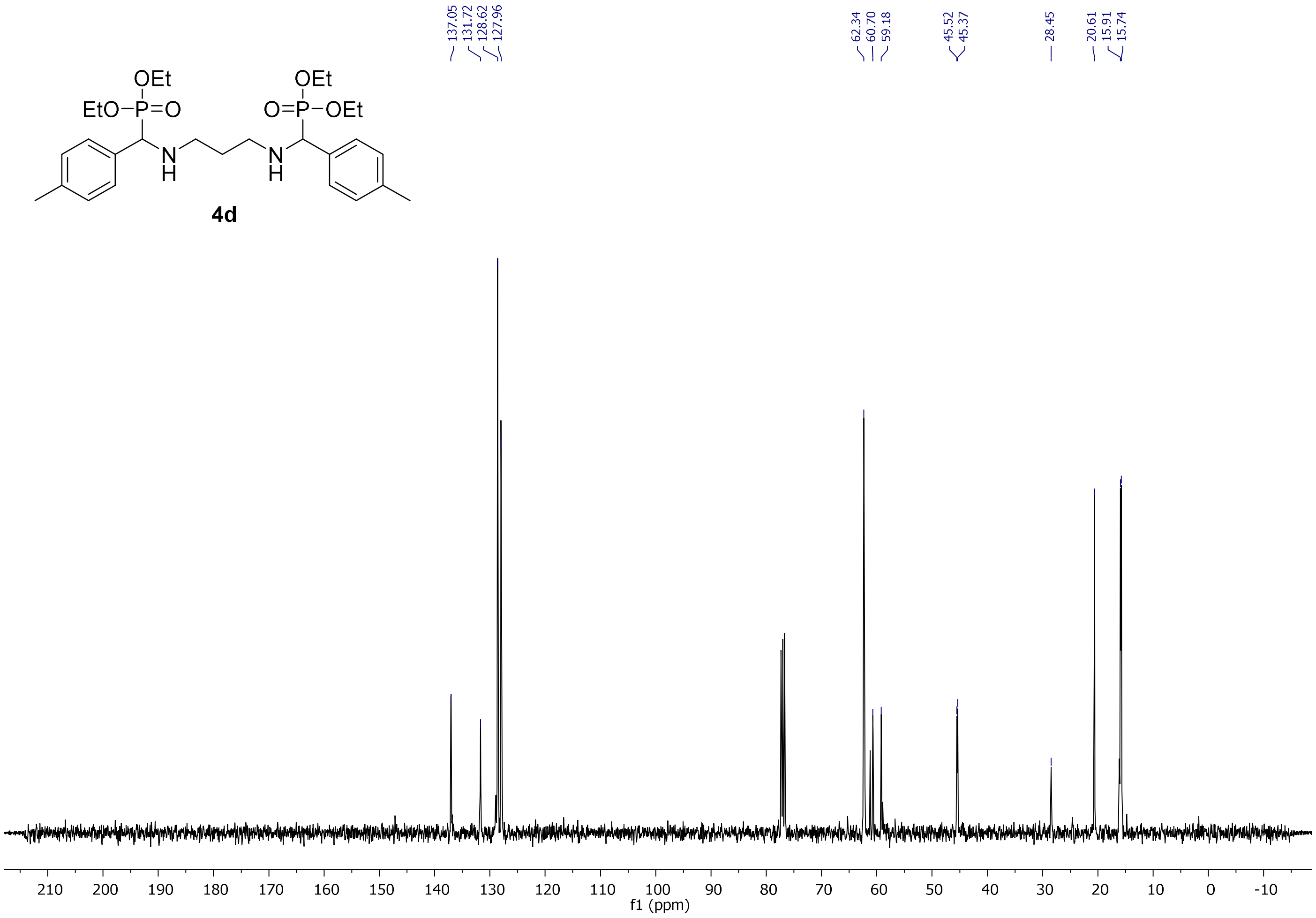

^{31}P NMR (162 MHz, $CDCl_3$) of compound **4d**.

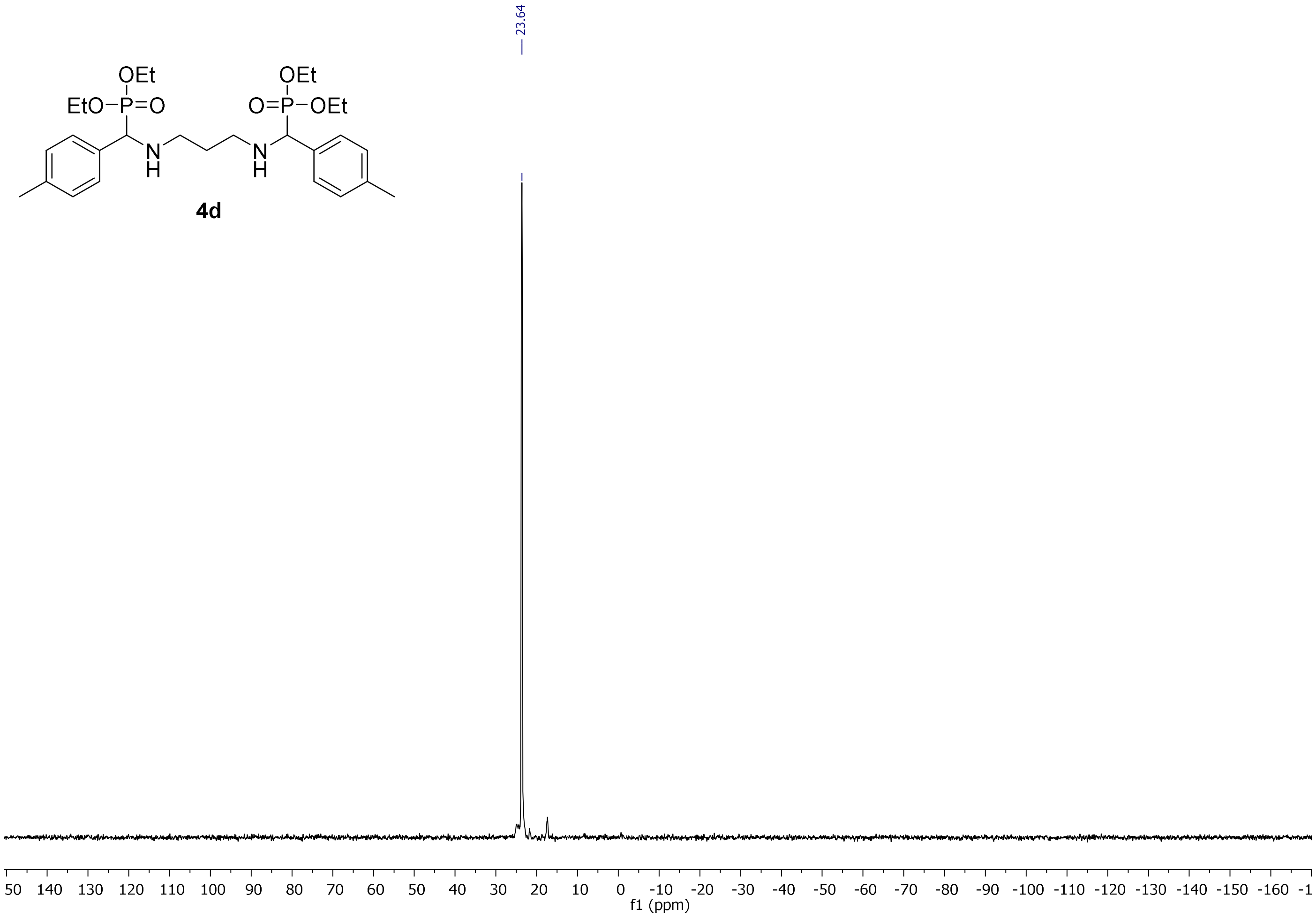

^{1}H NMR (400 MHz, $CDCl_3$) of compound **4e**.

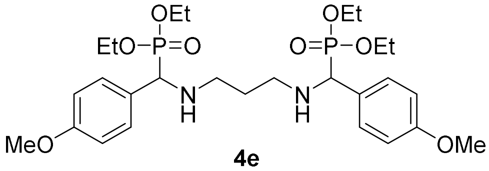

4e

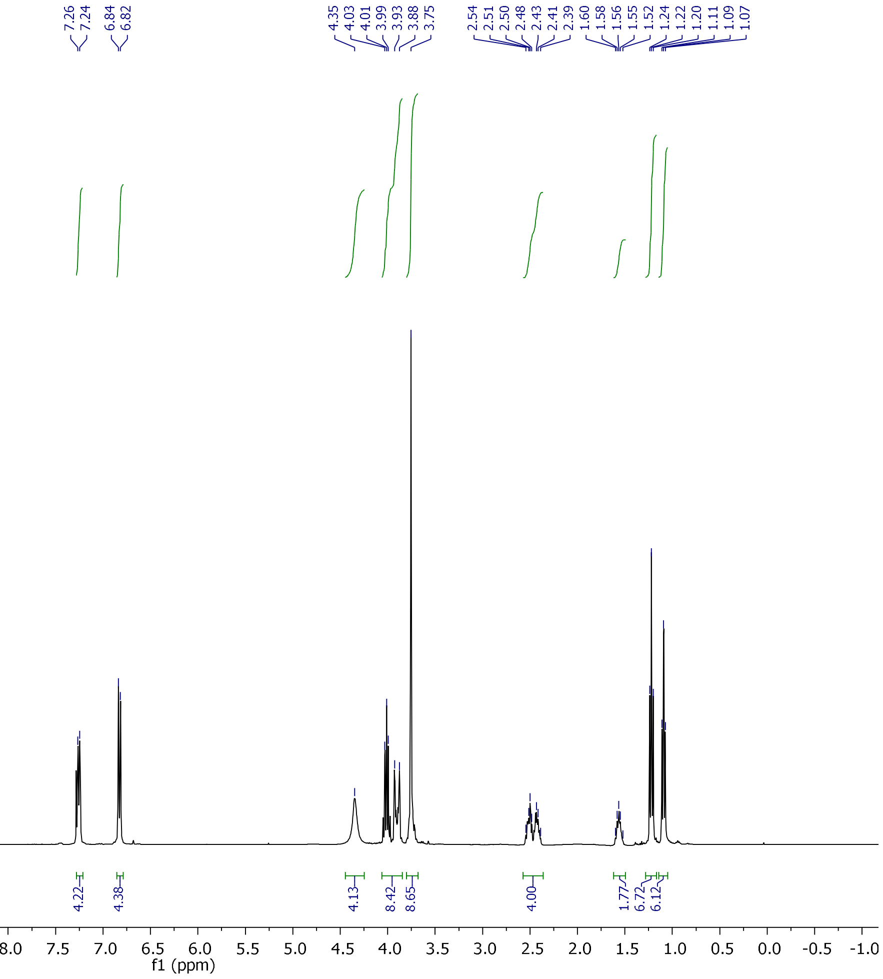

^{31}P NMR (162 MHz, $CDCl_3$) of compound **4e**.

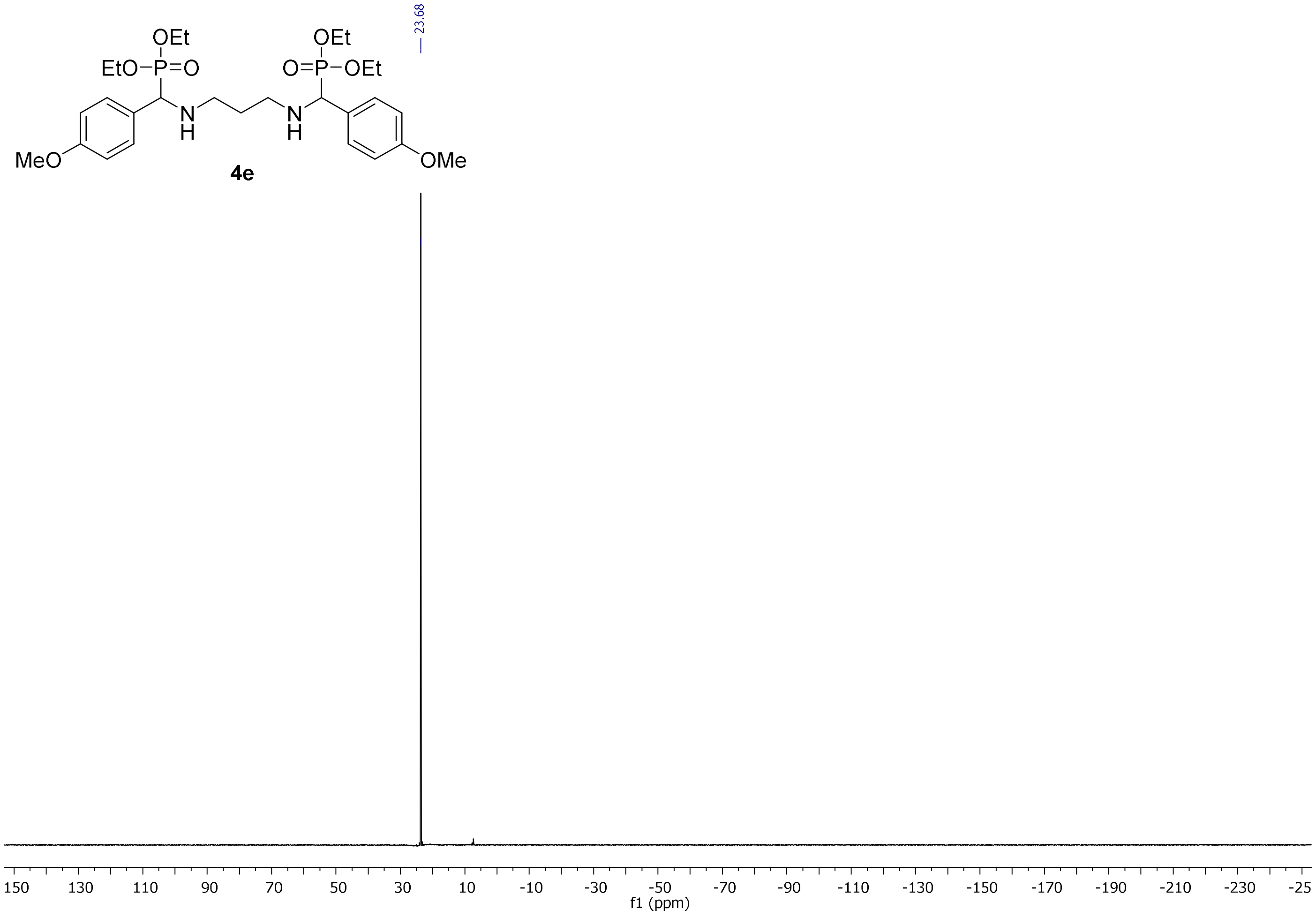

^{1}H NMR (400 MHz, $CDCl_3$) of compound **4f**.

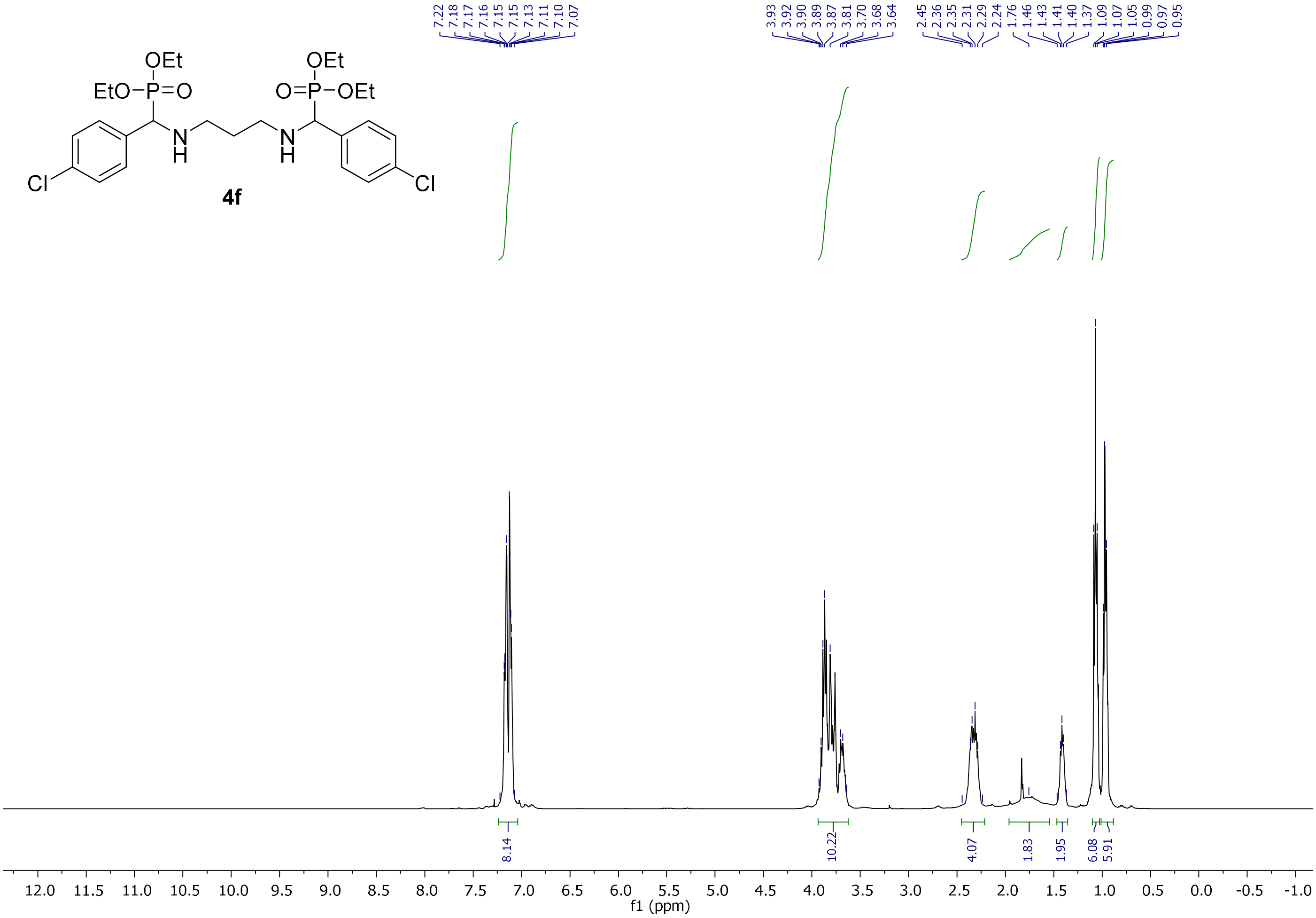

^{13}C {^{1}H} NMR (101 MHz, $CDCl_3$) of compound **4f**.

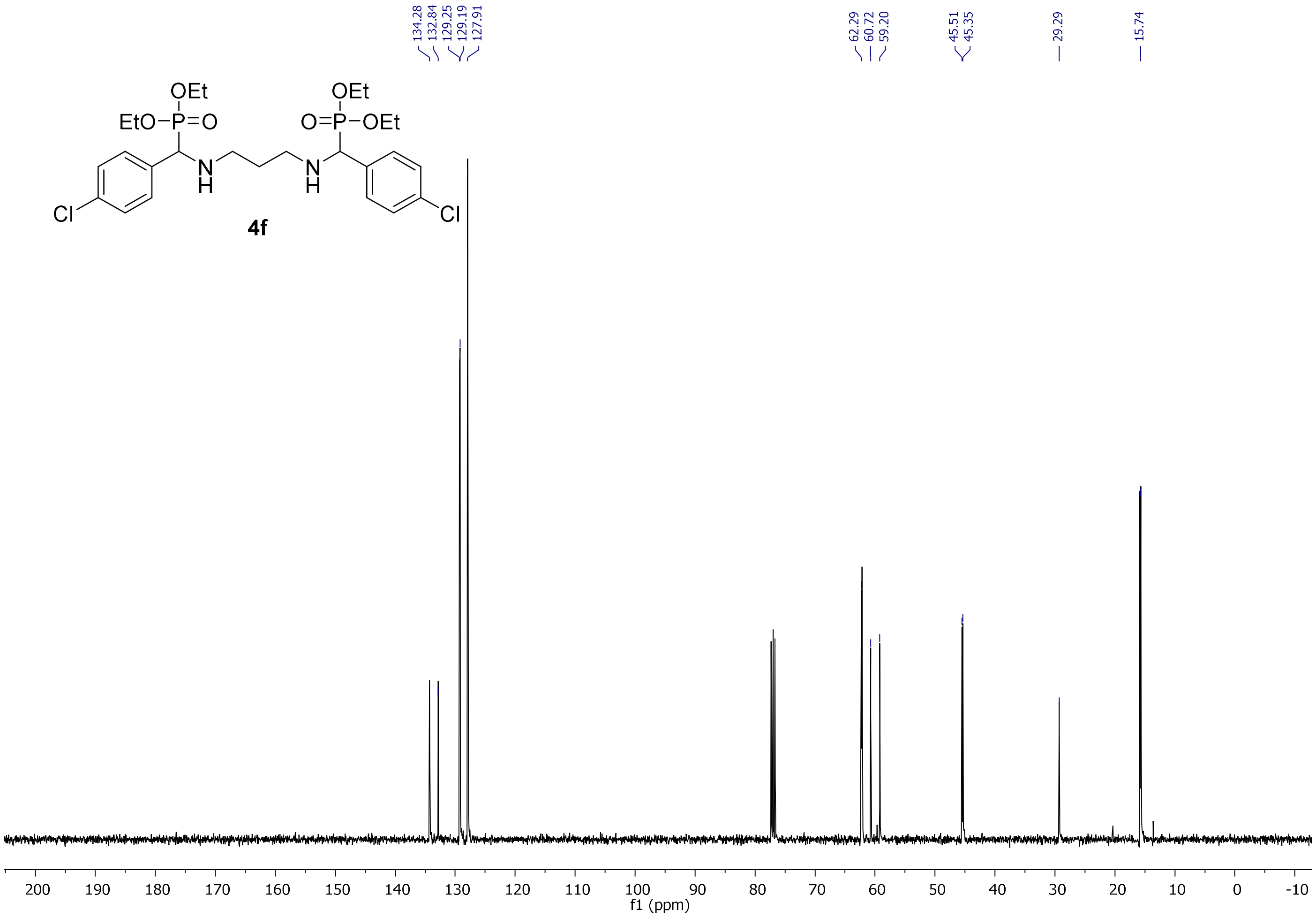

^{31}P NMR (162 MHz, $CDCl_3$) of compound **4f**.

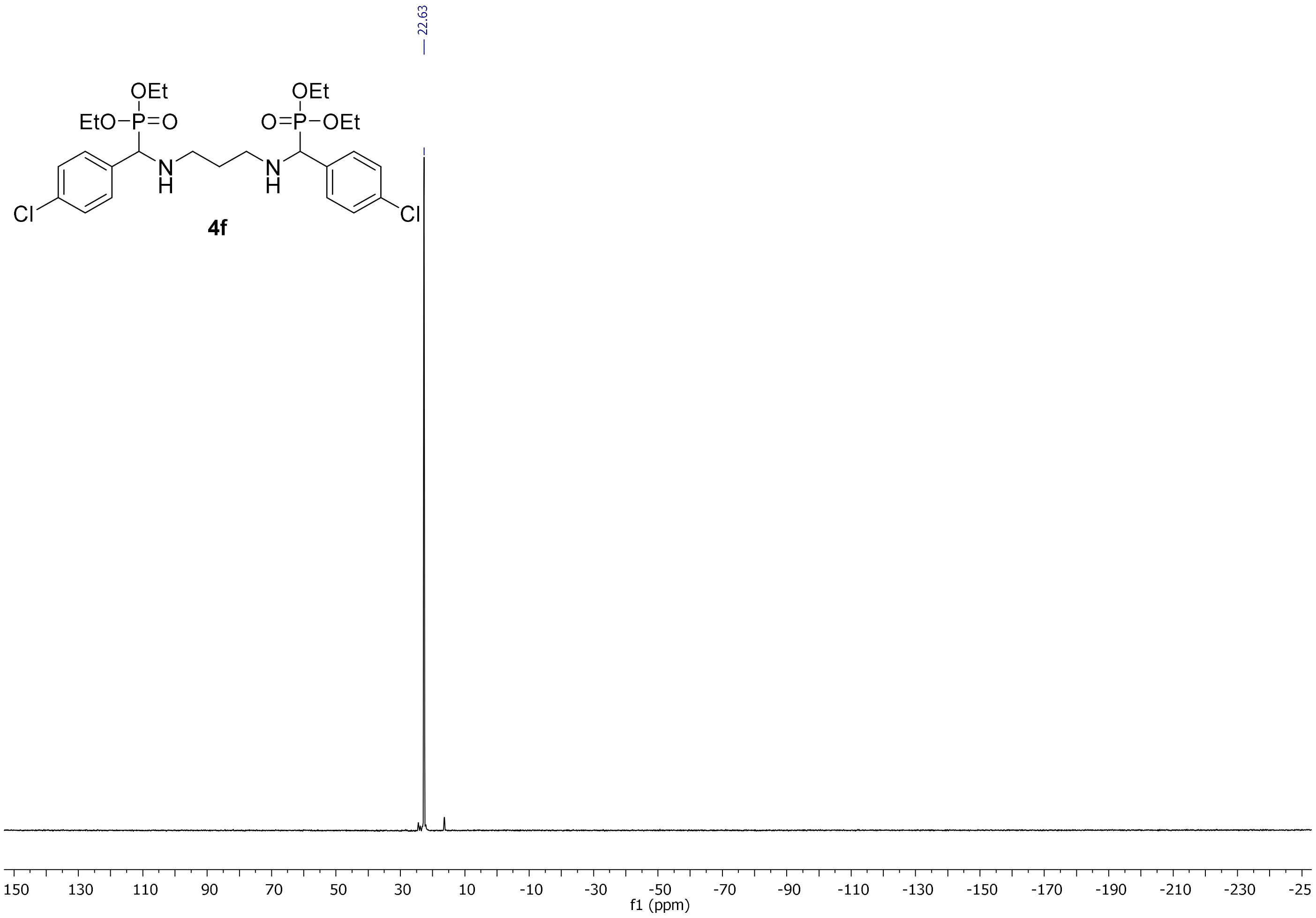

^{1}H NMR (400 MHz, $CDCl_3$) of compound **4g**.

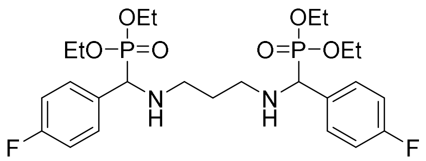

4g

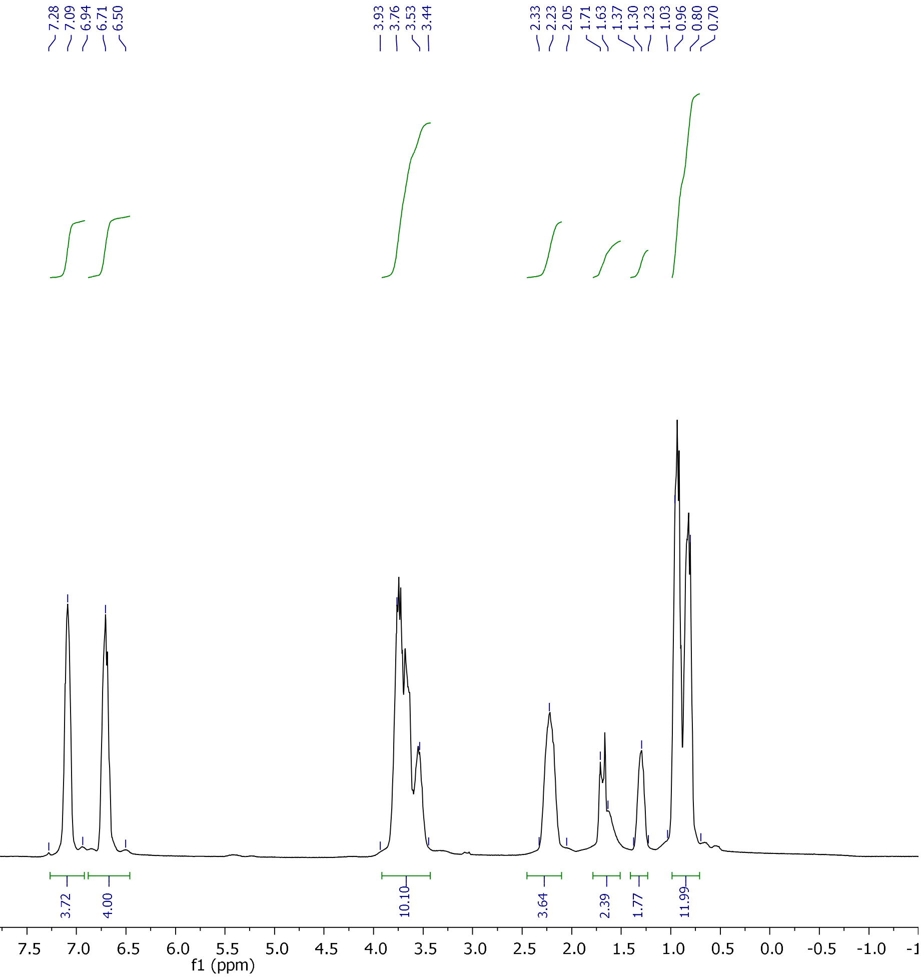

^{13}C {^{1}H} NMR (101 MHz, $CDCl_3$) of compound **4g**.

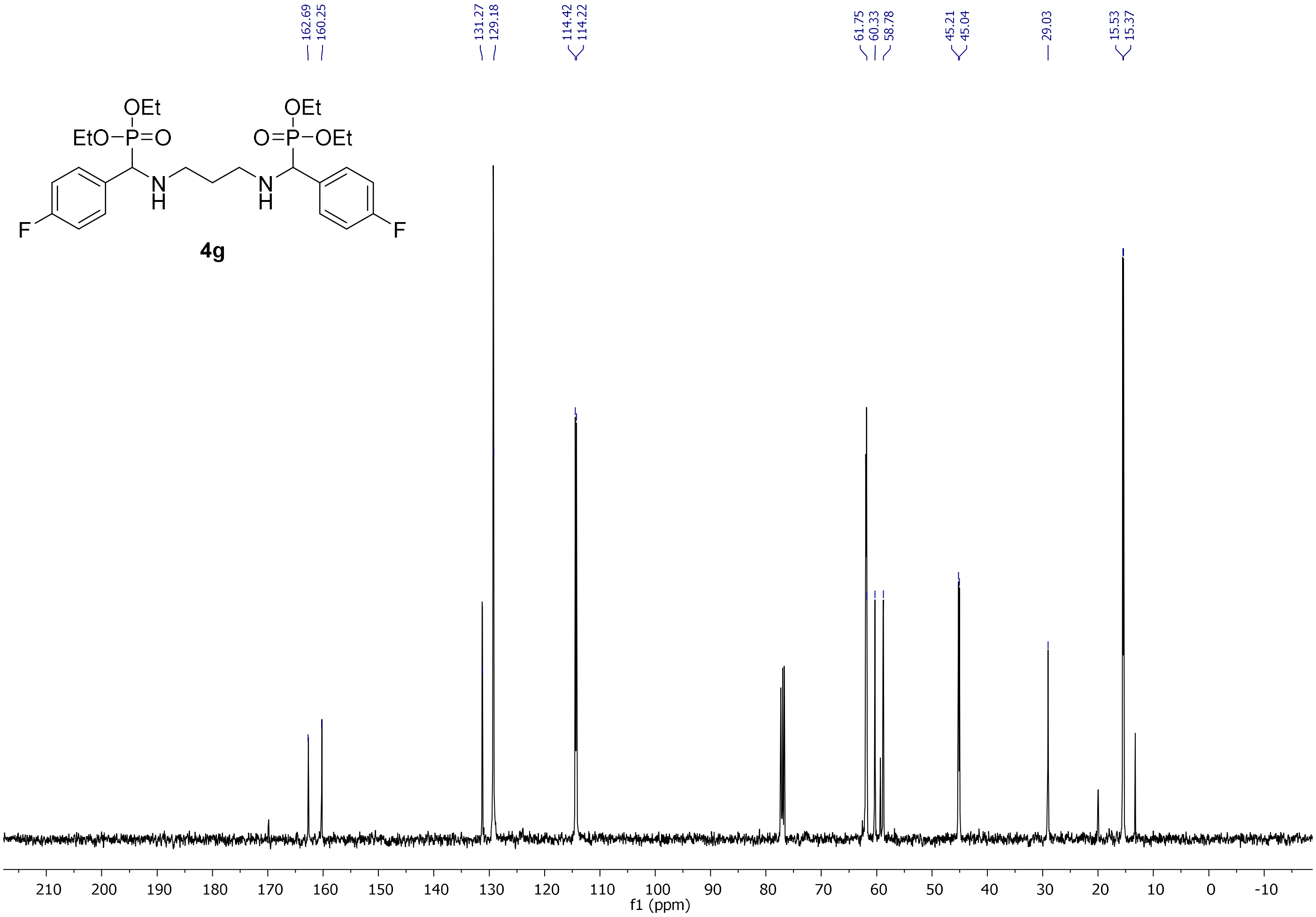

^{31}P NMR (162 MHz, $CDCl_3$) of compound **4g**.

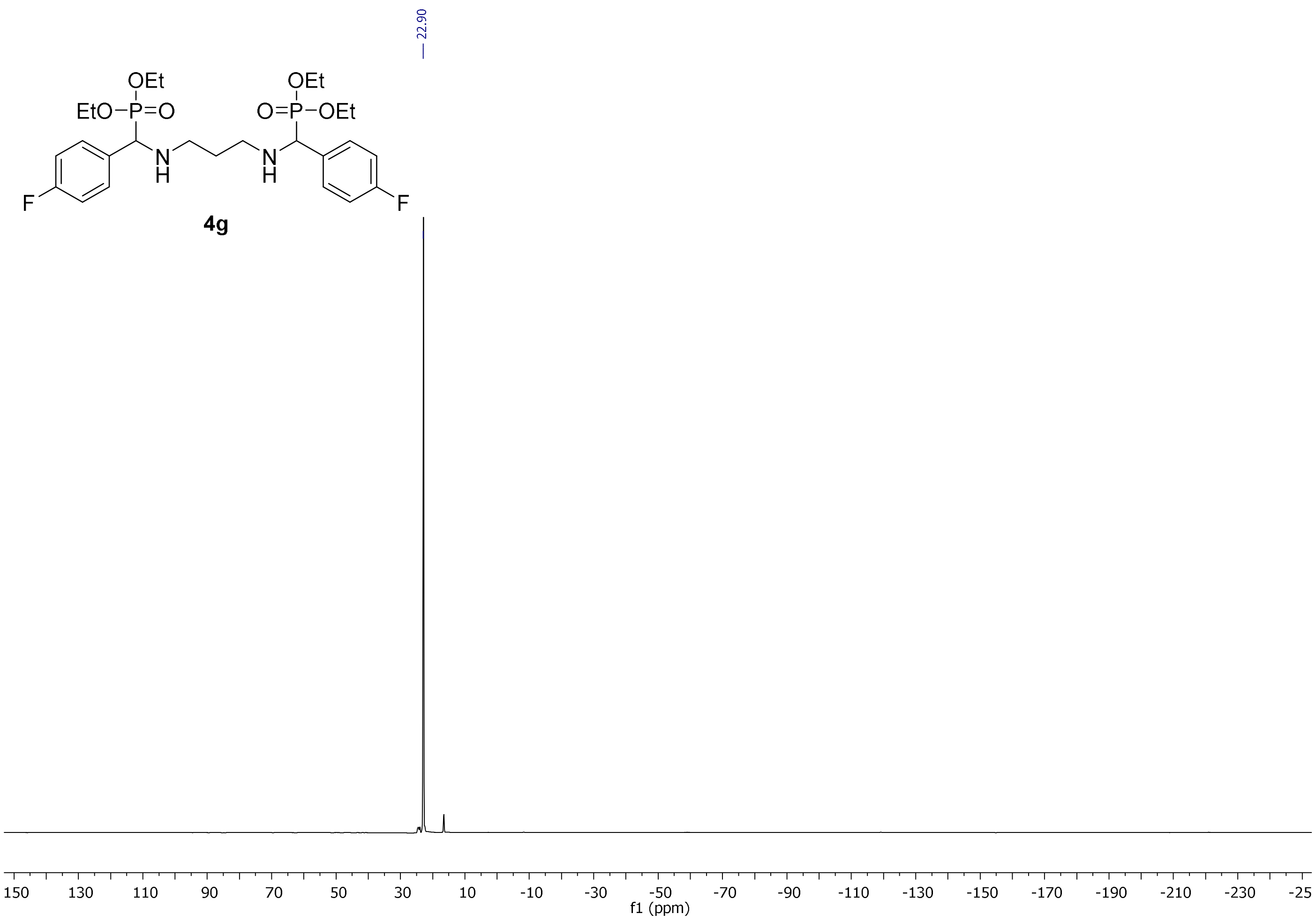

^{18}F NMR (376 MHz, $CDCl_3$) of compound **4g**.

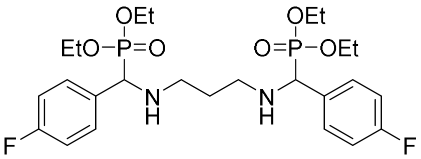

4g

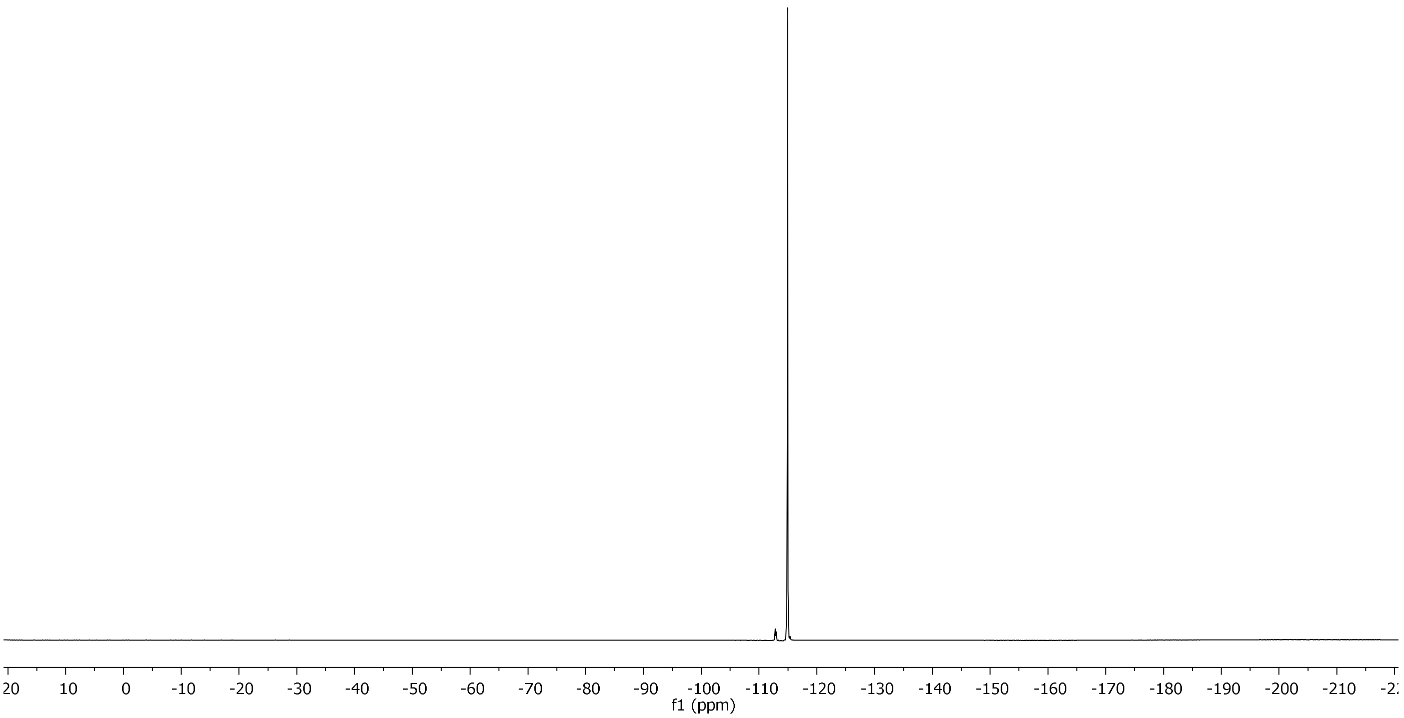

2D-COSY NMR {^{1}H-^{1}H} (400 MHz, $CDCl_3$) of compound **4g**.

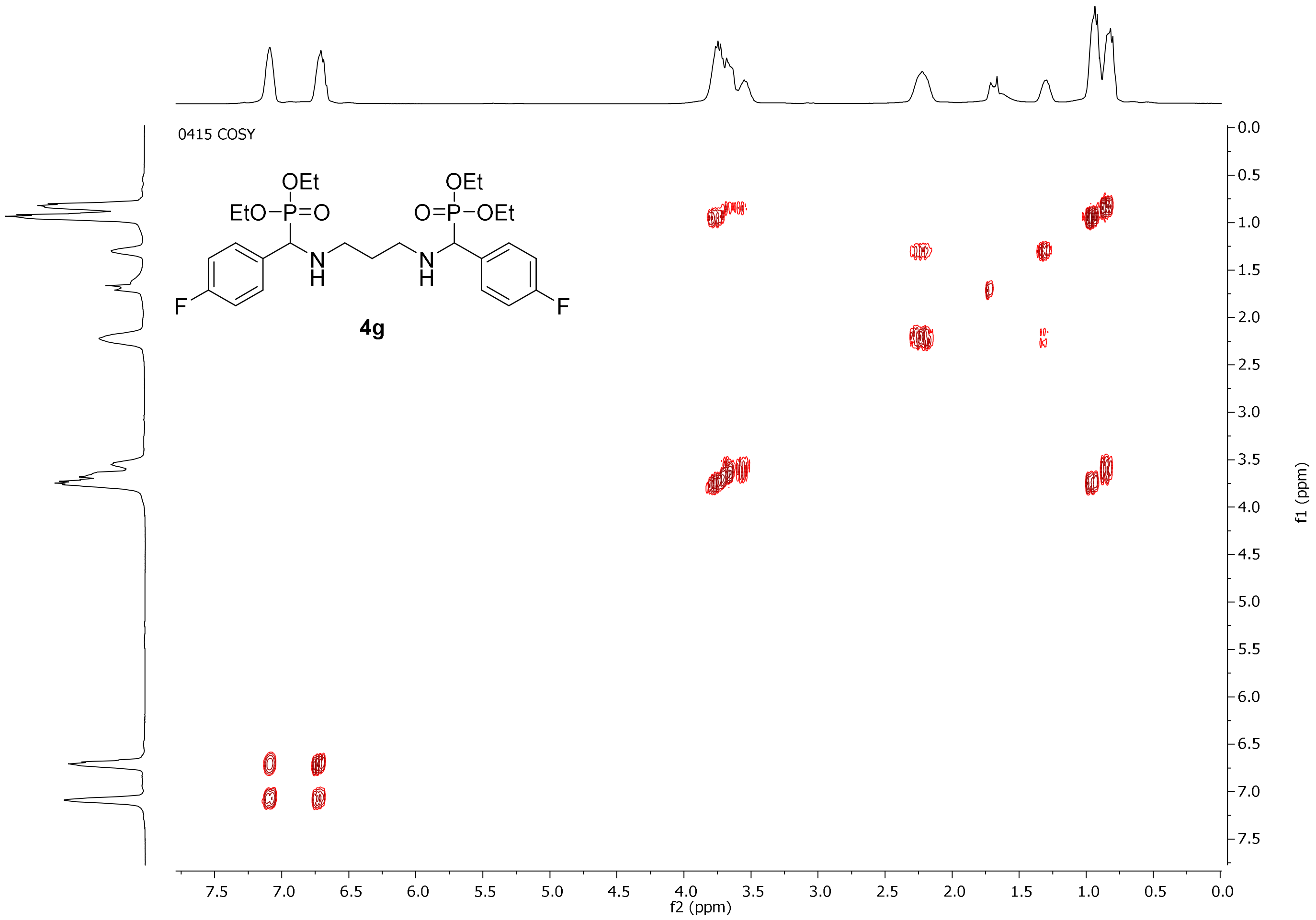

2D-HSQC NMR {^1H-^{13}C} (^1H: 400 MHz, ^{13}C: 101 MHz, $CDCl_3$) of compound **4g**.

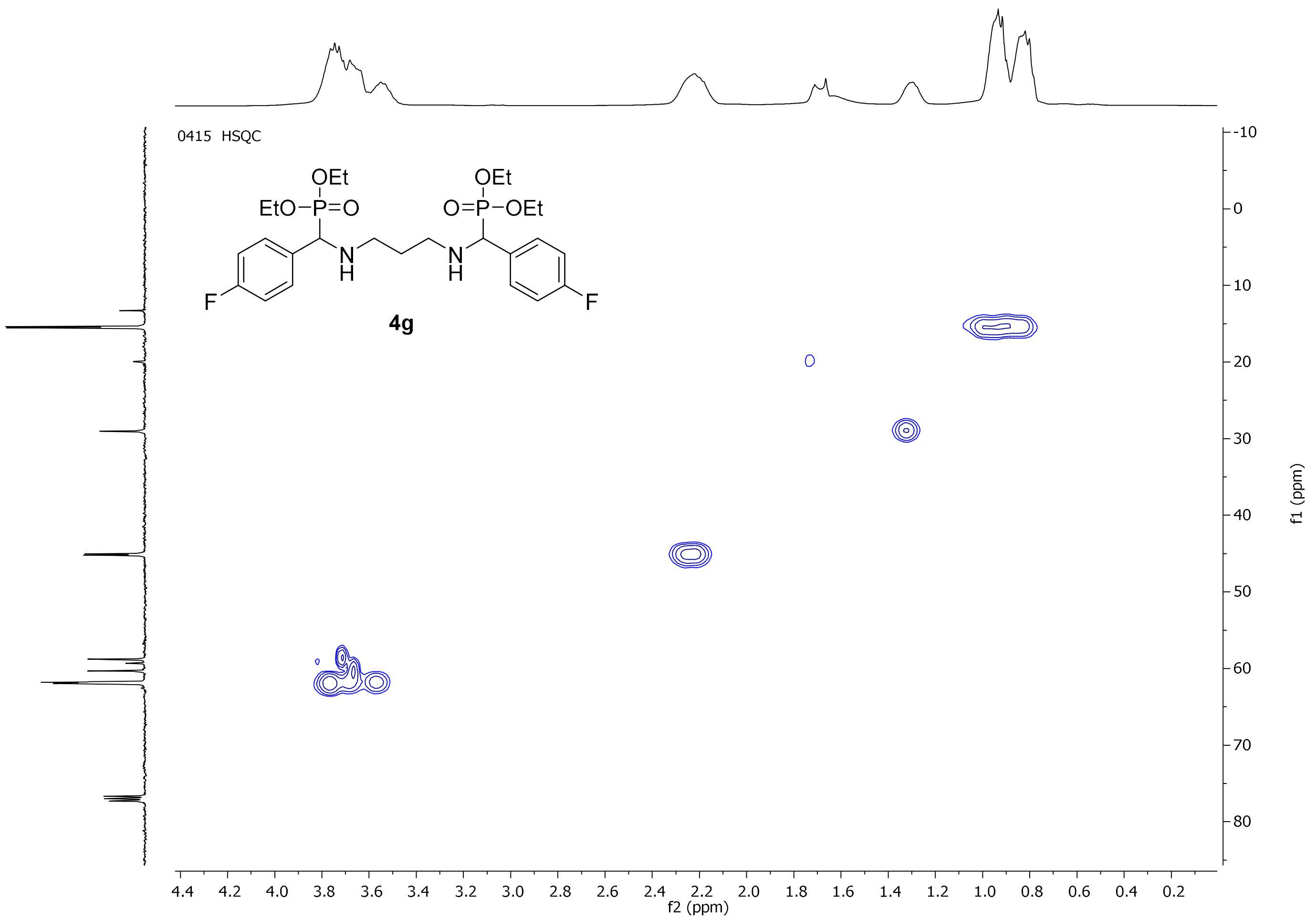

2D-HMBC NMR {^{1}H-^{13}C} (^{1}H: 400 MHz, ^{13}C: 101 MHz, $CDCl_3$) of compound **4g**.

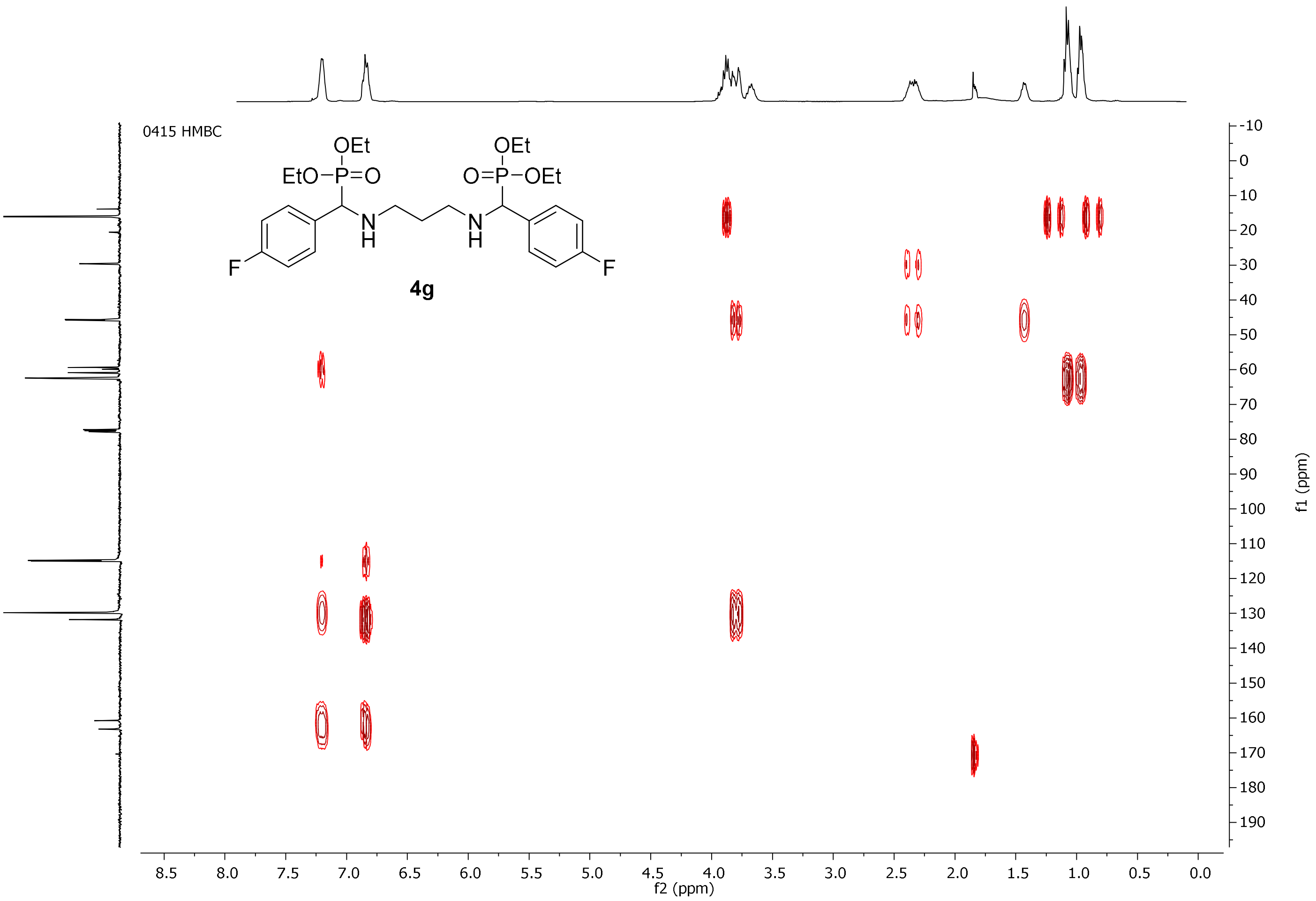

^{1}H NMR (400 MHz, $CDCl_3$) of compound **4h**.

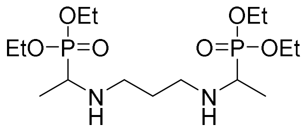

4h

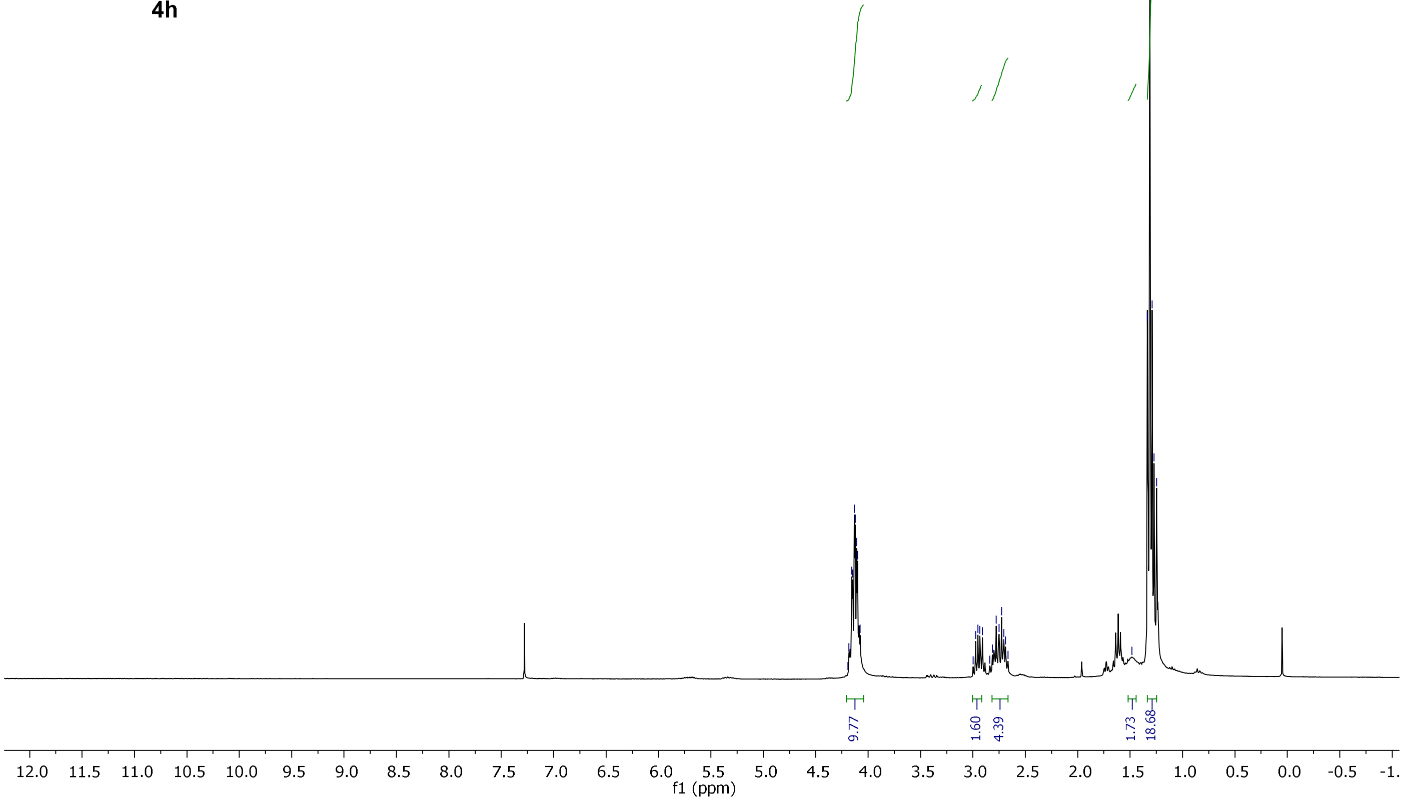

^{13}C {^{1}H} NMR (101 MHz, $CDCl_3$) of compound **4h**.

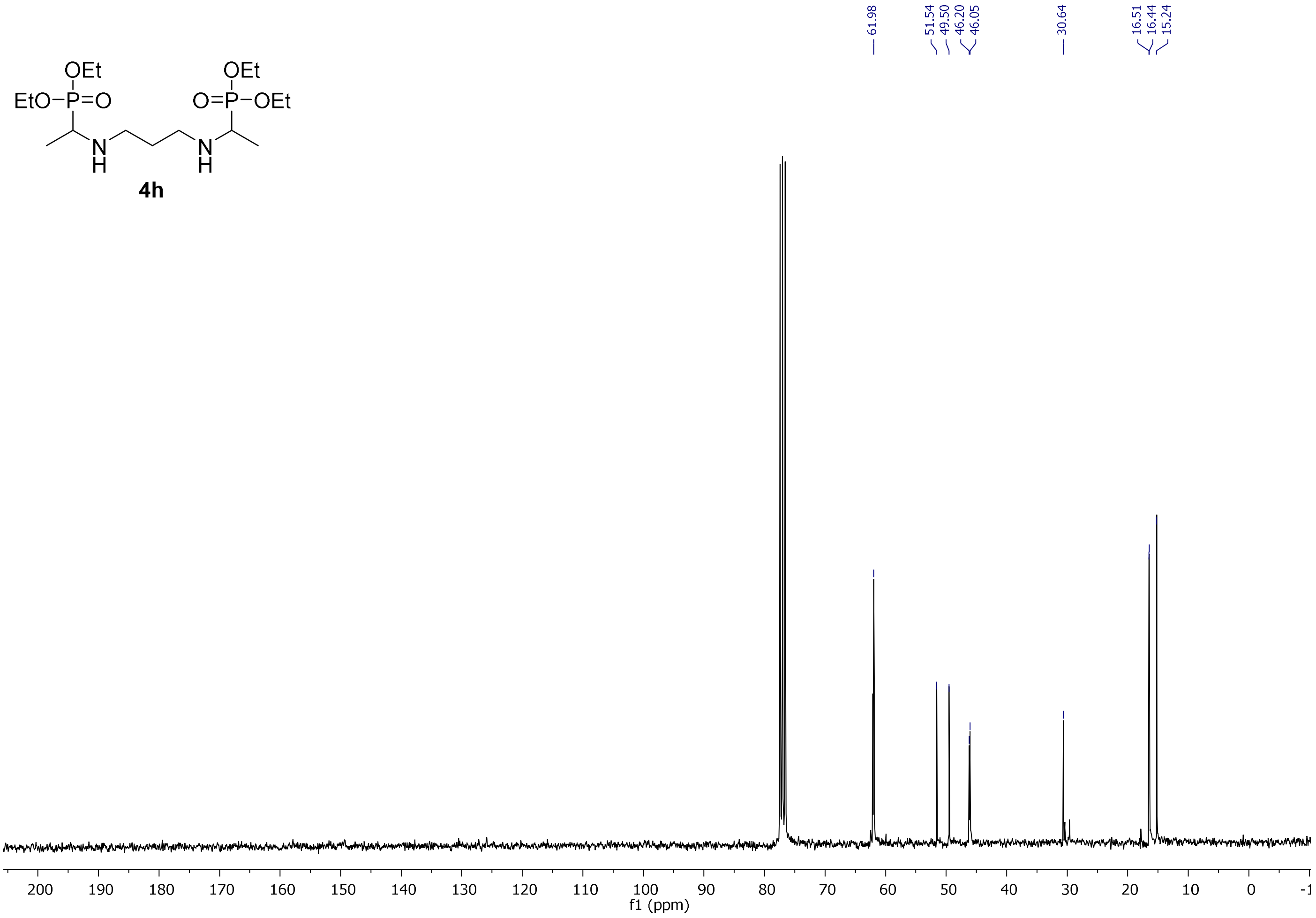

^{31}P NMR (162 MHz, $CDCl_3$) of compound **4h**.

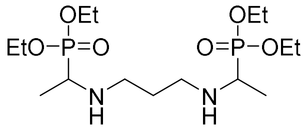

4h

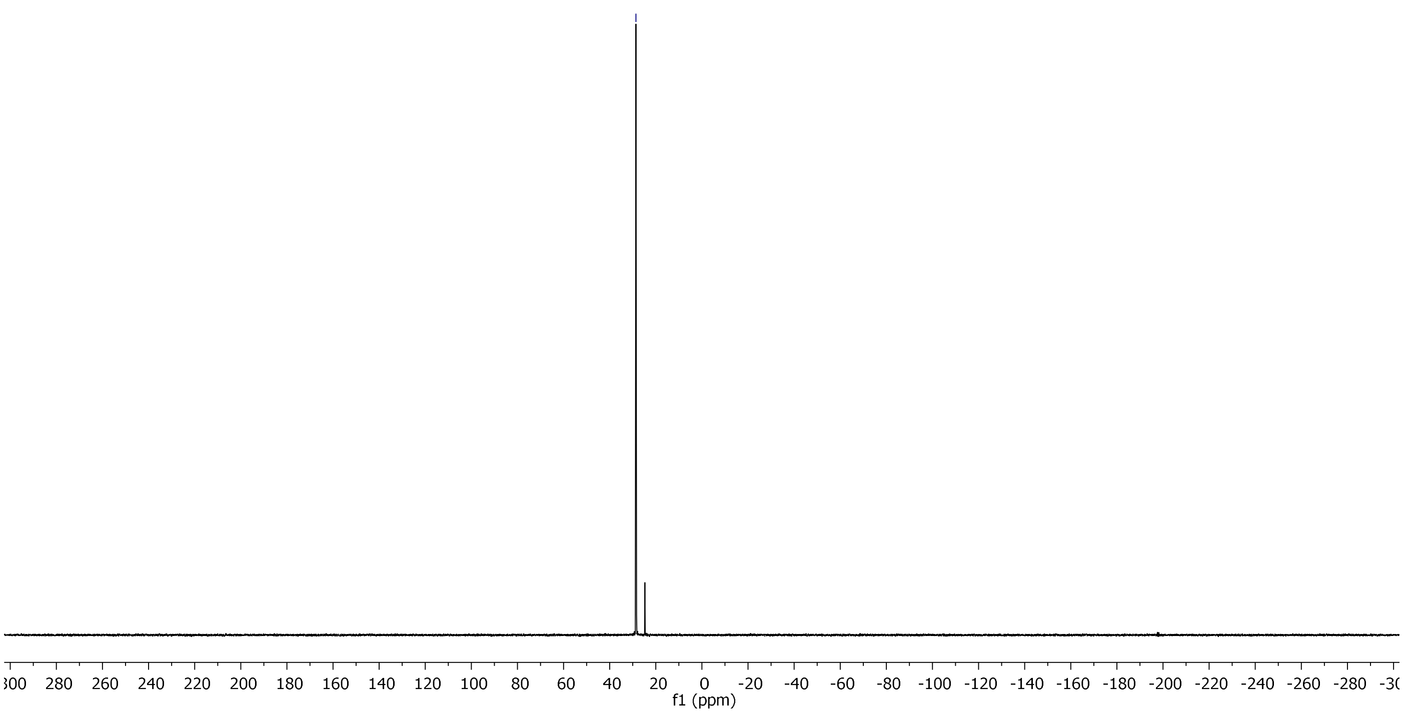

^{1}H NMR (300 MHz, $CDCl_3$) of compound **6a**.

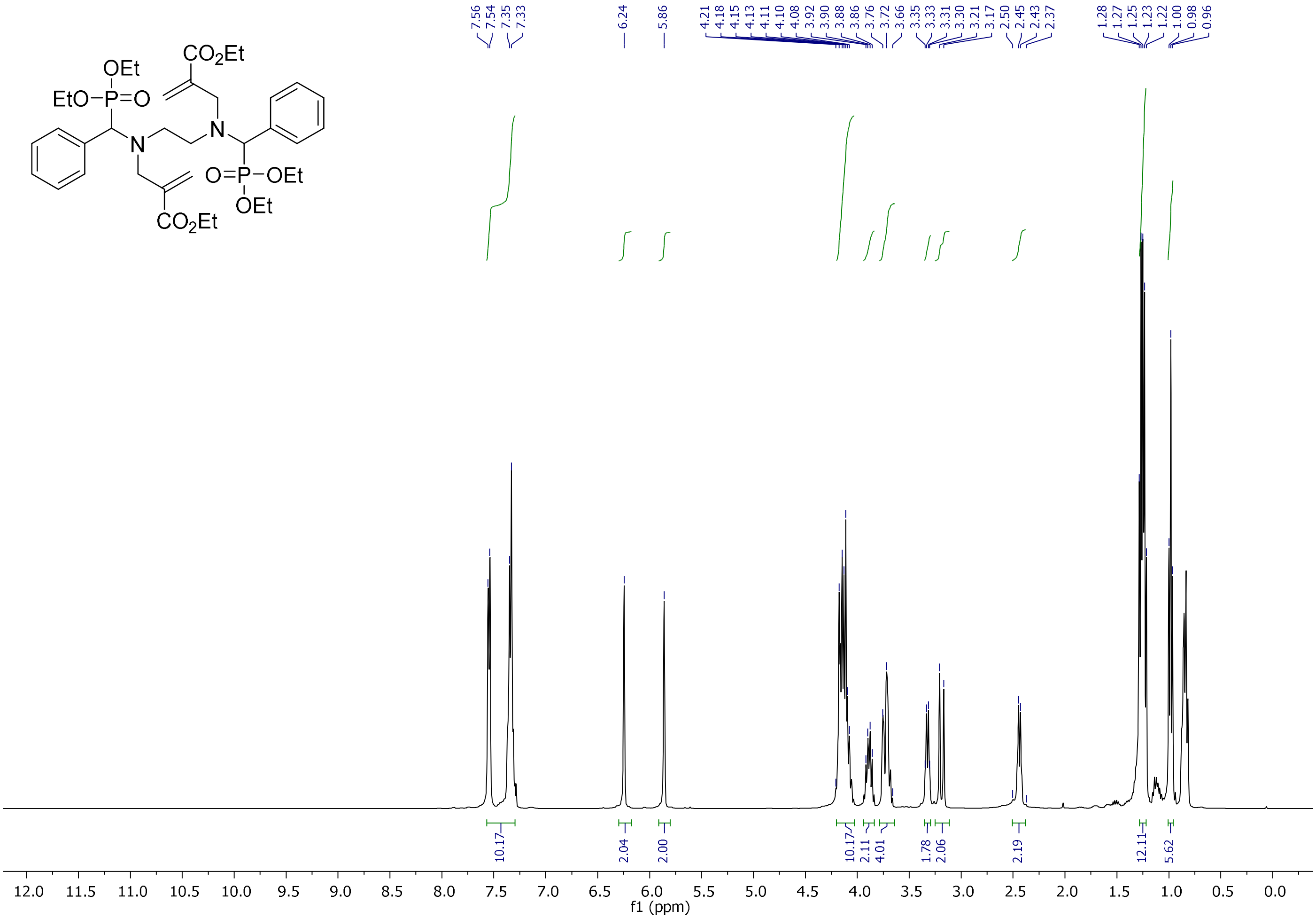

^{13}C {^{1}H} NMR (101 MHz, $CDCl_3$) of compound **6a**.

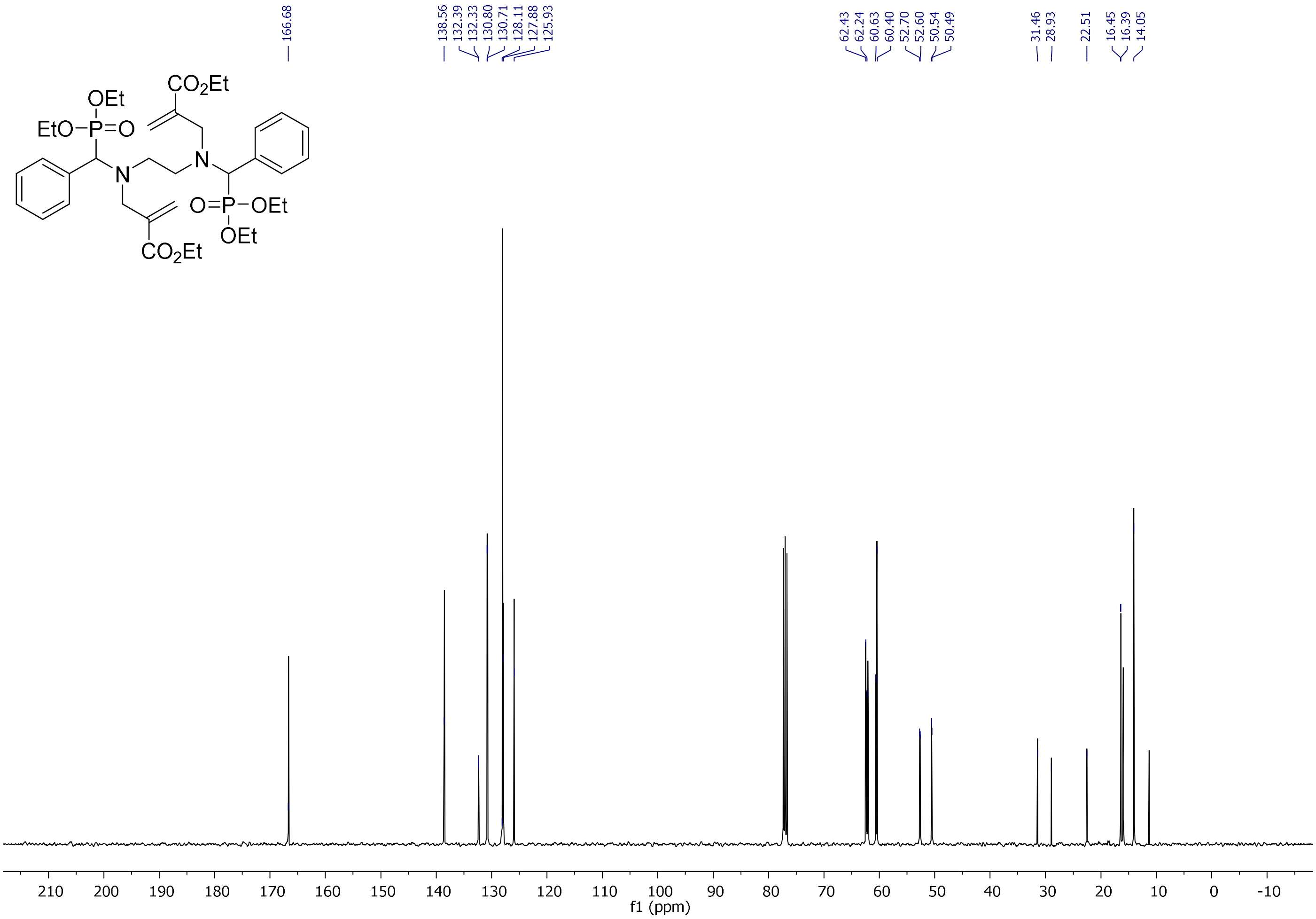

^{31}P NMR (162 MHz, $CDCl_3$) of compound **6a**.

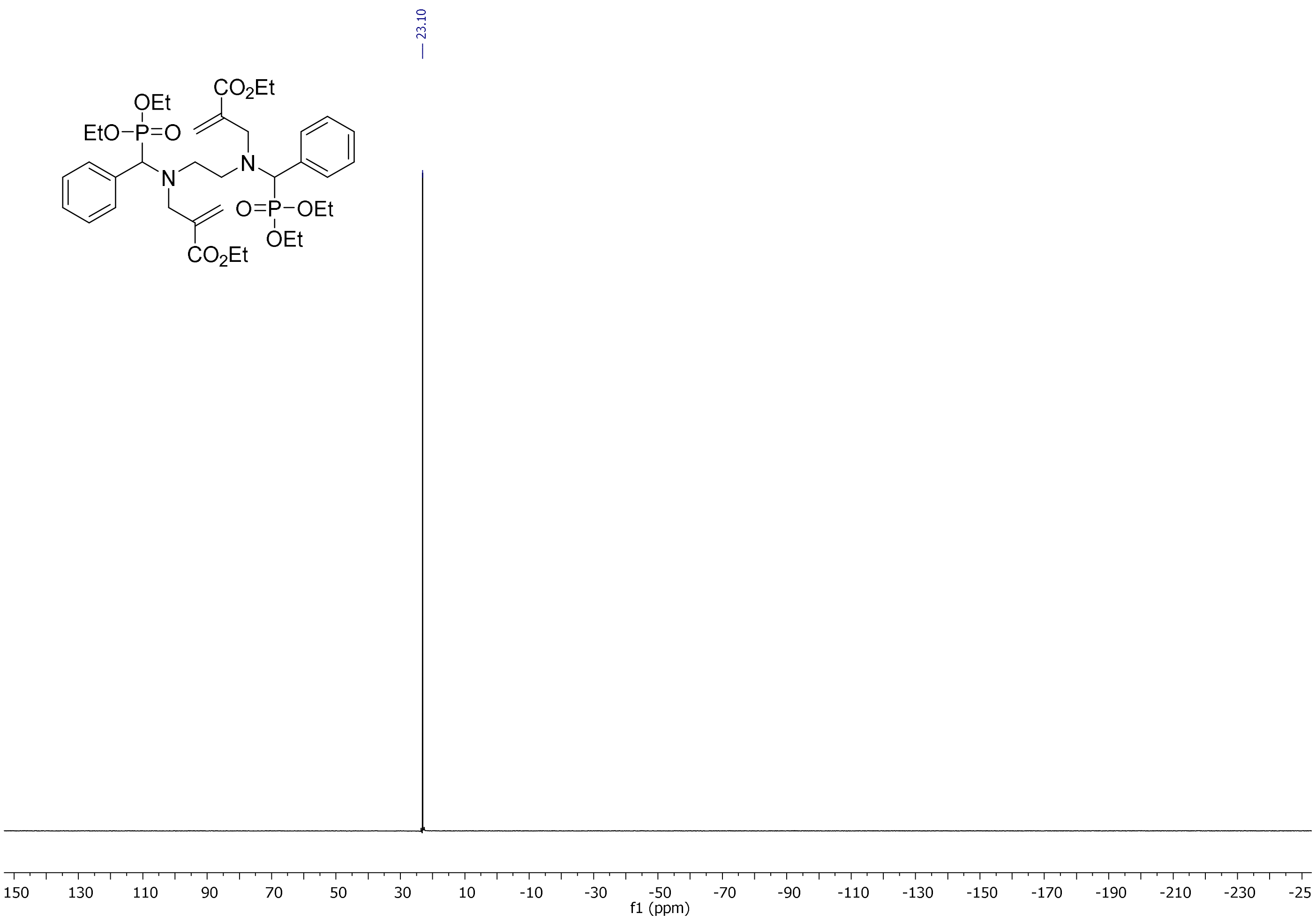

^{1}H NMR (400 MHz, $CDCl_3$) of compound **6b**.

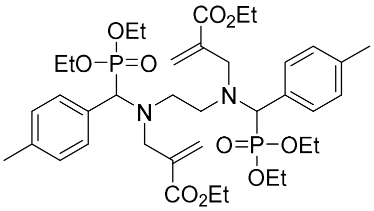

6b

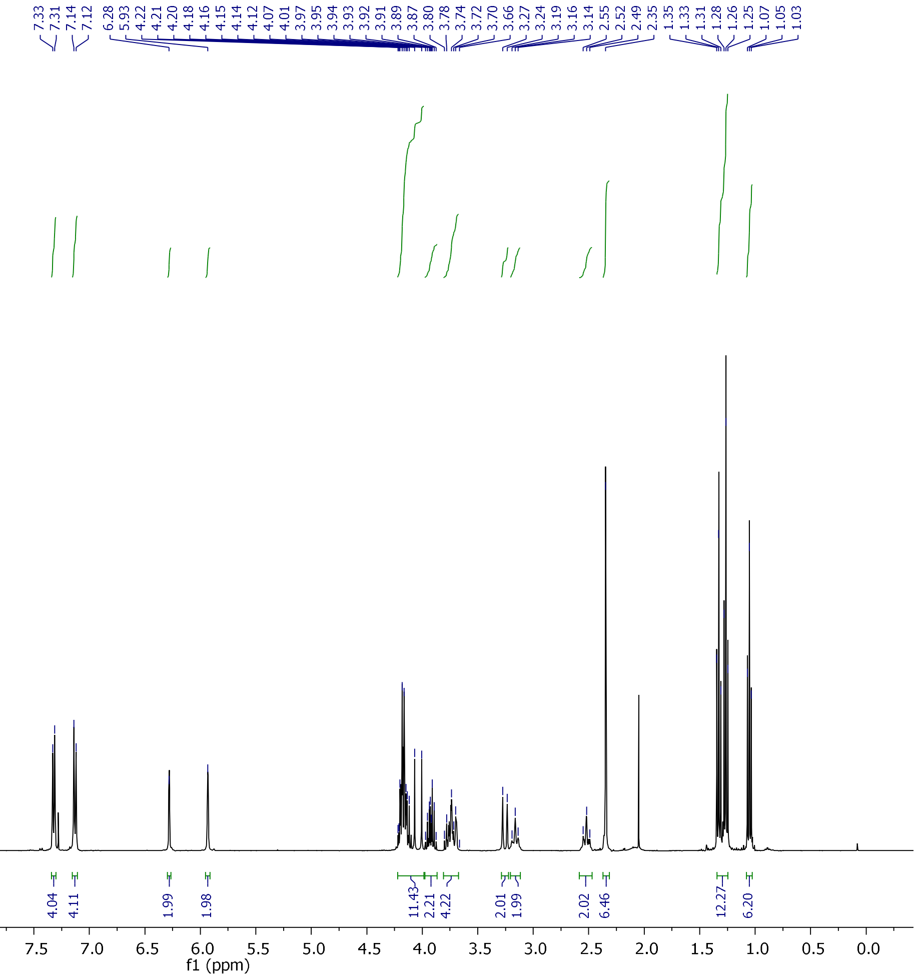

^{13}C {^{1}H} NMR (101 MHz, $CDCl_3$) of compound **6b**.

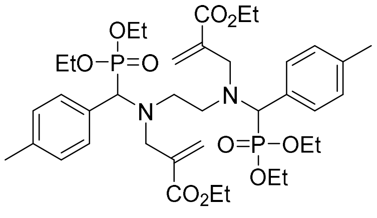

6b

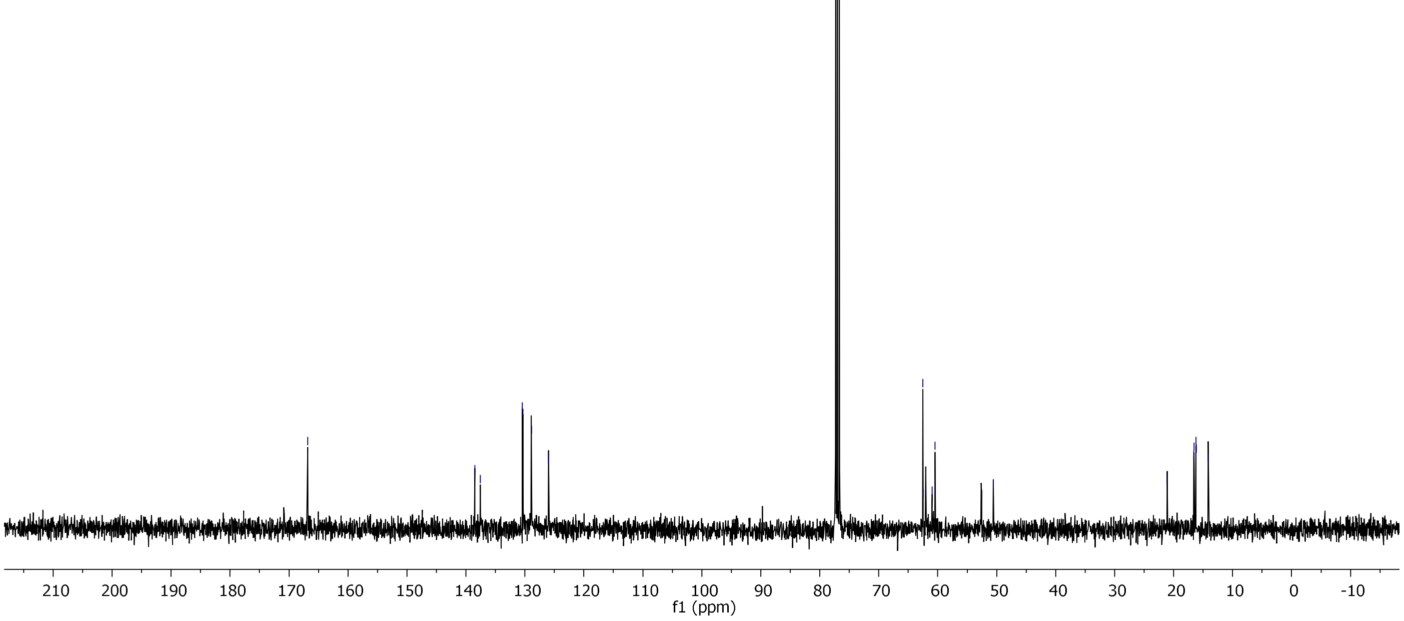

^{31}P NMR (162 MHz, $CDCl_3$) of compound **6b**.

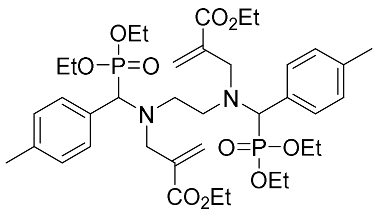

6b

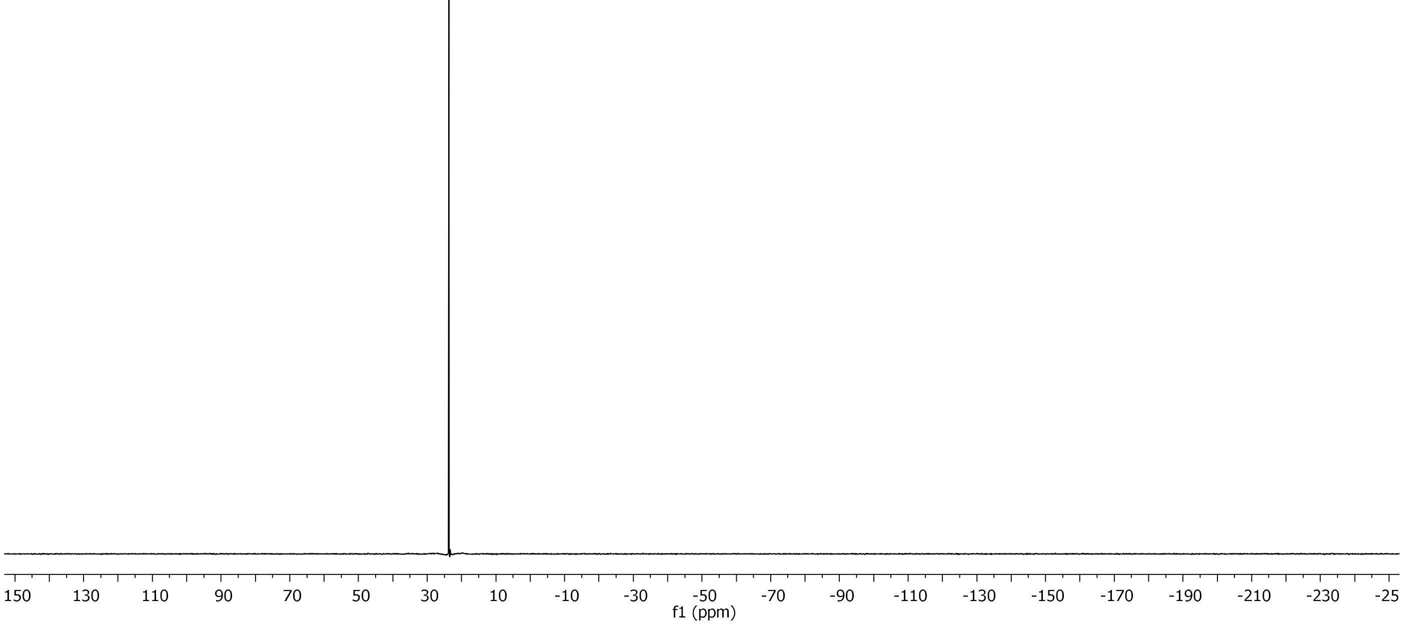

¹H NMR (300 MHz, $CDCl_3$) of compound **6c**.

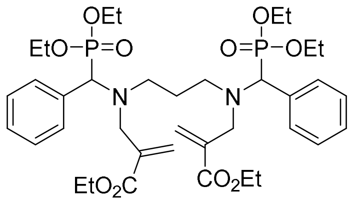

6c

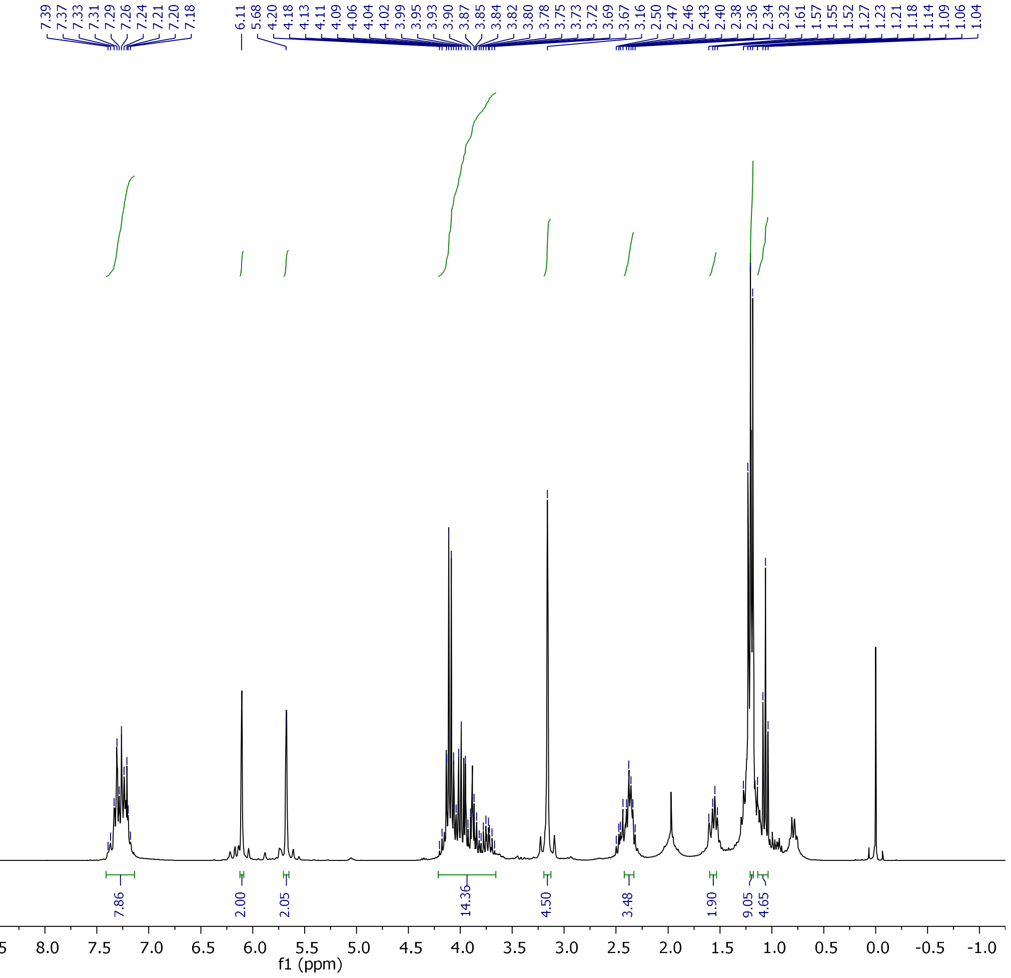

^{13}C {^{1}H} NMR (75 MHz, $CDCl_3$) of compound **6c**.

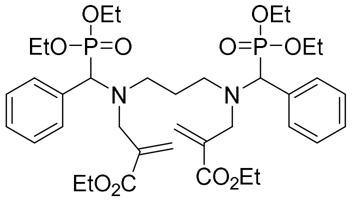

6c

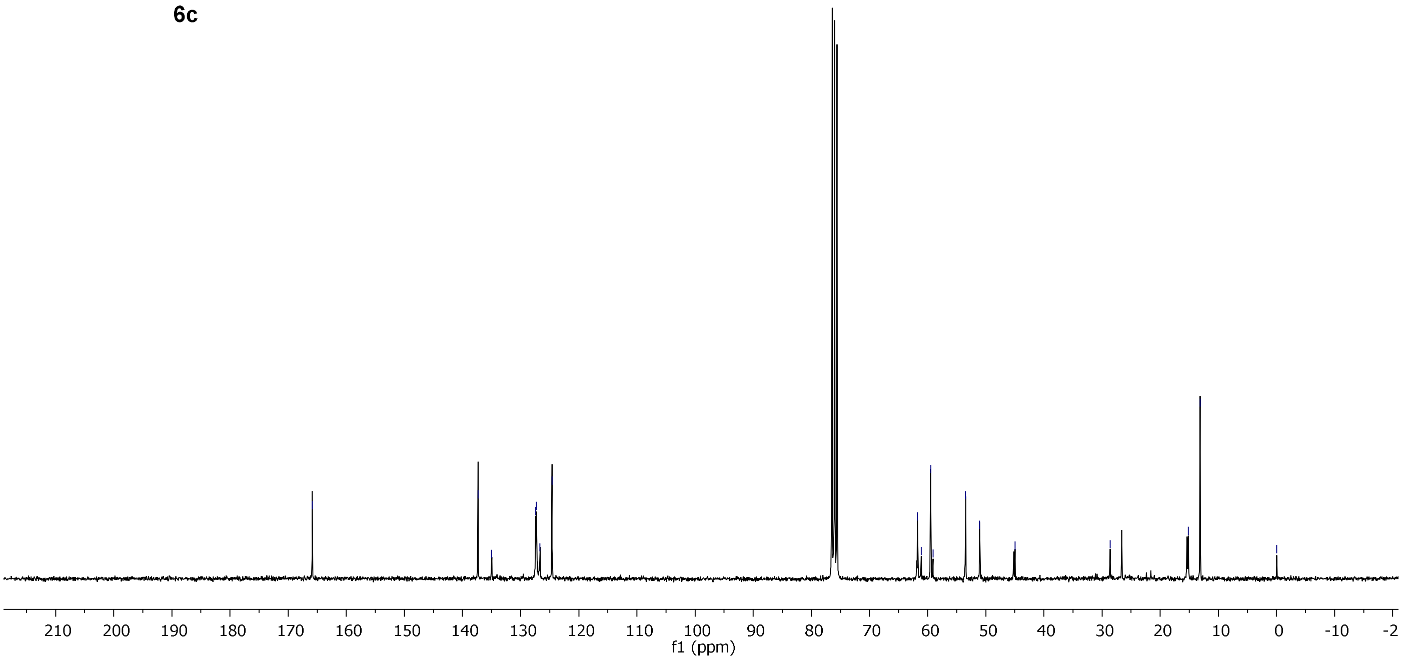

^{31}P NMR (121 MHz, $CDCl_3$) of compound **6c**.

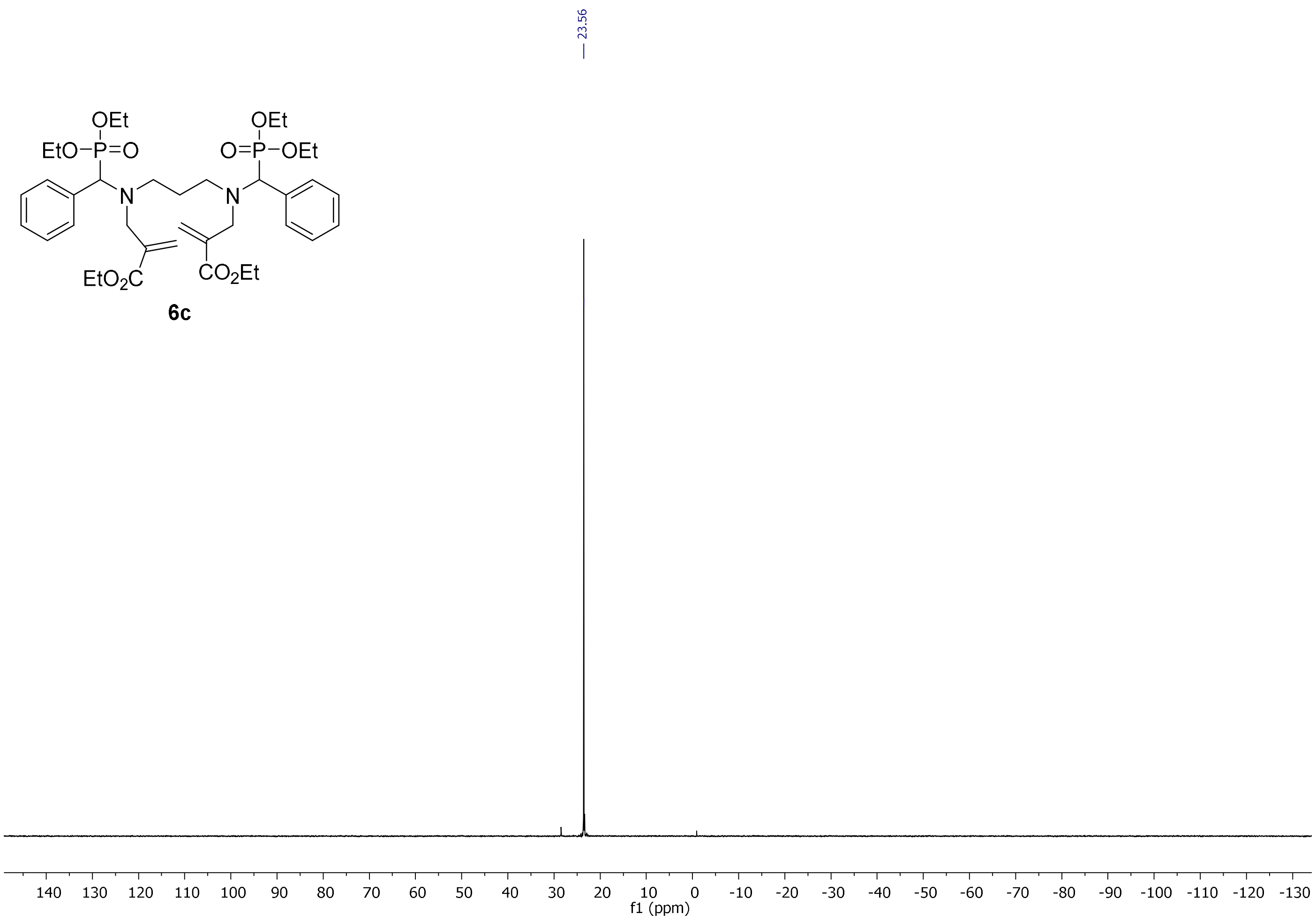

^1H NMR (300 MHz, $CDCl_3$) of compound **6d**.

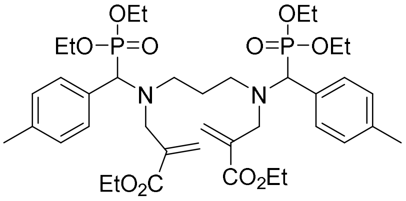

6d

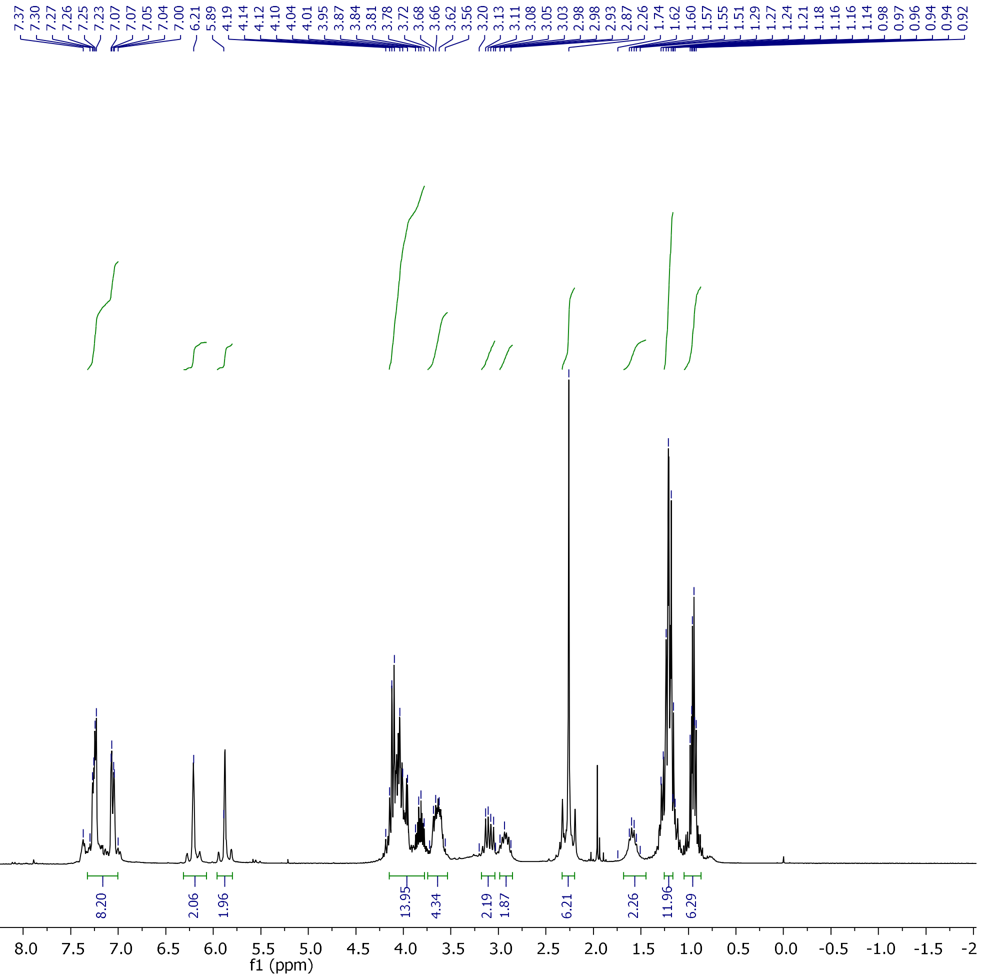

$^{13}C\{^1H\}$ NMR (101 MHz, $CDCl_3$) of compound **6d**.

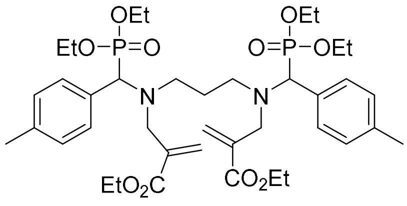

6d

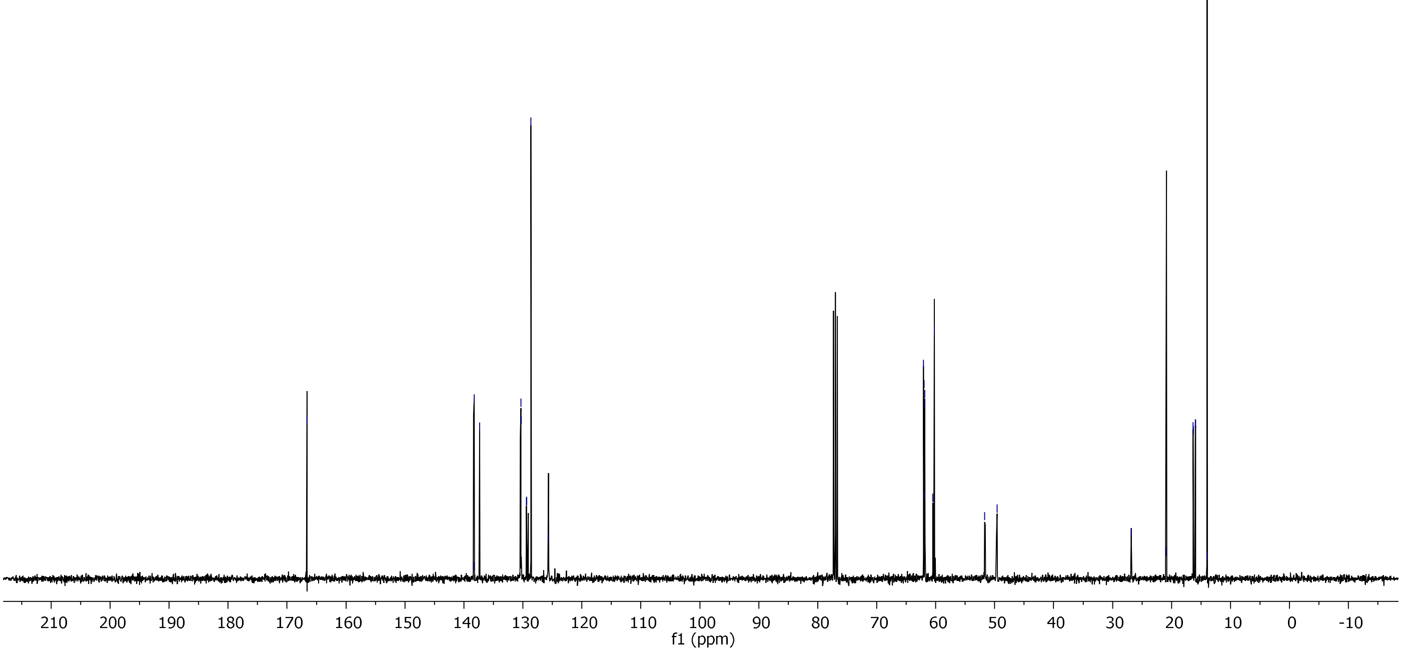

^{31}P NMR (121 MHz, $CDCl_3$) of compound **6d**.

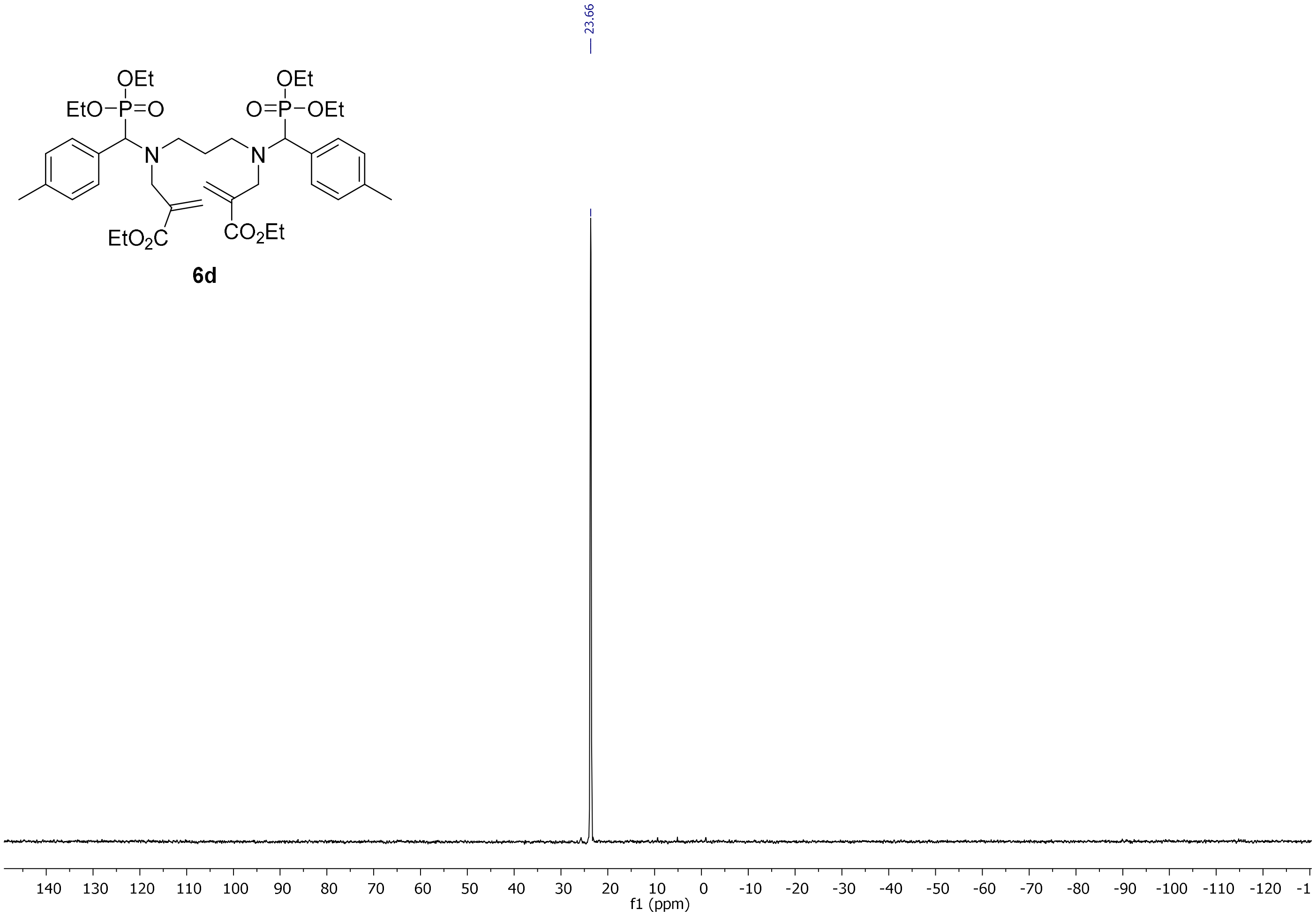

¹H NMR (400 MHz, $CDCl_3$) of compound **6e**.

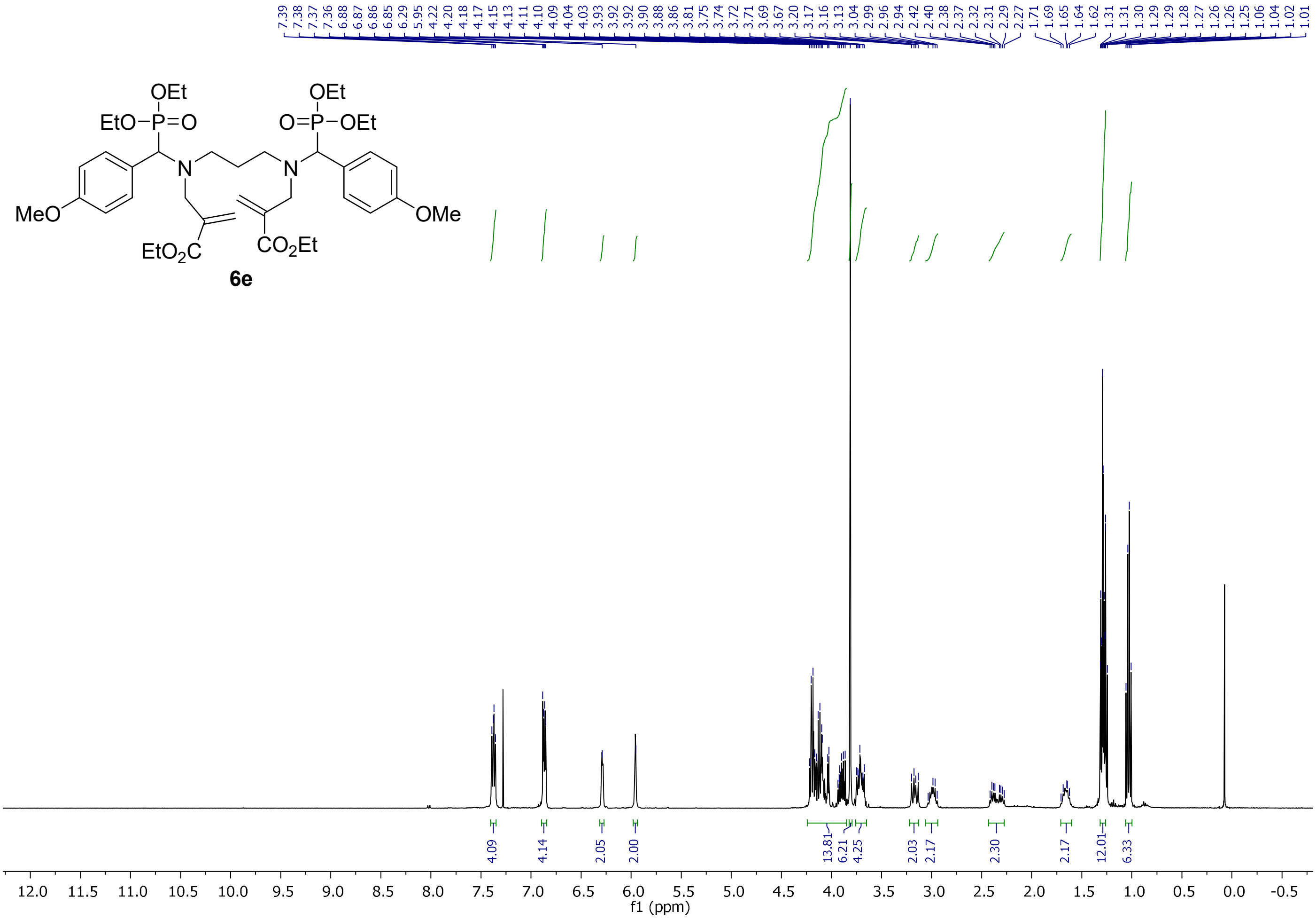

^{13}C {^{1}H} NMR (101 MHz, $CDCl_3$) of compound **6e**.

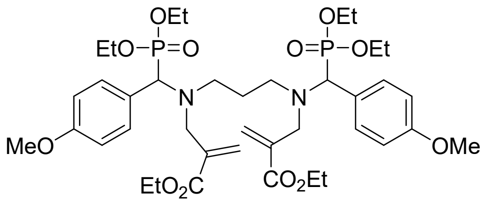

6e

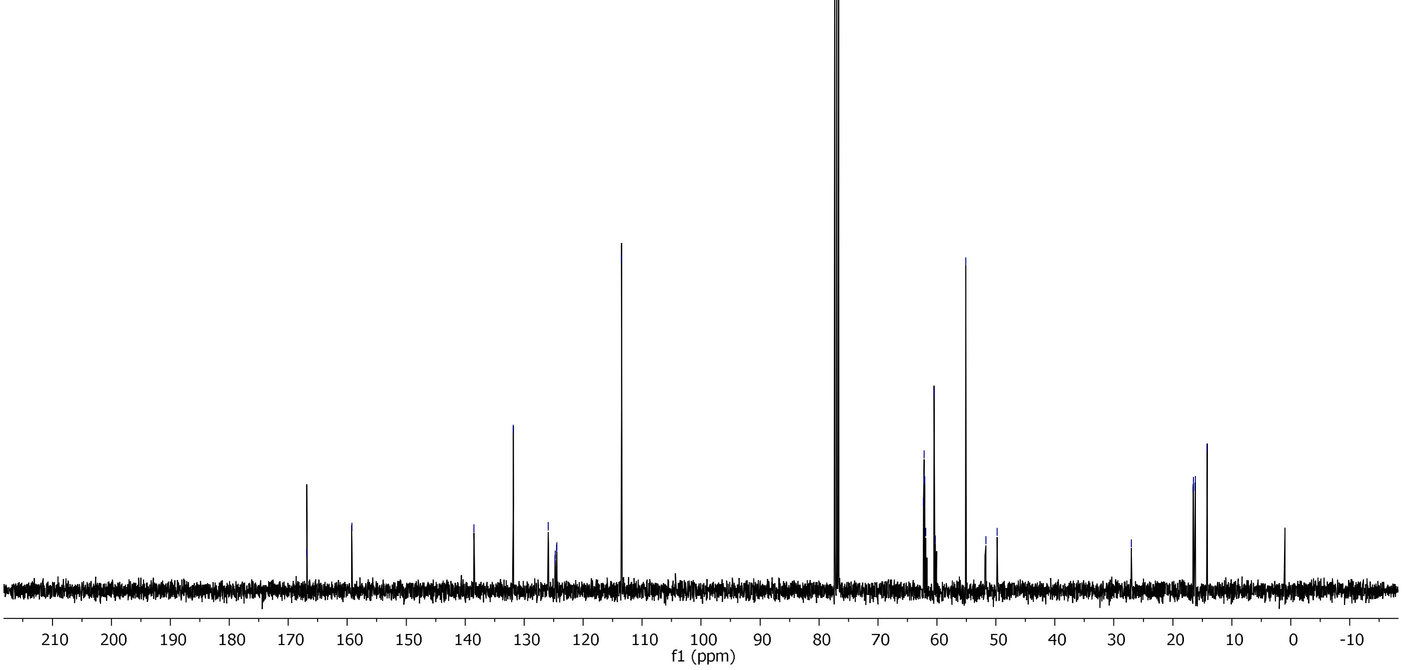

^{31}P NMR (162 MHz, $CDCl_3$) of compound **6e**.

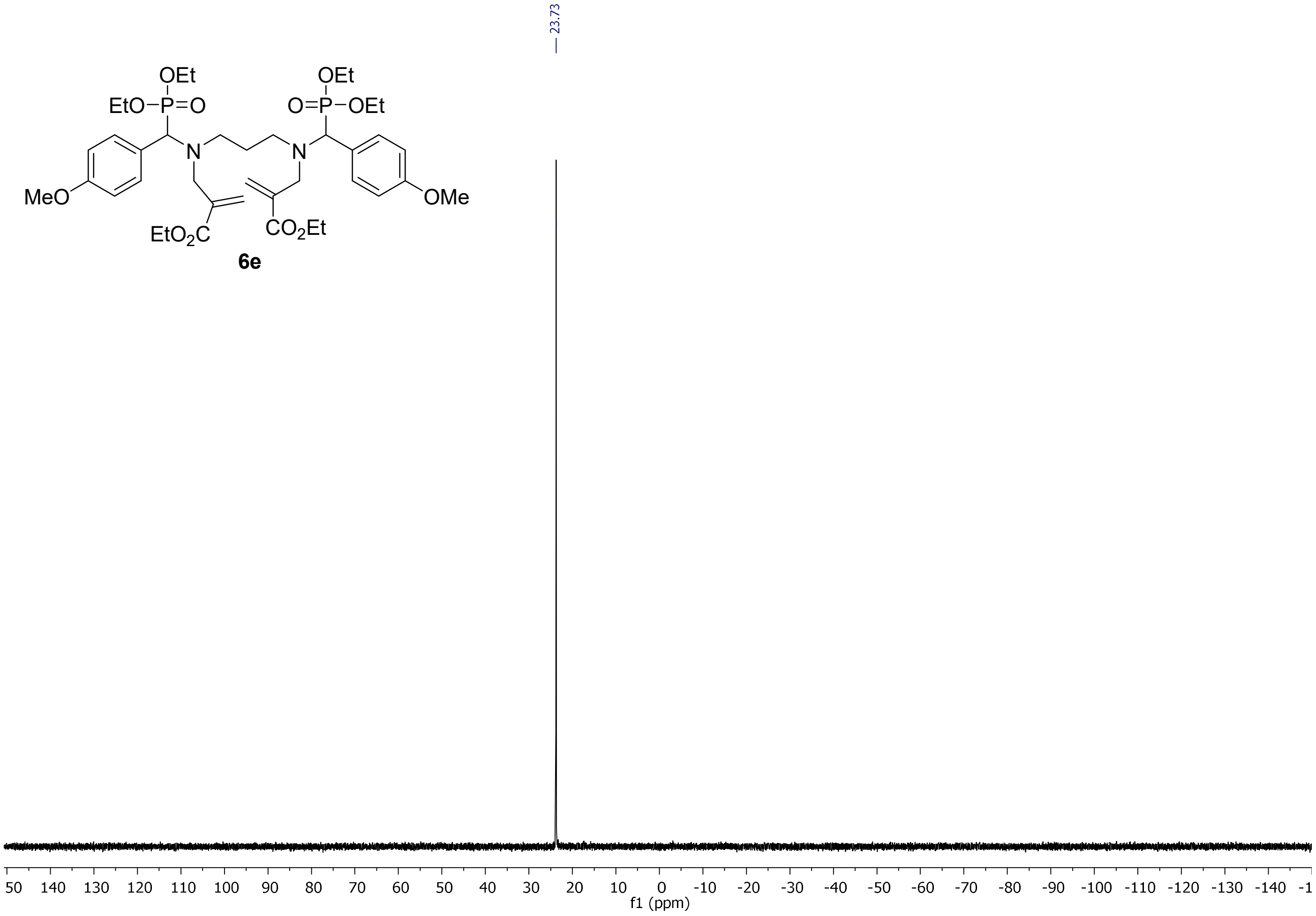

^{1}H NMR (300 MHz, $CDCl_3$) of compound **6f**.

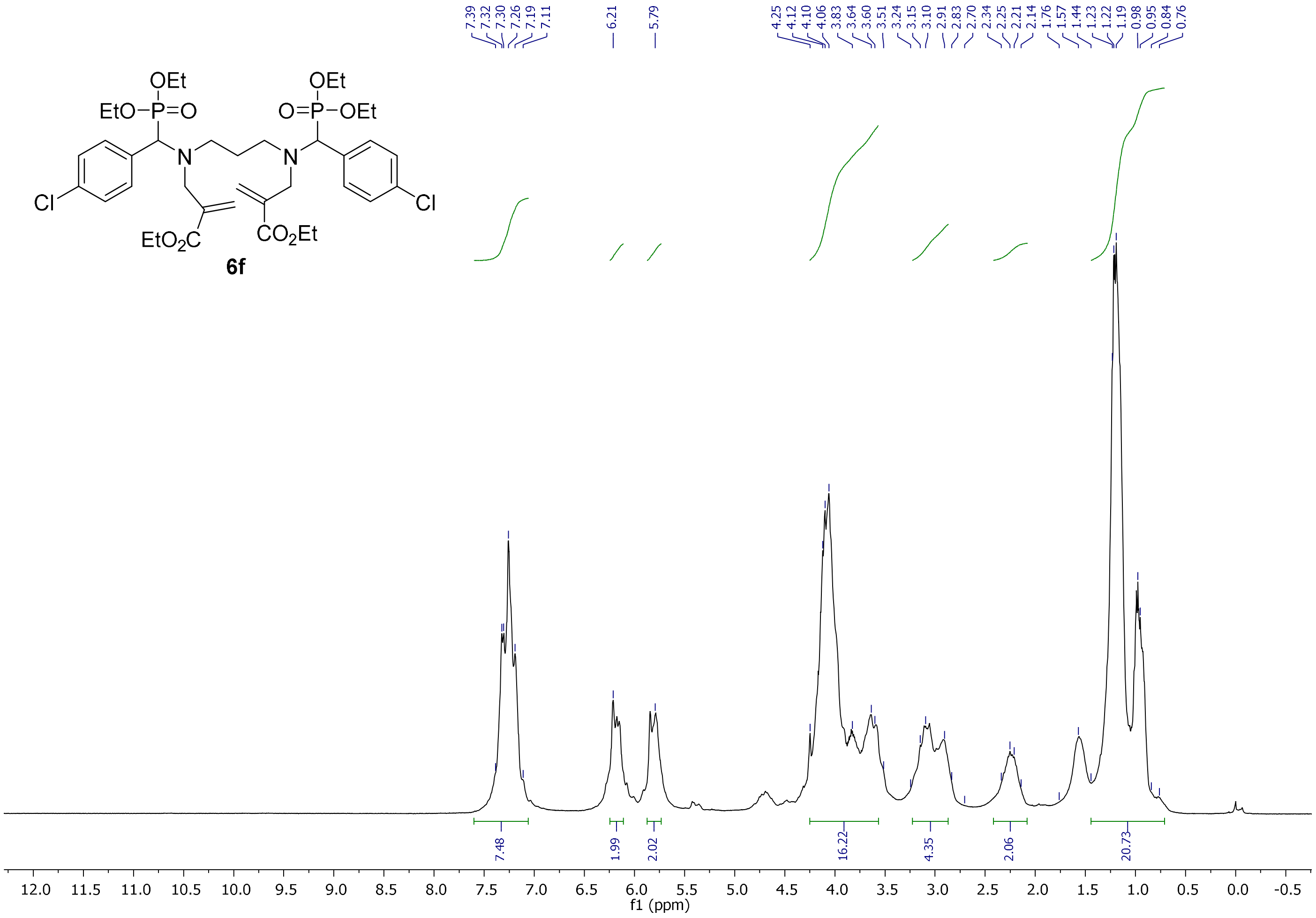

^{13}C {^{1}H} NMR (75 MHz, $CDCl_3$) of compound **6f**.

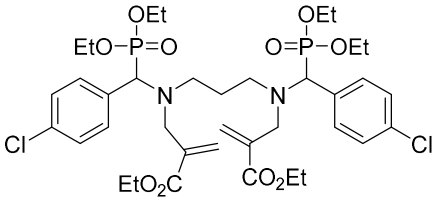

6f

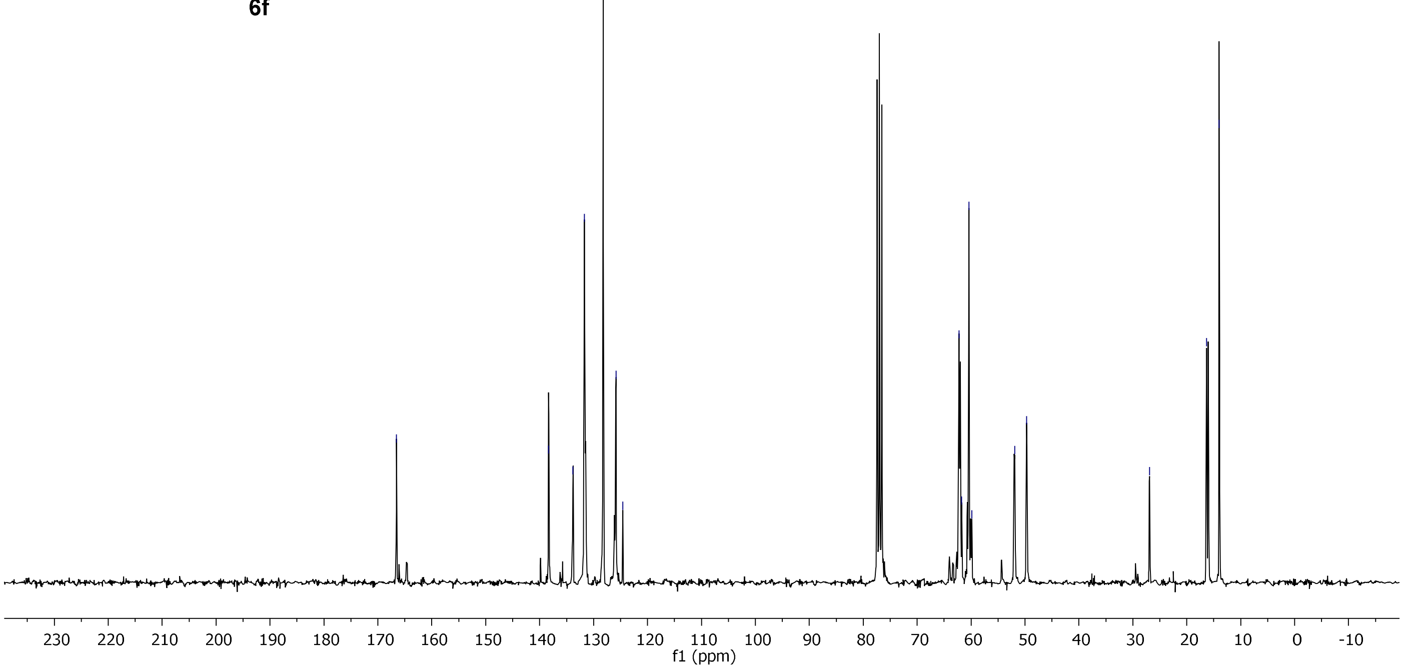

^{31}P NMR (121 MHz, $CDCl_3$) of compound **6f**.

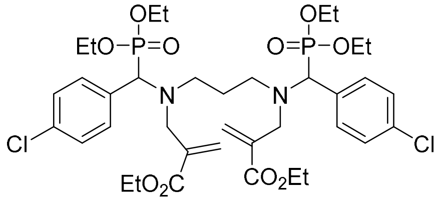

6f

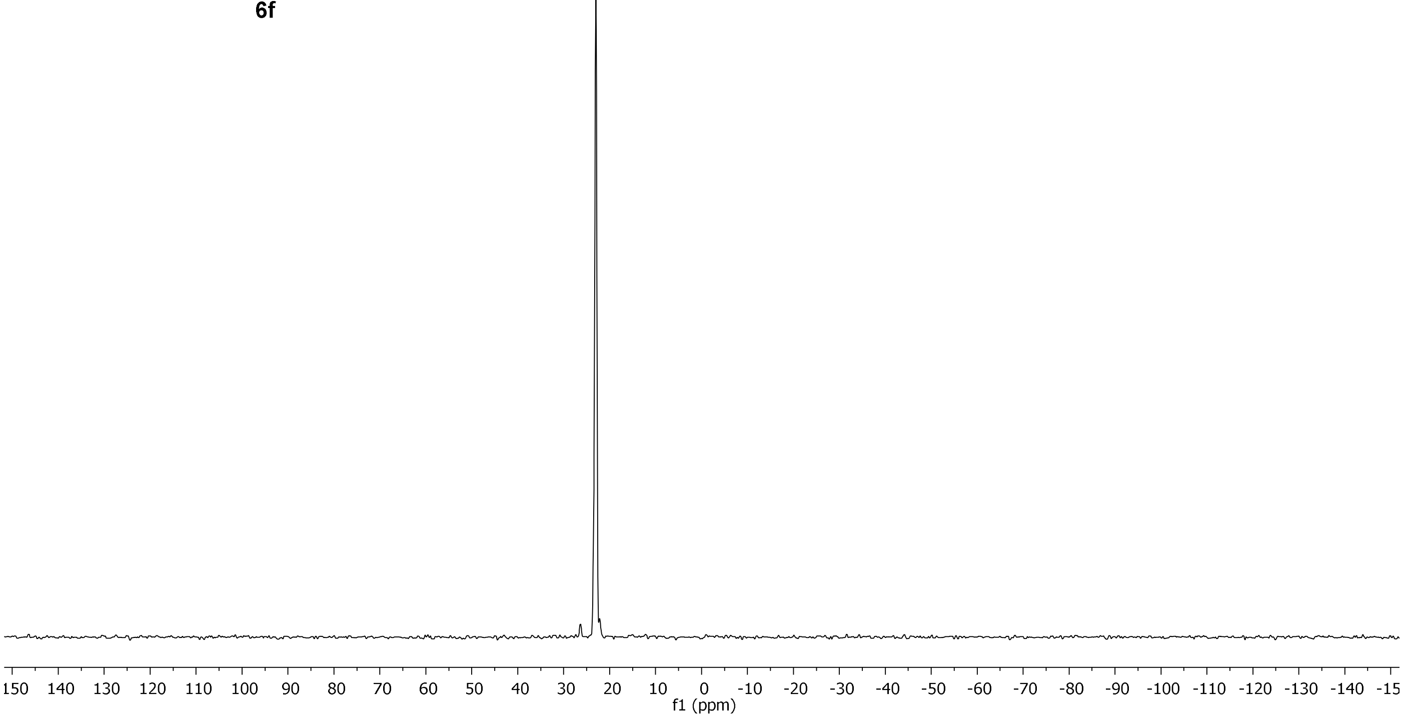

^{1}H NMR (400 MHz, $CDCl_3$) of compound **6g**.

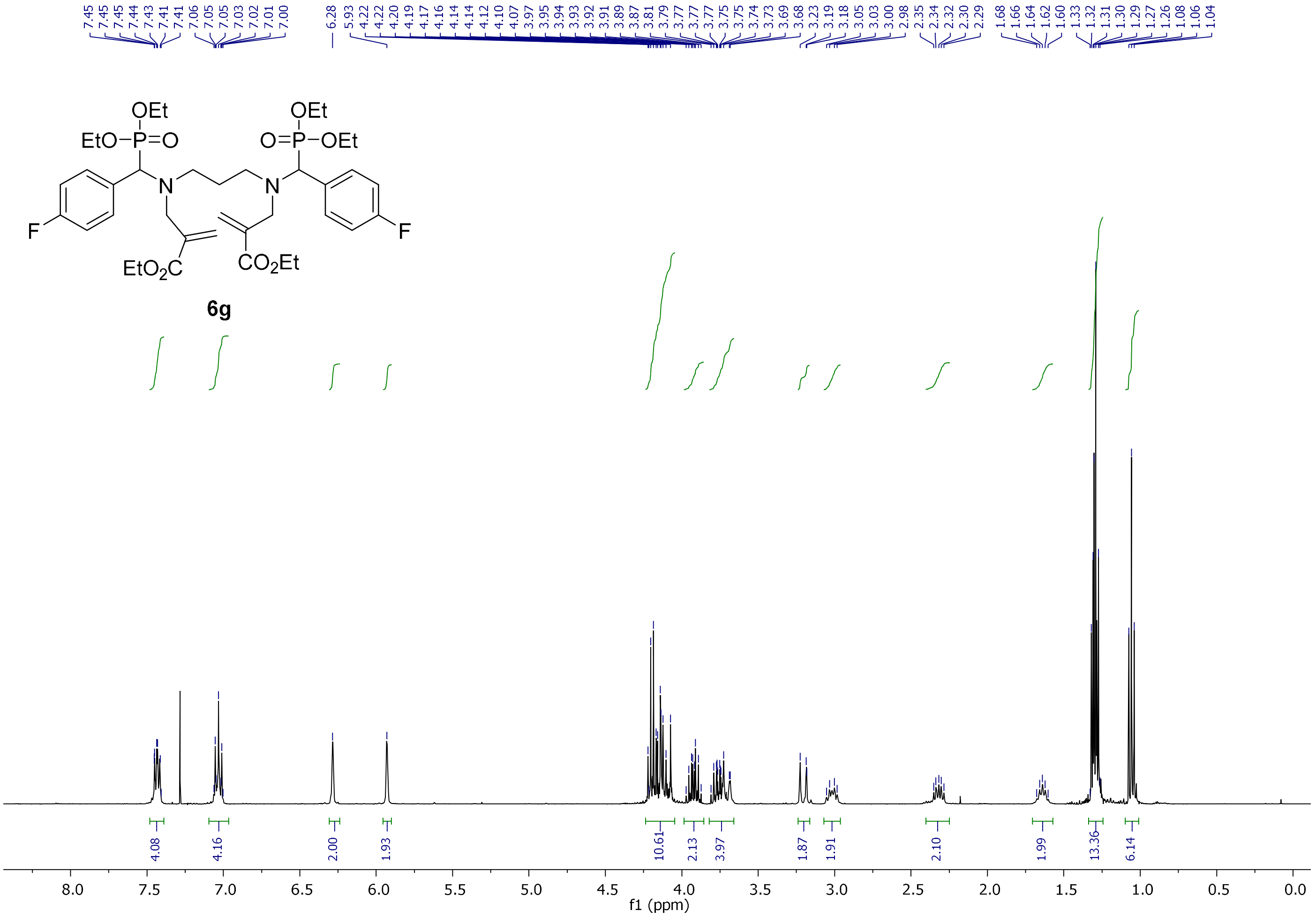

^{13}C {^{1}H} NMR (101 MHz, $CDCl_3$) of compound **6g**.

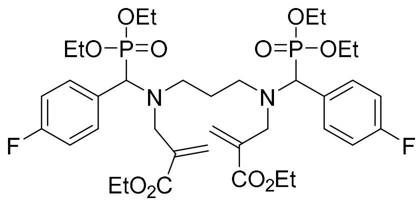

6g

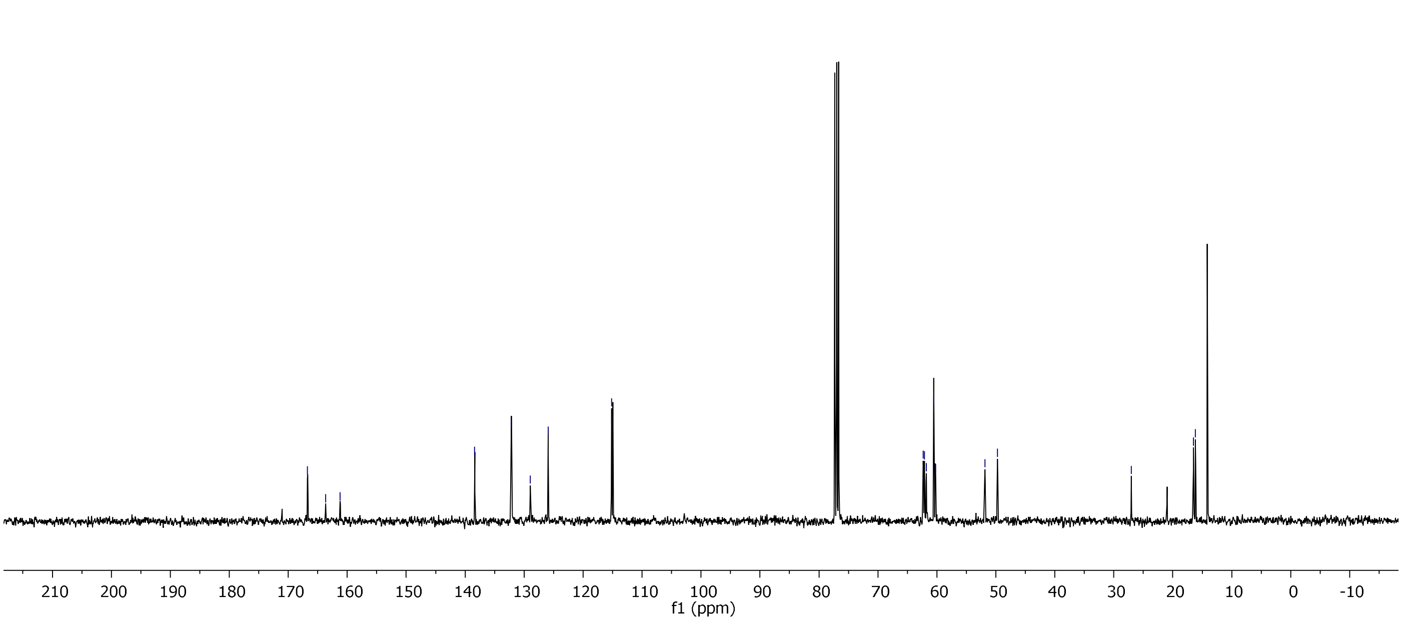

^{31}P NMR (162 MHz, $CDCl_3$) of compound **6g**.

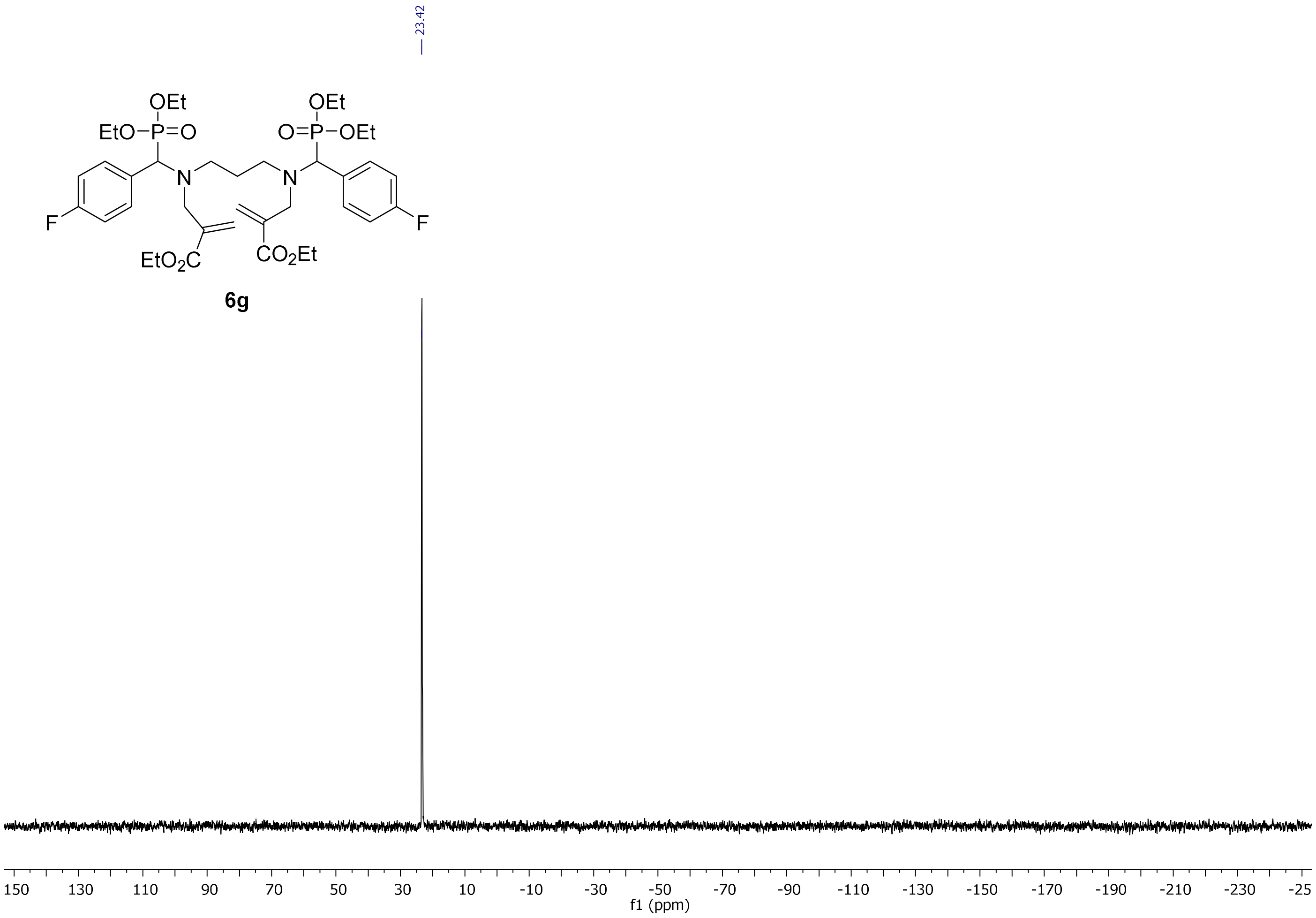

^{19}F NMR (376 MHz, $CDCl_3$) of compound **6g**.

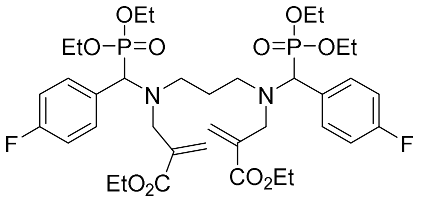

6g

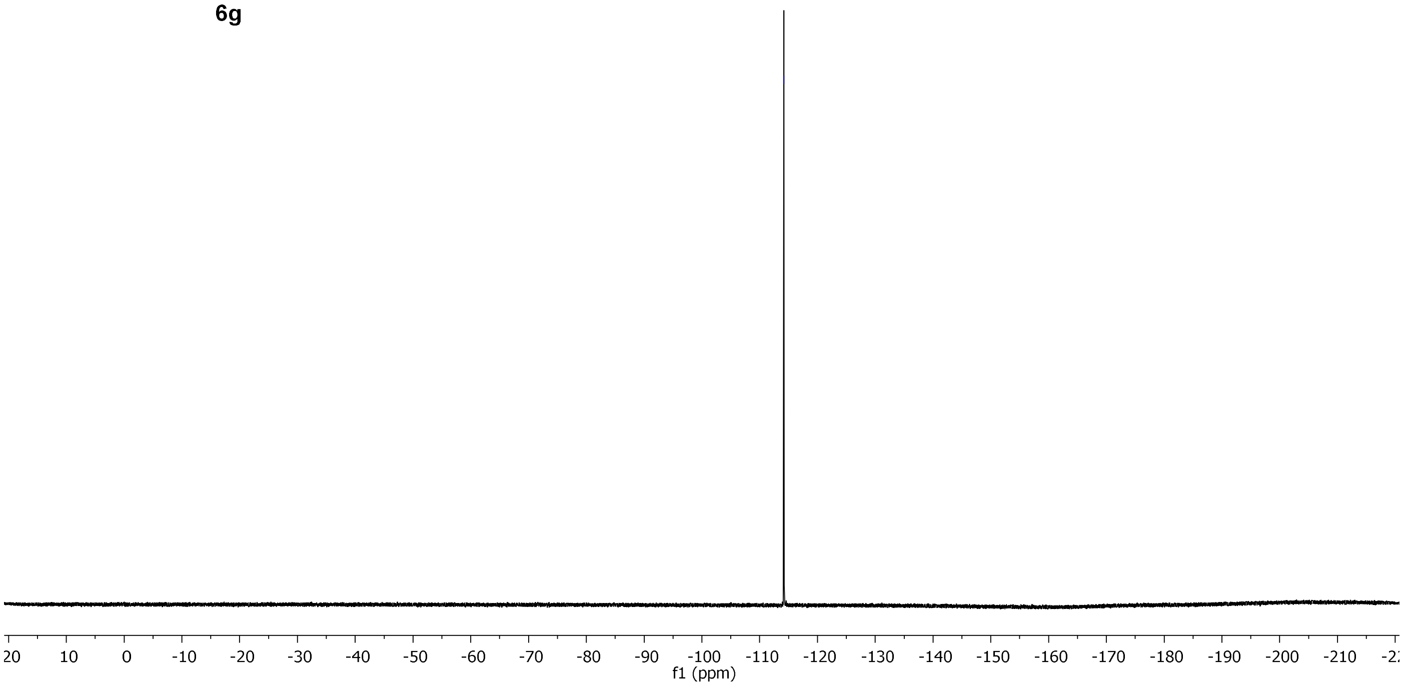

2D-COSY NMR {^1H-^1H} (400 MHz, $CDCl_3$) of compound **6g**.

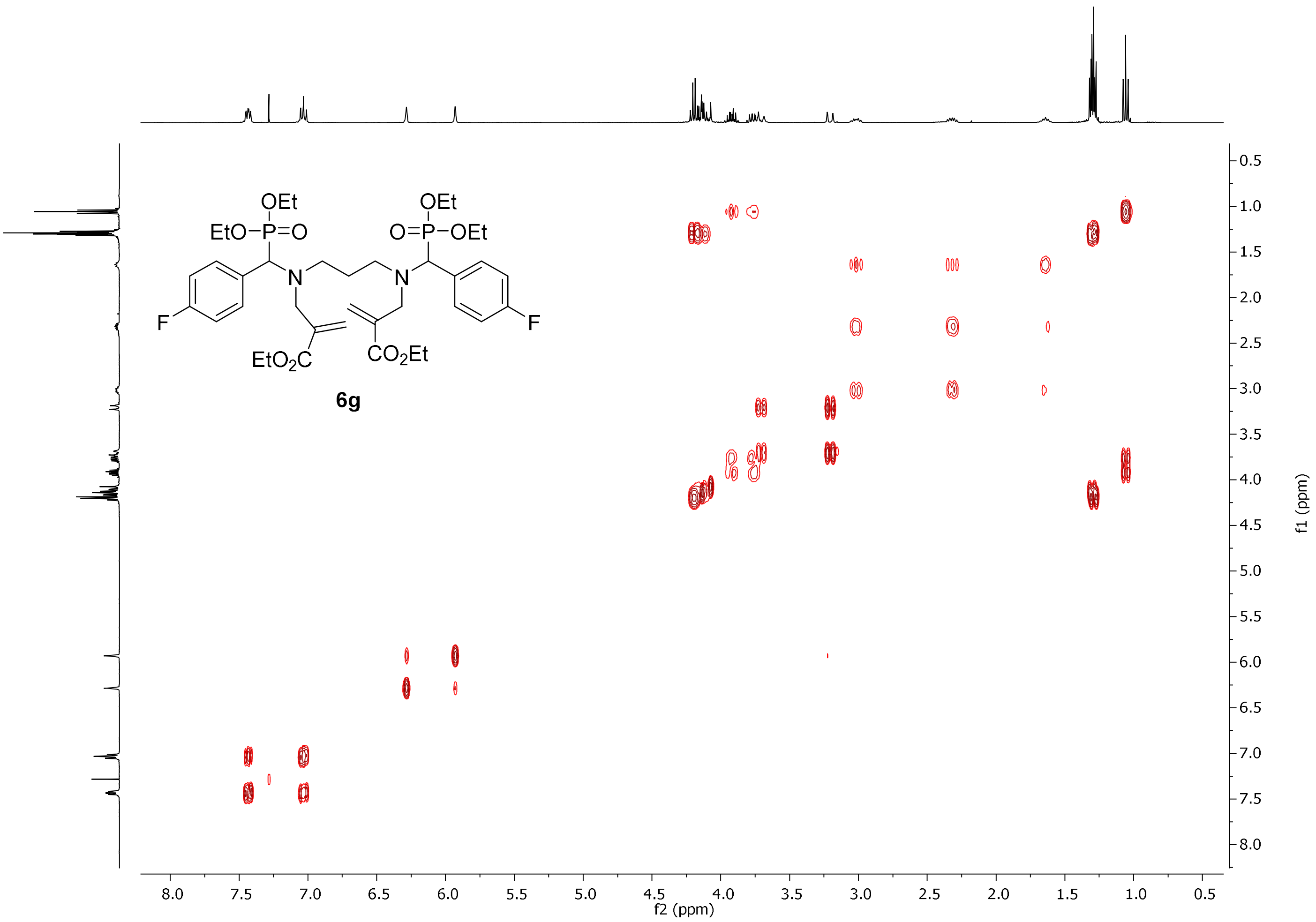

2D-HSQC NMR {^{1}H-^{13}C} (^{1}H: 400 MHz, ^{13}C: 101 MHz, $CDCl_3$) of compound **6g**.

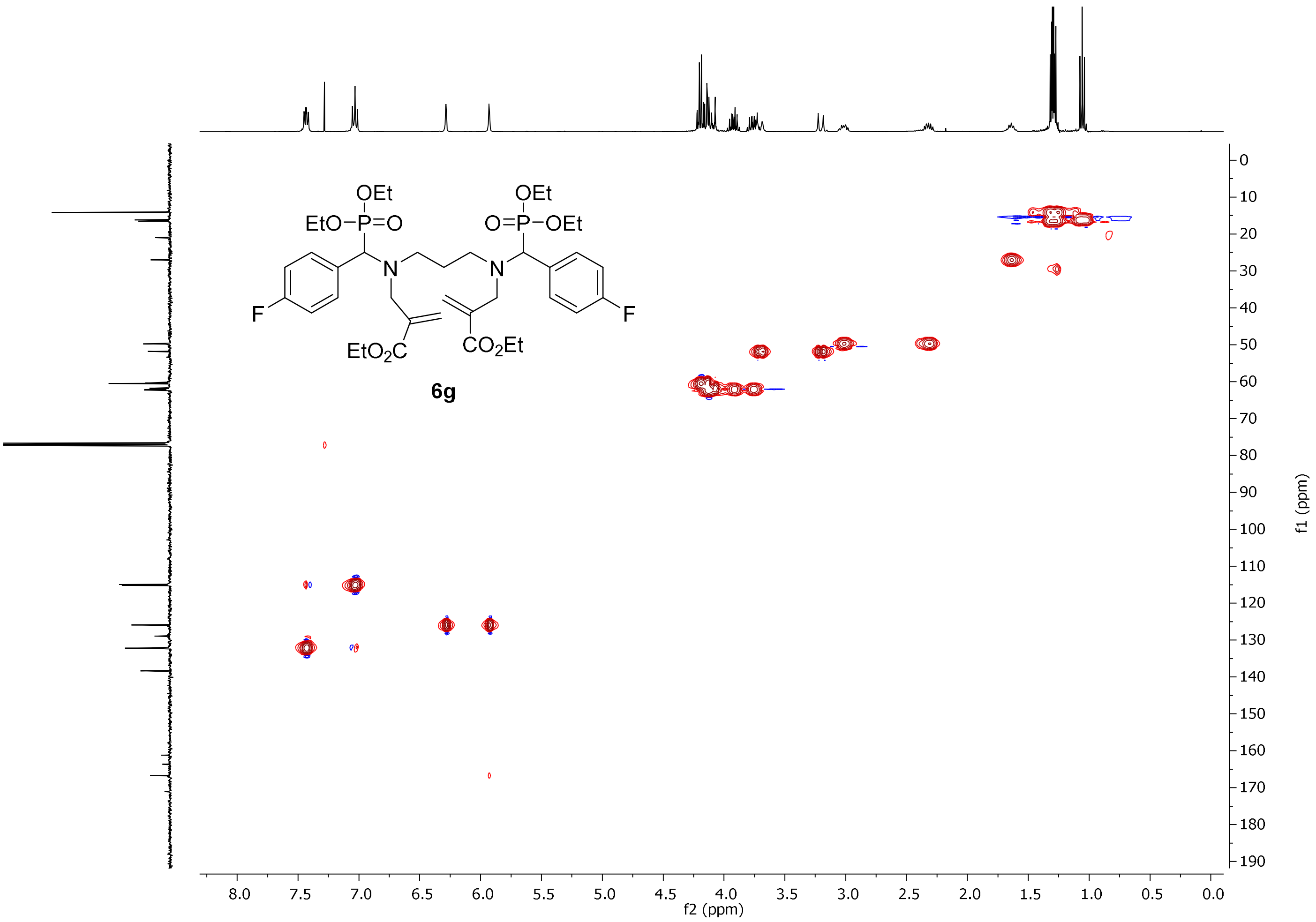

2D-HMBC NMR {^{1}H-^{13}C} (^{1}H: 400 MHz, ^{13}C: 101 MHz, $CDCl_3$) of compound **6g**.

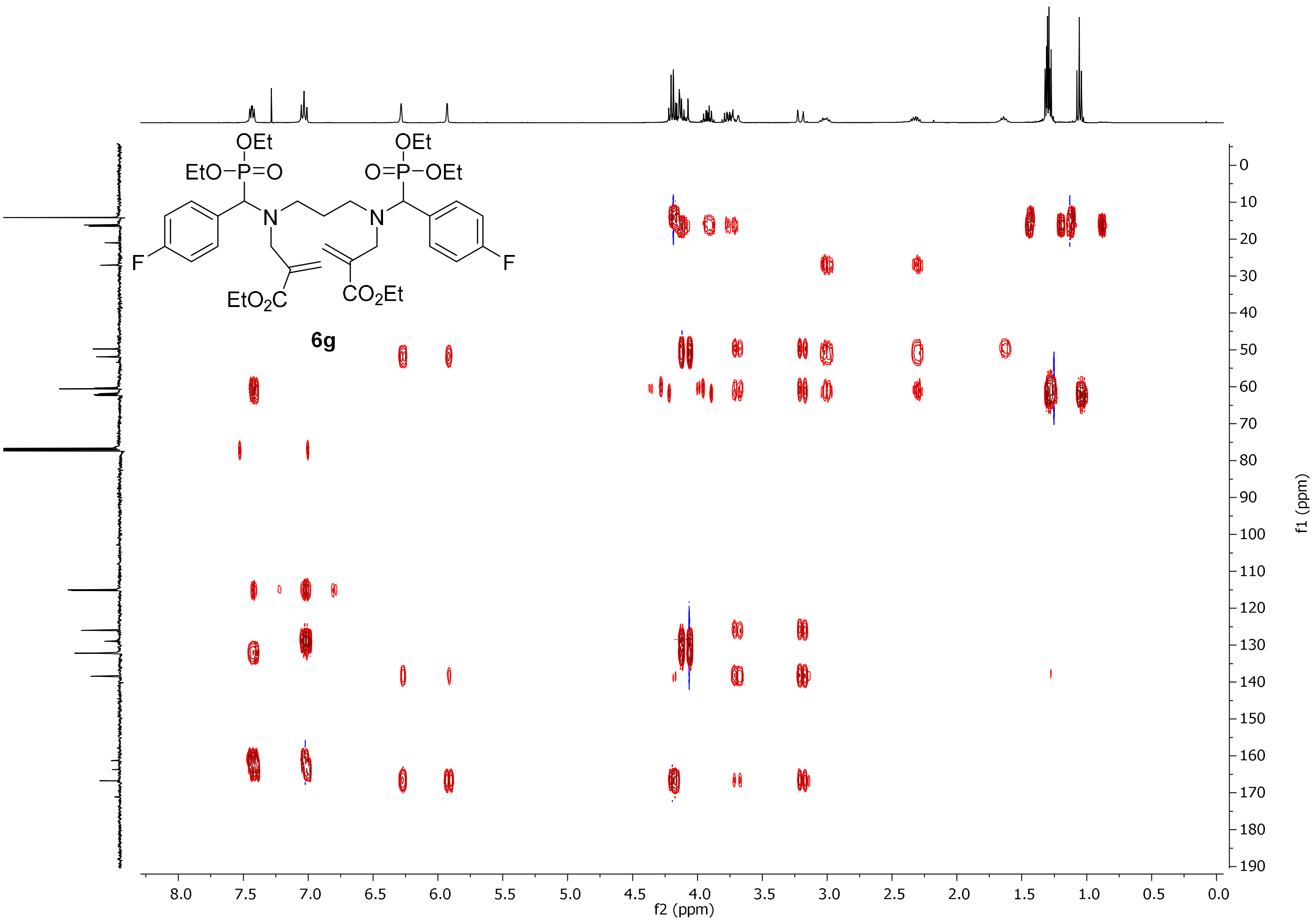

^{1}H NMR (400 MHz, $CDCl_3$) of compound **6h**.

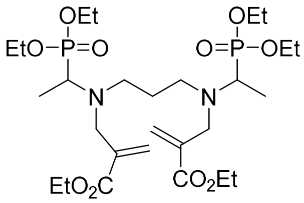

6h

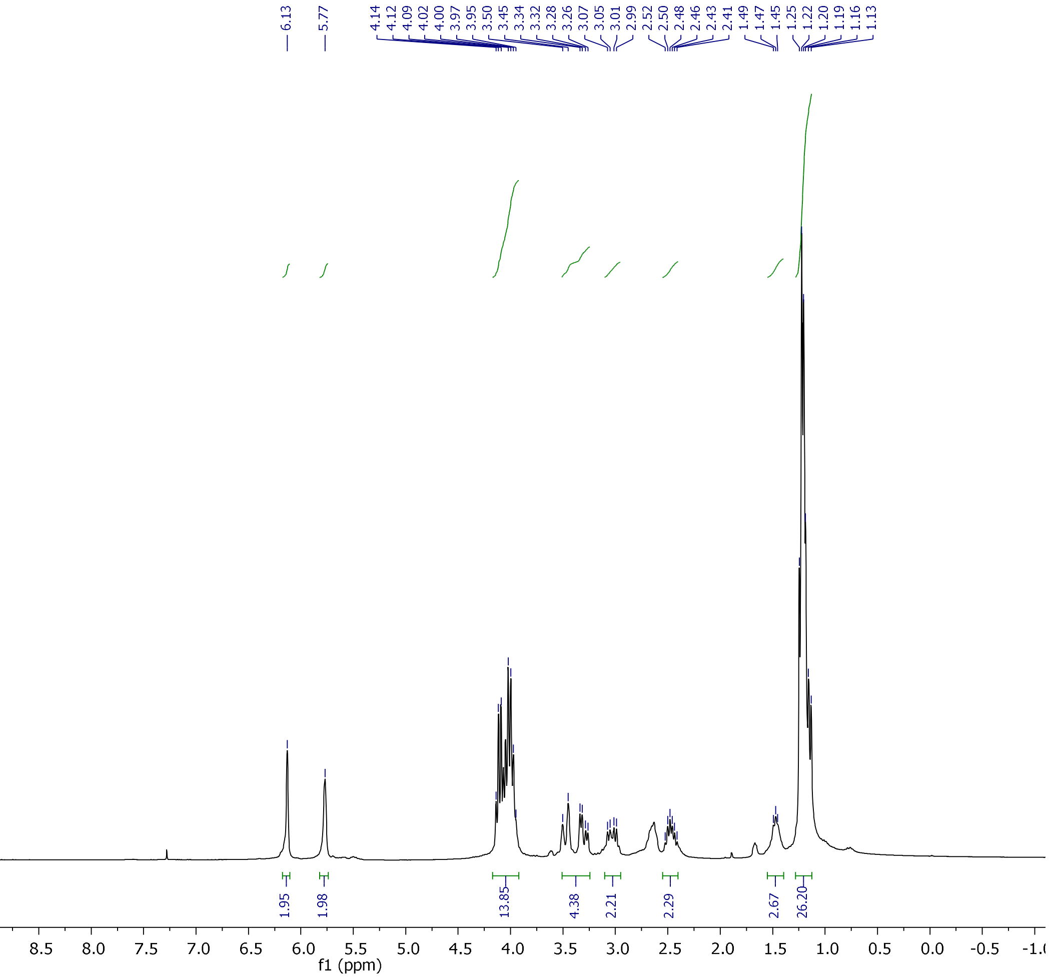

^{13}C {^1H} NMR (101 MHz, $CDCl_3$) of compound **6h**.

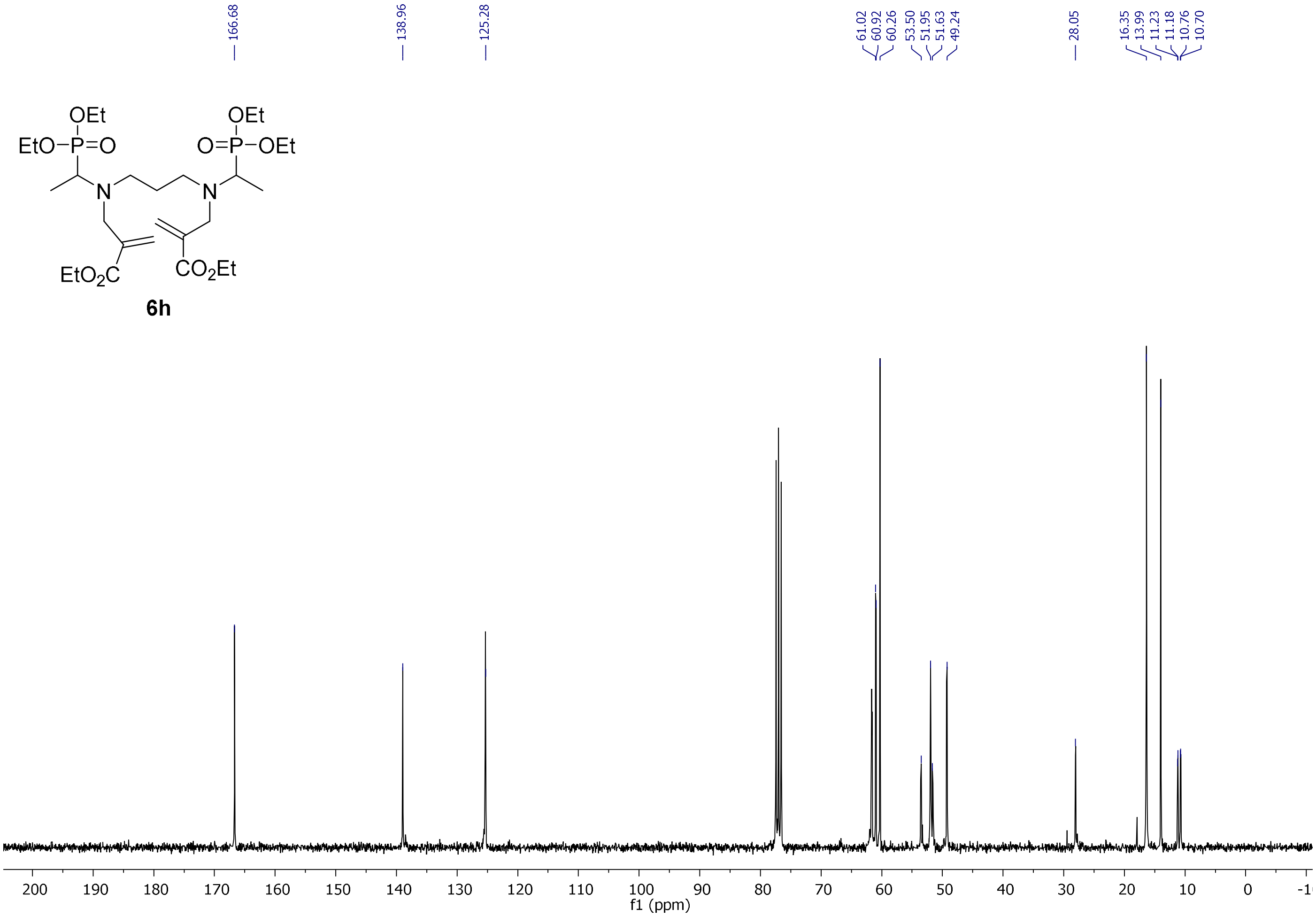

^{31}P NMR (162 MHz, $CDCl_3$) of compound **6h**.

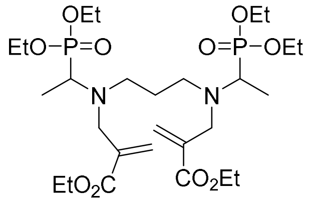

6h

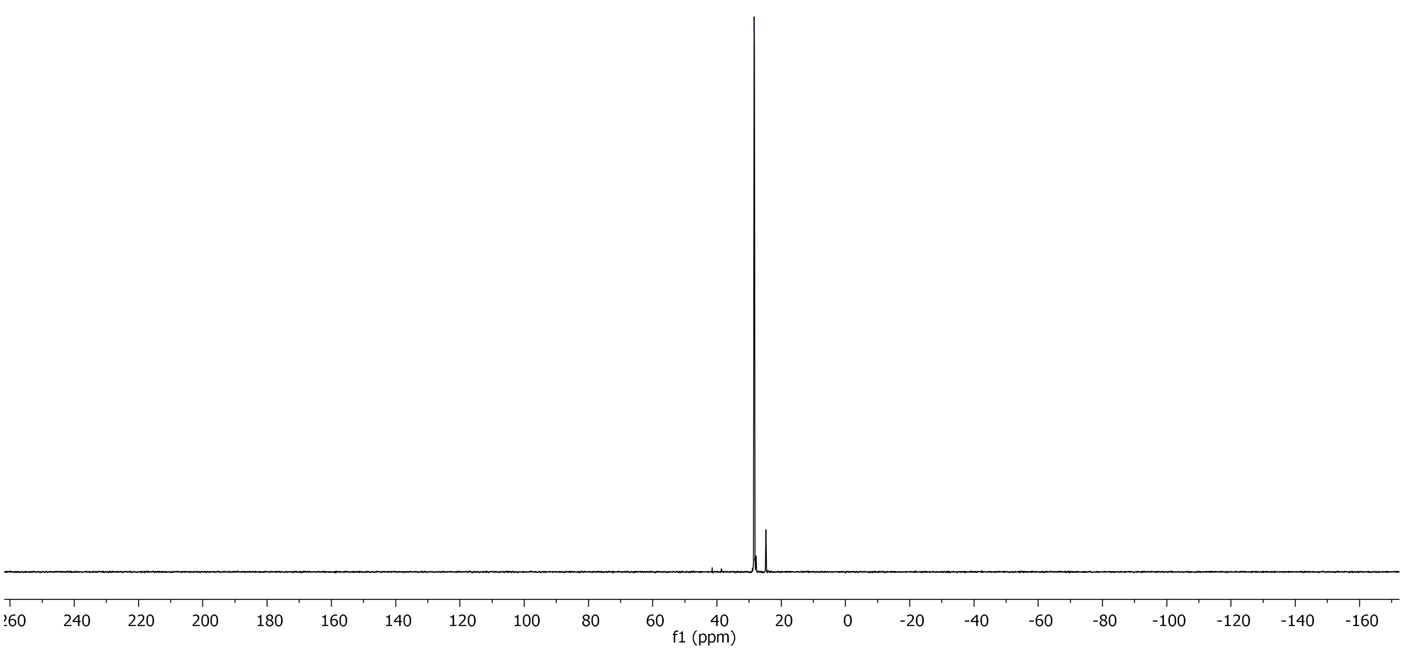

Supplement: Supplementary file 1 [file molecules-28-04678-s001.zip › molecules-2406878-supplementary.pdf]
